# The Candidate Phylum *Poribacteria* by Single-Cell Genomics: New Insights into Phylogeny, Cell-Compartmentation, Eukaryote-Like Repeat Proteins, and Other Genomic Features

**DOI:** 10.1371/journal.pone.0087353

**Published:** 2014-01-31

**Authors:** Janine Kamke, Christian Rinke, Patrick Schwientek, Kostas Mavromatis, Natalia Ivanova, Alexander Sczyrba, Tanja Woyke, Ute Hentschel

**Affiliations:** 1 Department of Botany II, Julius-von-Sachs Institute for Biological Sciences, University of Wuerzburg, Wuerzburg, Germany; 2 Department of Energy Joint Genome Institute, Walnut Creek, California, United States of America; 3 Center for Biotechnology, Bielefeld University, Bielefeld, Germany; University of Vienna, Austria

## Abstract

The candidate phylum *Poribacteria* is one of the most dominant and widespread members of the microbial communities residing within marine sponges. Cell compartmentalization had been postulated along with their discovery about a decade ago and their phylogenetic association to the *Planctomycetes*, *Verrucomicrobia*, *Chlamydiae* superphylum was proposed soon thereafter. In the present study we revised these features based on genomic data obtained from six poribacterial single cells. We propose that *Poribacteria* form a distinct monophyletic phylum contiguous to the PVC superphylum together with other candidate phyla. Our genomic analyses supported the possibility of cell compartmentalization in form of bacterial microcompartments. Further analyses of eukaryote-like protein domains stressed the importance of such proteins with features including tetratricopeptide repeats, leucin rich repeats as well as low density lipoproteins receptor repeats, the latter of which are reported here for the first time from a sponge symbiont. Finally, examining the most abundant protein domain family on poribacterial genomes revealed diverse phyH family proteins, some of which may be related to dissolved organic posphorus uptake.

## Introduction

Single-cell genomics is a powerful tool to describe genomes of as yet uncultivated organisms from diverse environments [1], [2]. Recently it allowed a first glimpse into the vast functional diversity represented by genomes of previously largely uncharacterized candidate phyla [3]. This method further revealed the glycobiome of the candidate phylum *Poribacteria*, symbionts of marine sponges, based on six single-amplified genome (SAG) sequences [4]. In this study we further examined these SAGs for phylogenetic and additional functional features of *Poribacteria*. *Poribacteria* were first discovered as highly abundant symbionts of marine sponges [5] and as of now lack any cultivated representatives. Through amplicon sequencing studied based on 16S rRNA genes they were also detected in seawater albeit in low abundances [6]–[8]. *Poribacteria* are one of the most predominant taxa inhabiting the extracellular matrix (mesohyl) of sponge species around the world [9]–[11]. These symbionts are vertically transmitted over larval stages from the adult sponge to the next generation [7], [12]. Initially, the candidate phylum *Poribacteria* showed a moderate phylogenetic relationship to *Planctomycetes*, *Verrucomicrobia*, and *Chlamydiae* (PVC superphylum) based on monophyletic clustering in 16S rRNA gene analysis [5]. Later, *Poribacteria* were classified as members of the PVC superphylum although the exact position within the superphylum could not be completely resolved [13]. Similar to some members of the PVC superphylum *Poribacteria* were also suspected to have a compartmentalized cell plan [5]. In this study we revisited the features of phylogeny and cell compartmentalization based on the sequence data of six single-cell derived genomes from the candidate phylum *Poribacteria*. We further reveal a large abundance and diversity of eukaryote-like domain containing proteins as well as phyH-like proteins in *Poribacteria*.

## Materials and Methods

### Genome Annotation and Analysis

Six poribacterial single-cell genome sequences were included in this study, these being *Candidatus Poribacteria* WGA 3A, 3G, 4C, 4CII, 4E and 4G with Genbank accession numbers ADFK02000000, ASZN01000000, APGO01000000, ASZM01000000, AQTV01000000, AQPC01000000, respectively. These genomes were previously obtained by our group from uncultivated bacteria inhabiting the marine sponge *Aplysina aerophoba* by fluorescence activated cell sorting (FACS), multiple displacement amplification (MDA), and next generation sequencing [14], [4].

Please also note that the initial version of genome WGA 3A (first published as WGA A3 with accession number ADFK00000000 version ADFK01000000) [14] was found to be flawed. It was corrected accordingly and the submission to Genbank was updated (version ADFK02000000) [4]. All genomic information of WGA 3A in this manuscript is based on the latest version of the genome, which should be used for all future studies. For a detailed description of all steps from sample collection to genome assembly and annotation please refer to Kamke et al. [4]. Genome sequences were automatically annotated via the IMG pipeline [15] and manually curated in IMG/MER. All analyses were conducted using the tools in IMG/MER unless further specified.

#### Clustering analysis of PhyH family genes

For clustering of pfam 05721-PhyH family proteins we used the fastclust algorithm in usearch [16] with an identity cutoff of 60% amino acidid.

### Phylogenetic 16S rRNA Gene Analysis

Sequences for 16S rRNA gene based phylogenetic analysis were selected from the SILVA 16S rRNA database version 108 [17] in the ARB software package (V5.3) [18]. All poribacterial 16S rRNA sequences (≥1100 bp) available in GenBank by June 2013 and the 16S rRNA sequences of poribacterial single-cell genomes were included. Additional sequences for the candidate phyla *Aerophobetes* (CD12) and *Hydrogenedentes* (NKB19) were obtained by blast searches [19] of reference sequences (accession number JN675971 for CD12 and CR933119 for NKB19) against Genbank nr/nt database in June 2013 and selecting the 100 best hits with >75% sequence ID and sequence length ≥1100 bp. All sequence added to the original database were aligned using the SINA aligner [20] and included into the ARB database for further manual refinement. Alignments were exported from ARB for phylogenetic tree construction using RAxML (v7.3.2) [21]. Maximum likelihood trees were constructed using sequences ≥1100 bp only and 50% conservation filters. Bootstrap analysis was carried out with 500 resamplings. Trees were reimported into ARB and sequences <1100 bp were added to the tree using the parsimony interactive tool in ARB without changing tree topology.

### Phylogenetic Analysis of 83 Bacterial Marker Protein Sequences

For the calculation of the bacterial phylogenetic tree we followed the procedure described by Rinke et al. [3] based on a custom marker set of 83 bacteria specific markers (Table S1) described in the study. Briefly, single-cell genome assemblies of *Poribacteria* were translated into all six reading frames and marker genes were detected and aligned with hmmsearch and hmmalign included in the HMMER3 package [22] using HMM profiles obtained from phylosift (http://phylosift.wordpress.com/). Extracted marker protein sequences were used to build concatenated alignments of up 83 markers per genome. Alignments were included into the database constructed by Rinke and coworkers [3] and reference sequences were selected for phylogenetic tree construction. Phylogenetic inference methods used were the maximum likelihood based FastTree2 [23] and a custom RAxML bootstrap script originally provided by Christian Goll and Alexandros Stamatakis (Scientific Computing Group, Heidelberg Institute for Theoretical Studies, Germany) and modified by Douglas Jacobsen (Bioinformatics Computing Consultant, LBNL, Berkeley, USA). The script requires two input files, the alignment file as PHYLIP format and a starting tree calculated by RAxML-Light [24]. The script workflow is briefly summarized as follows: First RAxML version 7.3.5 [21] creates bootstrap replicates of the multiple sequence alignments and stepwise addition order parsimony trees as starting points for the maximum likelihood search, based on user defined rate heterogeneity and substitution models. Next RAxML-Light [24] is run on every bootstrap replicate. After all RAxML-Light runs are finished the resulting replicate trees are fed into RAxML to calculate the bootstrap support values which are drawn upon the starting tree. The rate heterogeneity and amino acid evolution models used were GAMMA and LG for the custom RAxML bootstrap script, and CAT approximation with 20 rate categories and Jones-Taylor-Thorton (JJT) for FastTree2. To evaluate the robustness of the protein trees we used seven different out-group taxon configurations (Table 1).

**Table 1:** Summary of phylogenetic inference results from all phylogenomic tree calculations.

Inference^1^	Species^2^	Por BS^3^	Sistergroup^4^	Clade members (BS)^5^	Outgroup^6^
Fasttree, CAT, JTT	2311	100%	Hydrogenedentes (NKB19)	Poribacteria, Hydrogenedentes (NKB19), Aerophobetes (CD12) (100%)	all bacteria
Fasttree, CAT, JTT	316	100%	Aerophobetes (CD12)+Hydrogenedentes(NKB19)	Poribacteria, Hydrogenedentes (NKB19), Aerophobetes (CD12) (100%)	Spirochaetes, Alpha- & Betaproteobacteria, Firmicutes, Cyanobacteria, Elusimicrobia
Fasttree, CAT, JTT	310 (noS)	100%	Chloroflexi	Poribacteria, Chloroflexi, Hydrogenedentes (NKB19), Aerophobetes (CD12) (87%)	Spirochaetes
Fasttree, CAT, JTT	312 (noS)	100%	Hydrogenedentes (NKB19)	Poribacteria, Hydrogenedentes (NKB19), Aerophobetes (CD12), Elusimicrobia (71%)	Spirochaetes, Alpha-, Beta-, & Gammaproteobacteria
Fasttree, CAT, JTT	306 (noS)	100%	Aerophobetes (CD12)	Poribacteria, Aerophobetes(CD12) (100%)	Spirochaetes
RAxML, GAMMA, LG	312 (noS)	100%	Hydrogenedentes (NKB19)	Poribacteria, Hydrogenedentes (NKB19), Aerophobetes (CD12), Elusimicrobia (45%)	Spirochaetes, Alpha-, Beta-, & Gammaproteobacteria

1 Inference method, rate categories, and substitution model.

2 number of species in tree. Single sequences which did not belong to any main clades were removed before tree calculations where indicated (noS = no Singletons).

3 Bootstrap support for the phylum Poribacteria.

4 sistergroup to the phylum Poribacteria.

5 sistergroup to the phylum Poribacteria.

6 phyla added as outgroups for tree calculation.

## Results and Discussion

### Phylogenetic Revision of *Poribacteria*

**Figure 1:**
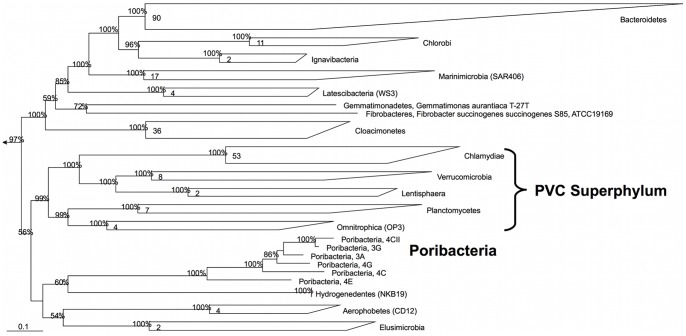
Phylogenomic tree based on a concatenated alignment of up to 83 genes illustrating the phylogenetic position of the candidate phylum *Poribacteria*. Bootstrap value (100 resamplings) are shown on tree nodes where support ≥50%. Number of genomes per group is displayed in group boxes. Outgroup consists of several species of *Spirochaetes* and *Gammaproteobacteria*. The scale bar represents 10% sequence divergence.

**Figure 2:**
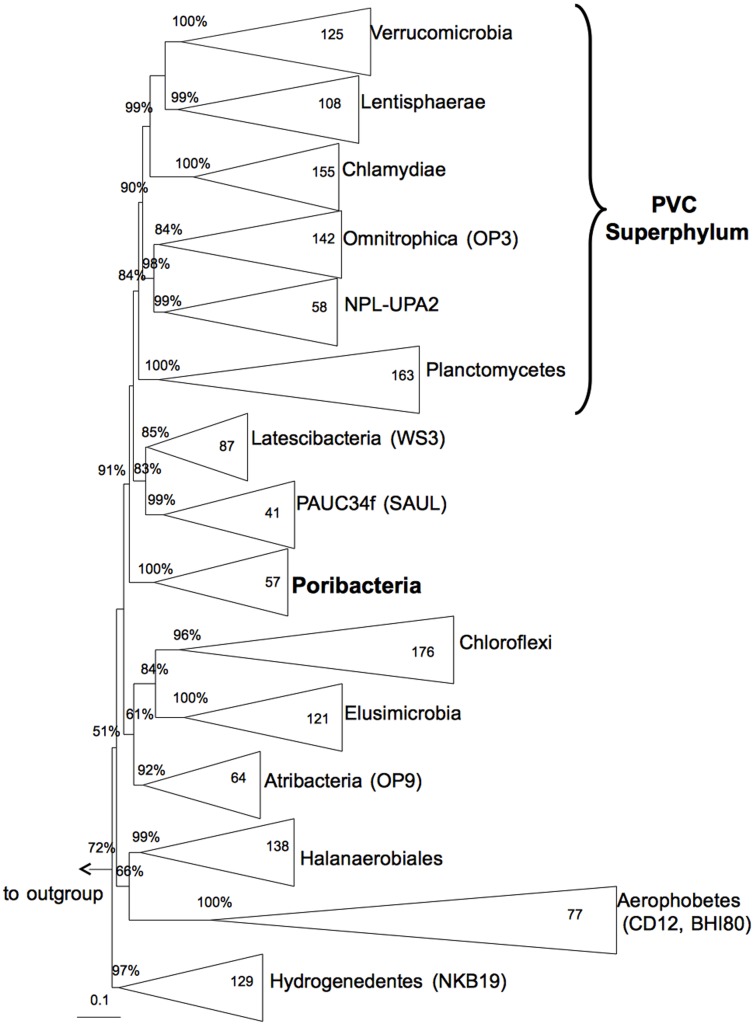
16S rRNA gene based maximum likelihood tree illustrating the phylogenetic position of the candidate phylum *Poribacteria*. Bootstrap values (500 resamplings) ≥50% are shown on tree nodes. Numbers of sequences included per group is shown in group boxes. Outgroup consists of 80 sequences belonging to the *Bacteroidetes*. Scale bar represents 10% sequences divergence.

Analysis of phylogenetic interferences of up to 83 marker genes (hereafter termed phylogenomic analyses) showed that all poribacterial SAGs clustered, with 100% bootstrap support in all our tree calculations, in a monophyletic group distinct to the PVC superphylum (Table 1, Fig. 1). *Poribacteria* SAGs clustered with the recently proposed phyla *Aerophobetes* (CD12) and/or *Hydrogenedentes* (NKB19) [3] in most of our phylogenomic calculations (Table 1). This loosely affiliated clade, including other phyla such as *Elusimicrobia*, formed in some tree calculations a sister clade to the PVC superphylum (Fig. 1). Phylogenetic analysis of the 16S rRNA gene supported monophyletic clustering of *Poribacteria* with strong bootstrap support (Fig. 2). However, phylogenetic placement based on the 16S rRNA gene did not show the direct grouping with *Aerophobetes* (CD12) and/or *Hydrogenedentes* (NKB19) (Fig. 2). Instead *Poribacteria* were placed (bootstrap support 91%) separately within a larger cluster of other phyla including the PVC superphylum as well as the candidate phylum WS3, recently renamed as *Latescibacteria* [3] and a monophyletic lineage previously described as “sponge associated unclassified lineage” (SAUL) [10].

The inconsistency between phylogenomic and the 16S rRNA gene-based phylogeny might be due to the relatively low resolution provided by the single marker gene (16S rRNA) analysis compared to multiple genes analysis as has been suggested previously [3], [25]. On the other hand the phylogenomic analysis, limited to the relatively small amount of draft reference sequences available at the time of analysis, might not be able to properly resolve the general placement of the phylum. We expect that the position of the *Poribacteria* in the tree of life will be further refined as more genome sequences of *Poribacteria* and of other candidate phyla become available. Importantly, the phylogenetic analyses performed in this study (whether 16S rRNA gene or marker genes based) did not support a clustering of *Poribacteria* with the PVC superphylum, which is in contrast to what was suggested earlier [13].

Previous studies based on concatenated alignments of protein data [26], [27] also showed the phylogenetic position of *Poribacteria* outside the PVC superphylum. However, these studies included only one poribacterial genome sequence available at that time, *Candidatus* Poribacteria sp. WGA A3 in its initial version (ADFK01000000). This version was later shown to be flawed by contaminating DNA and was replaced in Genbank (ADFK02000000) [4]. Since the previous studies examining poribacterial phylogeny [26], [27] were published before the release of the updated version they could not have revealed accurate placement of *Poribacteria*. Besides phylogenetic analysis, two marker proteins were described for members of the PVC superphylum [26], [28]. Blast searches using representatives sequences of these signature molecules [26], [28] as query against the poribacterial SAG sequences did not show the presence of any PVC marker. This lack of a PVC marker proteins provides further support for the independent phylogenetic position of *Poribacteria*.

### Genomic Evidence for Microcompartments

Cell compartmentalization is one characteristic that has been proposed for *Poribacteria* based on ring shaped fluorescence in situ hybridization (FISH) signals and the electron microscopic observations of compartmentalized prokaryotic cells in the mesohyl of the sponge *Aplysina aerophoba* [5]. The observed structures appeared similar to those described for many members of the *Planctomycetes* [29] and most specifically for *Gemmata obscuriglobus* where the compartment was proposed to be a nucleus-like structure [30]. The existence of cell compartmentalization in members of the PVC superphylum was later connected to the occurrence of membrane coat like proteins encoded on the genome [31]. To further investigate the possibility of cell compartmentalization in *Poribacteria*, we searched poribacterial SAGs for possible genomic evidence of such features. We were not able to find membrane coat like proteins or any genomic indication of large cell compartments. This is in accordance with a recent study which challenged the concept of the existence of these compartments even in other bacteria and confutes the existence of a nucleus-like structure in *G*. *obscuriglobus* [32].

**Figure 3:**
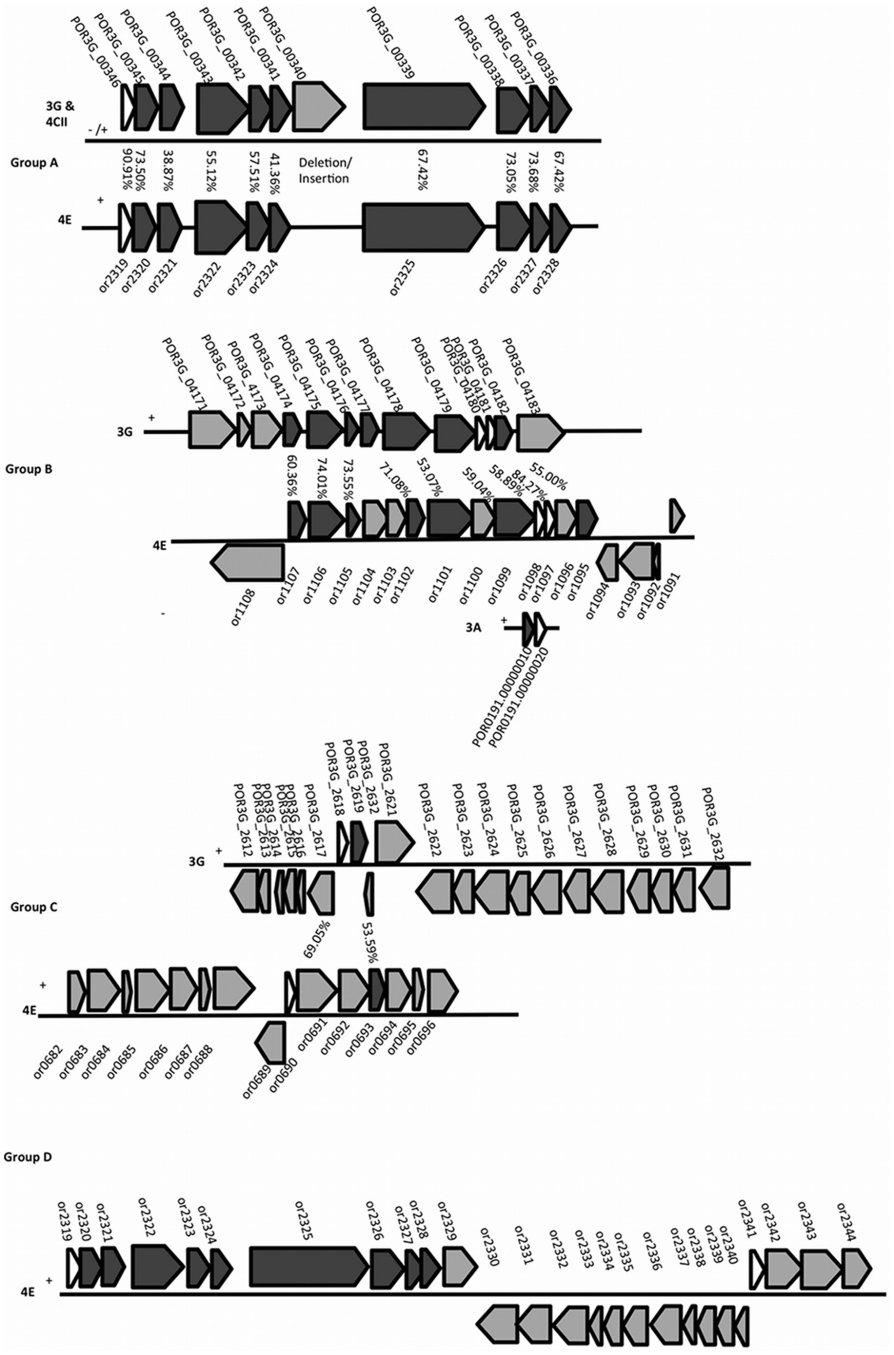
Schematic view of poribacterial BMC shell protein groups. For a better overview all genes are displayed in 5′-3′ direction of the BMC shell protein gene. The actual strand orientation might be different and is indicated by plus or minus signs. Genes are shown with locus taq and amino acid identities based on IMG/MER homology searches are shown between genes where applicable. BMC shell protein genes are shown in white, other genes with homologies between different SAGs are shown in dark grey, other genes are shown in light grey.

Our analysis did reveal evidence for a possible occurrence of bacterial microcompartments (BMCs) in *Poribacteria*. Four of six poribacterial SAGs encoded for genes with hits to either one of two pfam domains namely, pfam00936 BMC or pfam03319 EutN CcmL (Table 2). These domains are considered markers for BMC shell proteins. Specifically, we identified three regions with conserved genomic structure between different poribacterial SAGs (groups A-C) (Fig. 3) that encoded for genes with these domains. A fourth region (group D) was identified on SAG 4E with two BMC shell proteins enclosing a set of 21 genes (Fig. 3). A detailed description of these groups can be found in Text S1 and tables S2, S3, S4, and S5. BMCs are proteinaceous structures that enclose sets of enzymes of diverse functions performing a chain of reactions within the compartment [33]. BMC shell functions have been described as concentrating enzymes and substrates together to increase reaction efficiency, protection of e.g. oxygen sensitive enzymes, enclosure of toxic or volatile metabolites that are produced/consumed by enzymes in the shell, and concentrating metabolites to increase efficiency [33], [34]. Kerfeld et al. [33] suggested that at least two (or more) pfam00936 domain proteins and one pfam03319 domain protein might be required as building blocks of functional BMCs. Out of all poribacterial SAGs only 4E encoded for more than one pfam00936 domain and, with the exception of SAG 4CII, all poribacterial SAGs encoded for a higher number of pfam03319 than pfam00936 domains (Table 2). This is unusual when compared to most other BMC shell protein studied to date (Table S6). *Poribacteria,* together with *Planctomycetes*, the candidate phylum *Atribacteria* (OP9), and some additional phyla (Table S6), appear to be among the few exceptions containing more pfam03319 than pfam00936 domains.

**Table 2:** BMC shell protein markers on poribacterial SAGs.

Function ID	Name	3G	4CII	4E	3A
pfam00936	BMC	1	1	2	0
pfam03319	EutN CcmL	3	0	3	1

The so far best described BMC functions are the carboxysome and BMCs containing enzymes for ethanolamine or propanediol utilization. Bioinformatic analysis of all available BMC shell protein encoding genomic regions at the time by Yeates et al. [35] revealed that functional proteins within the BMCs are often encoded in close proximity of the BMC shell proteins and identified a set of functions regularly occurring with BMC shell proteins. However, the genes in poribacterial BMC clusters did not show direct similarities to any of these previously described functions but some genes in poribacterial BMC clusters give an indication of potential functions. It is noteworthy that many of the described enzymatic reactions in previously described BMCs are co-factor dependent (often vitamin B12), and that the co-factor biosynthesis genes were often found in close proximity to BMC shell protein genes [33]. In poribacterial BMC group B we found genes for riboflavin (vitamin B2) biosynthesis, which might indicate a riboflavin dependent process occurring in poribacterial BMCs. Riboflavin is a major cofactor in many processes of the energy metabolism. To our knowledge riboflavin biosynthesis genes have so far not been described from other BMC shell gene clusters. Further investigations will reveal, whether there are indeed BMCs with riboflavin dependent reactions. Furthermore, poribacterial BMC gene clusters show similar regulatory systems to previously described clusters. A recent study by Jorda et al. [36] identified clusters of BMCs shared between different organisms by comparing similarities of genes in the genomic neighborhoods of BMC shell proteins. They identified two BMC clusters that are characterized by a two-component regulatory system with a signal transduction histidine kinase and response regulator receiver [36]. Poribacterial BMC clusters appear to be similarly regulated, since we also detected genes of a two-component regulatory system in three out of four described poribacterial BMC groups (see Fig. 3, text S1, and tables S2–S5). However, none of the functional genes on poribacterial BMC clusters showed similarities to those on the clusters described by Jorda et al. [36] and therefore the true functions of poribacterial BMCs remain to be investigated.

It is suspected that novel BMC functions will be revealed in the future [36] especially from genomes with a more scattered operon structure [33]. This might also be the case for *Poribacteria* where the identified genomic regions with BMC shell protein genes (group A-C) appear scattered across the genome. For example, the different BMC shell protein genes (with pfam00936 and pfam03319) are generally in different genomic regions on poribacterial genomes and not encoded together within one region, as it is the case for many so far functionally characterized BMC types [33]. Functional components of poribacterial BMCs might therefore also be encoded on different genomic regions. Alternatively, the existence of only one pfam00936 domain and the occurrence of transposase genes in BMC gene clusters B and D (see text S1) might indicate lack of function [33]. Future efforts are needed to resolve this issue for *Poribacteria*.

### Eukaryote-like Repeat Proteins

**Figure 4:**
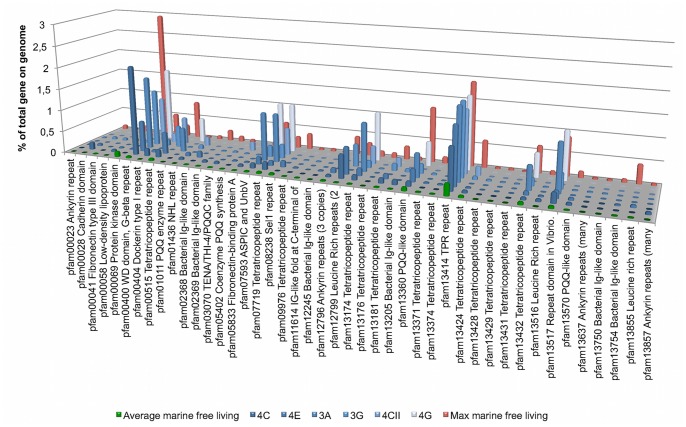
Bar plot showing frequency of eukaryote-like pfam domains found on poribacterial SAGs in comparison to the average and maximum frequency on all finished genomes of marine free-living bacteria available in IMG in July 2013.

Eukaryote-like repeat domain containing proteins have received much recent attention in sponge microbiology and their involvement in mediating host-microbe interactions has been postulated. Especially ankyrin (ANK) and tetratricopeptide repeats (TPR) have been in focus of such investigations [37]–[39]. To examine the role of these domains on poribacterial SAGs we searched for proteins with pfam hits to repeat and eukaryote-like domains in the IMG/MER database and also compared these to all finished genomes of free-living marine bacteria available in the IMG database in July 2013 (n = 98). We detected 41 such domains on poribacterial SAGs. The majority of these showed a higher domain frequency per total genes on at least one poribacterial SAG when compared to the average frequency of this domain on genomes of free-living marine bacteria (Fig. 4, Table S7). For 14 pfam domains the frequency on poribacterial genomes was even higher than the maximum frequency of this domain on the genome of any free-living marine bacterium. Many domains occurred simultaneously on the same genes with a total of 668 domains in all poribacterial SAGs on 490 encoded proteins (3A: 15 domains on 11 genes, 3G: 335 domains on 240 genes, 4C: 95 domain on 75 genes, 4CII: 24 domains on 16 genes, 4E: 181 domains on 135 genes, and 4G: 17 domains on 8 genes).

Among the most abundant domains were TPRs with pfams 013414, 00515, 07719, 13432, 13174, and 13181, which were also represented by eight other pfams (13424, 13374, 13371, 09976, 13431, 13429, 13428, and 13176) but in lower abundances. We were also able to find Sel1 repeat like proteins domains encoded on poribacterial SAGs 3G and 4E (0.02 and 0.15% of total genes, respectively) which have a similar structure to TPRs [40]. In total TPRs represented the highest frequency of repeat domains on poribacterial SAGs. Furthermore WD40 domains (pfam00400) were highly abundant on poribacterial SAGs, as well as two-copy leucin rich repeats (LRR) (pfam 12799), and the VCBS domain (pfam 13517) which is a domain found in high numbers in the genera *Vibrio*, *Colwellia*, *Bradyrhizobium* and *Shewanella*. Pfam domain 07593- ASPIC and UNbV was also present on several poribacterial SAGs in multiple copies. ANK repeat domains were detected (pfam 12796, 13637, 13857, and 00023) in lower numbers on a total of 14 genes on SAGs 3G, 4C and 4E (Table S7). The frequency of genes with pfam domains representing ankyrin repeats was often higher than average compared to the genomes of free-living marine bacteria (Table S7).

The occurrence of low-density lipoprotein (LDL) receptor repeat class B domains (pfam00058) on poribacterial genomes seemed noteworthy. We found these domains on one gene in each SAG 4C and 4E as well as on five genes in SAG 3G. Outside of *Poribacteria* this domain has only been found in proteins of 14 bacterial genomes but not in archaeal genomes publically available at the IMG/MER database in July 2013. Most of these bacterial hits however do not show the tandem repeats that are characteristic for this domain in eukaryotes. Such tandem repeats were only detected in the poribacterial proteins and proteins of four other bacterial genomes. Amongst these were free-living marine cyanobacteria (*Cyanothece* species, *Pleurocapsa* sp. PCC 7327), the marine deep sea piezophile *Mortiella* sp. PE36, and the strictly anaerobic bacterium *Paludibacter propionicigenes* WB4, DSM 17365. The LDL receptor is best described in mammals where they transport ligands into the cell for degradation by lysosomes and plays a role in cholesterol homeostasis [41]. The LDL repeat domain class B is part of the region of the LDL receptor which is responsible for ligand release and receptor recycling [42]. Virtually nothing is known about such domains in bacteria and it remains to be investigated whether there is a real connection to eukaryotic domains.

Although the limited data did not allow for any functional assignments of the LDL receptor genes, a role on the cell surface seems very likely in *Poribacteria* since all of the discovered genes with these domains had predicted transmembrane helices (TMHs) (∼86%) with the majority of the protein located outside of the cell or signal peptides (SPs) (∼14%). TMHs and SPs were also frequently predicted on genes representing other eukaryote-like proteins of *Poribacteria* (Table S8 and S9). High abundances (≥50% of genes with this pfam) of either TMHs or SPs were found on genes also encoding for bacterial Ig like domain protein genes, PQQ enzyme repeat containing genes, fibronectin type III domain and cadherin domain genes. Also genes with some of the pfams domains representing LRR and TPRs showed strong representation of TMH and SPs. Additionally, many poribacterial eukaryote-like domain genes (especially WD40 repeats) encoded for a domain potentially belonging to the Por secretion system C-terminal sorting domain family (TIGR04183) (Table S9), which is characteristic of proteins with outer membrane locations [43]–[45]. Since structural genes of the Por secretion system were not found on poribacterial genomes a potential secretion pathway for gene products with this domain remains to be revealed.

Our findings support previous reports of repeat and eukaryote-like domains being highly abundant in symbionts of marine sponges. The identification of proteins with these domains from the microbial communities of the sponge *Cymbastella concentrica* by ways of metaproteogenomics [46] might point towards an active functional role of these proteins. ANK domain proteins of sponge symbionts have been suspected to be involved in preventing phagocytosis by the sponge host as in analogy to similar functions of ANK domain proteins in bacterial pathogens *Legoniella pneumophila* or *Coxiella burnetti* [39], [47]. Indeed, in a recent paper Nguyen et al. [48] were able to show that ANK proteins from a marine sponge symbiont that were expressed in *E*.*coli* prevent phagocytosis of the bacterial cells by amoeba. The authors suggested this to be a function of sponge symbionts to avoid digestion by their host [48]. Thus, poribacterial ANK proteins may also facilitate similar functions.

LRRs have been found in proteins of pathogenic bacteria such as *Yersinia* species where LRRs are part of important virulence factors [49] or *Listeria monocytogenes* which encodes for LRR containing protein InlB that aids in host cell invasion [50]. Also TPRs were shown to be involved in different functions of pathogenesis [51] and fibronectin domains were shown to play a role in host-pathogen interactions as well, although in this case bacterial proteins bind to the fibronectin domains of the host protein [52], [53]. It would be interesting to explore whether bacterial fibronectin domains might be used in a similar way. Furthermore, fibronectin III domains have been found in polysaccharide degrading extracellular enzymes of *Clostridium thermocellum* [54]. Hentschel et al. [47] speculated that such functions in sponge symbionts could be connected to interactions with molecules of the sponge host extracellular matrix and our recent investigations of poribacterial carbohydrate degradation potential [14] support this hypothesis. However, at the current stage, we are just beginning to decipher the real functions of eukaryote-like proteins in *Poribacteria*. As many of these proteins may not be located outside of the poribacterial cell, as indicated by the large amount of proteins detected without TMHs or SPs (Table S9), it appears likely that at least some may mediate intracellular protein-protein interactions.

### High Abundance of phyH -domain Containing Proteins

Among poribacterial genomes we found a remarkably high occurrence of genes encoding for proteins with pfam domain pfam05721-phyH (Table S10). This pfam describes a protein family containing eukaryotic phytanoyl-CoA dioxygenase proteins, ectoine hydroxylases from eukaryotes and prokaryotes, and several bacterial deoxygenases of mostly unknown function (http://pfam.sanger.ac.uk/family/PF05721). These proteins are Fe(II) and 2-oxoglutarate dependent oxygenases that catalyze a wide range of oxidative reactions Among bacterial phyH genes are some potentially involved in quorum sensing [55], [56], synthesis of the compatible solute 5-hydroxyectoine [57], and utilization of phosphorous sources [58], [59]. We screened for this domain in all genomes publically available in the IMG/MER database in July 2013. All poribacterial genomes showed a frequency of more than 1.9% genes with this domain per total number of genes (Table S10). All other genomes available in the database at the time (independent of its domain Bacteria, Archaea, or Eukaryota) showed a frequency of less than 0.049% of genes with this domain per total genes. This large abundance of genes belonging to the same pfam family might indicate an importance of the related functions for *Poribacteria*.

A clustering analysis of poribacterial sequences showed that there was large diversity amongst poribacterial phyH family genes with 305 sequences clustering in 193 clusters with 60% aa id threshold (Table S11). For the majority of poribacterial genes with this domain a reliable functional annotation could not be made. Best homologies were usually between genes of poribacterial SAGs, despite the high diversity indicated by the clustering analysis. Some of the poribacterial phyH family genes also showed homology to another uncharacterized deoxygenase encoded on the first genome fragment sequence from a poribacterial metagenome clone 64K2 [60]. This might indicate *Poribacteria-*specific functions within the phyH family.

Although the majority of poribacterial phyH genes remained without further functional characterization, we were still able to make functional predictions in some cases. Poribacterial SAGs 3G and 4E encoded for phyH genes (OID 2265144857 and 2265139858, respectively) with homologies (40% aa id each) to a 2-aminoethylphosphonate (2-AEPn) utilization gene (phnY) for which function was experimentally proven [59]. These poribacterial genomes also encoded directly upstream of this gene for a protein of the HD phosphohydrolase family (phnZ) (OID 2265144856 in 3G and 2265139857 in 4E), which is the only other gene involved in this 2-AEPn utilization pathway [59]. Both poribacterial genomes further encoded for another predicted phosphohydrolase downstream of the previously described genes with as of yet unknown function in this pathway. 2-AEPn is assumed to be one of the biggest sources of dissolved organic phosphorous in the oceans [61], [62] and represents an alternative phosphorous source to the often limited dissolved inorganic phosphorous. The use of dissolved organic phosphorous i.e. phosphonates by many marine bacteria has been described before [63], [64]. Phosphonates such as 2-AEPn are found largely in phospholipids of marine invertebrates including sponges and are also produced by some marine bacteria [65]–[68]. Therefore organic phosphorous sources should be largely available in the sponge mesohyl and the ability to utilize 2-AEPn as a phosphorous source might therefore be a competitive advantage. The presence of both genes identified as essential for 2-AEPn utilization [59] indicated the presence of this pathway also in *Poribacteria* and elucidated one possible function of phyH superfamily genes in this candidate phylum.

## Conclusion

Our study demonstrates the power of single-cell genomics to reveal novel features of the candidate phylum *Poribacteria* which are almost exclusively found in association with marine sponges. Here we show by use of phylogenetic and phylogenomic analyses that *Poribacteria* are not members of the PVC superphylum, but rather form a distinct monophyletic phylum in close proximity. We provide genomic evidence for bacterial microcompartments in *Poribacteria* that show no similarity to any previously described BMCs. Further novel functions might be hidden in the various eukaryote-like protein domains, which may be involved in mediating host-microbe interactions within the sponge holobiont. The high abundance of diverse phyH-domain containing proteins points to important and potentially specific functions in *Poribacteria*. Most of these functions remain to be revealed in future studies but some show the genomic potential for organic phosphorous utilization. Our analyses show how genome sequences can help to revisit past hypotheses and at the same time open the way for new investigations by revealing novel functional features. Challenges for the future will be to experimentally demonstrate function and to ultimately understand the implications for symbiosis.

## Supporting Information

Table S1 **83 marker genes used for phylogenetic analysis.**
(PDF)

Table S2 **BMC group A genes with annotation.**
(PDF)

Table S3 **BMC group B genes with annotation.**
(PDF)

Table S4 **BMC group C genes with annotations.**
(PDF)

Table S5 **BMC group D genes with annotation.**
(PDF)

Table S6 **BMC shell protein pfam domain distribution on all genomes with either domain in IMG in July 2013.**
(PDF)

Table S7 **Overview of gene copy numbers (no) and percentage of genes per total genes on genome (%) of repeat proteins and eukaryote like protein domain genes on poribacterial SAGs and the maximum and average number of gene copies found on X finished genomes of marine free-living bacteria (n = 101).**
(PDF)

Table S8 **List of all repeat and eukaryote like domain protein encoding genes on poribacterial SAGs.** Information is shown as available in IMG/MER system. THM: number of predicted transmembrane helicies. SP: signal peptide predicted yes (Y) or no (N).
(PDF)

Table S9 **List of total repeat and eukaryote like protein domain encoding genes of poribacterial SAGs showing the number of genes (# genes), the number of genes with transmemebrane helicies (# TMH), percentage of genes with transmembrane helicies (% TMH), number of genes with signal peptide (# SP), and percentage of genes with signal peptide (% SP) for each domain.**
(PDF)

Table S10 **phyH domain distribution on publically available genomes.**
(PDF)

Table S11 **Poribacterial phyH gene clusters based on 60% amino acid identity.**
(PDF)

Text S1 **Genomic evidence for microcompartments in Poribacteria.** (extended description of genomic architecture)
(PDF)
