# Supplementary material for: The Candidate Phylum Poribacteria by Single-Cell Genomics: New Insights into Phylogeny, Cell-Compartmentation, Eukaryote-Like Repeat Proteins, and Other Genomic Features
Source: PLoS One. 2014 Jan 31;9(1):e87353. doi: 10.1371/journal.pone.0087353 (PMC3909097; doi:10.1371/journal.pone.0087353)
Supplement: Table S1 — 83 marker genes used for phylogenetic analysis. (PDF) [file pone.0087353.s001.pdf]

Table S1: 83 marker genes used for phylogenetic analysis.

HMM name	Gene name
pfam00162	PGK - Phosphoglycerate kinase
pfam00276	Ribosomal_L23 - Ribosomal protein L23
pfam00281	Ribosomal_L5 - Ribosomal protein L5
pfam00297	Ribosomal_L3 - Ribosomal protein L3
pfam00380	Ribosomal_S9 - Ribosomal protein S9/S16
pfam00410	Ribosomal_S8 - Ribosomal protein S8
pfam00411	Ribosomal_S11 - Ribosomal protein S11
pfam00416	Ribosomal_S13 - Ribosomal protein S13/S18
pfam00466	Ribosomal_L10 - Ribosomal protein L10
pfam00573	Ribosomal_L4 - Ribosomal protein L4/L1 family
pfam01795	Methyltransf_5 - MraW methylase family
TIGR00001	ribosomal protein L35
TIGR00002	ribosomal protein S16
TIGR00019	peptide chain release factor 1
TIGR00029	ribosomal protein S20
TIGR00043	probable rRNA maturation factor YbeY
TIGR00059	ribosomal protein L17
TIGR00060	ribosomal protein L18, bacterial type
TIGR00061	ribosomal protein L21
TIGR00064	signal recognition particle-docking protein FtsY
TIGR00082	ribosome-binding factor A
TIGR00086	SsrA-binding protein
TIGR00092	GTP-binding protein YchF
TIGR00115	trigger factor
TIGR00116	translation elongation factor Ts
TIGR00158	ribosomal protein L9
TIGR00165	ribosomal protein S18
TIGR00166	ribosomal protein S6
TIGR00168	translation initiation factor IF-3
TIGR00337	CTP synthase
TIGR00344	alanine--tRNA ligase
TIGR00362	chromosomal replication initiator protein DnaA
TIGR00388	glycyl-tRNA synthetase, tetrameric type, alpha subunit
TIGR00389	glycyl-tRNA synthetase, dimeric type
TIGR00409	prolyl-tRNA synthetase, family II
TIGR00459	aspartyl-tRNA synthetase, bacterial type
TIGR00460	methionyl-tRNA formyltransferase
TIGR00468	phenylalanyl-tRNA synthetase, alpha subunit
TIGR00471	phenylalanyl-tRNA synthetase, beta subunit
TIGR00472	phenylalanyl-tRNA synthetase, beta subunit, non-spirochete bacterial
TIGR00487	translation initiation factor IF-2
TIGR00496	ribosome recycling factor

TIGR00575	DNA ligase, NAD-dependent
TIGR00631	excinuclease ABC, B subunit
TIGR00663	DNA polymerase III, beta subunit
TIGR00810	protein translocase, SecG subunit
TIGR00855	ribosomal protein L7/L12
TIGR00922	transcription termination/antitermination factor NusG
TIGR00959	signal recognition particle protein
TIGR00963	preprotein translocase, SecA subunit
TIGR00964	preprotein translocase, SecE subunit, bacterial
TIGR00967	preprotein translocase, SecY subunit
TIGR00981	ribosomal protein S12, bacterial/organelle
TIGR01009	ribosomal protein S3, bacterial type
TIGR01011	ribosomal protein S2, bacterial type
TIGR01021	ribosomal protein S5, bacterial/organelle type
TIGR01024	ribosomal protein L19, bacterial type
TIGR01029	ribosomal protein S7, bacterial/organelle
TIGR01032	ribosomal protein L20
TIGR01044	ribosomal protein L22, bacterial type
TIGR01049	ribosomal protein S10, bacterial/organelle
TIGR01050	ribosomal protein S19, bacterial/organelle
TIGR01063	DNA gyrase, A subunit
TIGR01066	ribosomal protein L13, bacterial type
TIGR01067	ribosomal protein L14, bacterial/organelle
TIGR01071	ribosomal protein L15, bacterial/organelle
TIGR01079	ribosomal protein L24, bacterial/organelle
TIGR01164	ribosomal protein L16, bacterial/organelle
TIGR01169	ribosomal protein L1, bacterial/chloroplast
TIGR01171	ribosomal protein L2, bacterial/organellar
TIGR01391	DNA primase, catalytic core
TIGR01393	GTP-binding protein LepA
TIGR01632	50S ribosomal protein L11
TIGR01953	transcription termination factor NusA
TIGR02012	protein RecA
TIGR02013	DNA-directed RNA polymerase, beta subunit
TIGR02027	DNA-directed RNA polymerase, alpha subunit, bacterial and chloroplast-type
TIGR02386	DNA-directed RNA polymerase, beta' subunit, predominant form
TIGR02387	DNA-directed RNA polymerase, gamma subunit
TIGR02397	DNA polymerase III, subunit gamma and tau
TIGR02729	Obg family GTPase CgtA
TIGR03263	guanylate kinase
TIGR03594	ribosome-associated GTPase EngA
