# Supplementary material for: The Candidate Phylum Poribacteria by Single-Cell Genomics: New Insights into Phylogeny, Cell-Compartmentation, Eukaryote-Like Repeat Proteins, and Other Genomic Features
Source: PLoS One. 2014 Jan 31;9(1):e87353. doi: 10.1371/journal.pone.0087353 (PMC3909097; doi:10.1371/journal.pone.0087353)
Supplement: Table S2 — BMC group A genes with annotation. (PDF) [file pone.0087353.s002.pdf]

Table S2: BMC group A genes with annotation.

Genome	Gene ID	Locus Tag	Gene Product Name	COG	Pfam	Enzyme	KO
	2265138716	or2319	Carbon dioxide concentrating mechanism/carboxysome shell protein	COG4577 Carbon dioxide concentrating mechanism/carboxysome shell protein	pfam00936 BMC		
	2265138717	or2320	Propanediol utilization protein	COG4869 Propanediol utilization protein	pfam06130 PduL		KO:K15024 K15024 propanediol utilization protein
	2265138718	or2321	hypothetical protein				
	2265138719	or2322	hypothetical protein				
	2265138720	or2323	hypothetical protein				
	2265138721	or2324	hypothetical protein				
	2265138722	or2325	hypothetical protein				
	2265138723	or2326	Biopolymer transport proteins	COG0811 Biopolymer transport proteins	pfam13620 CarboxypepD_reg<<>>pfam01618 MotA_ExbB		
	2265138724	or2327	outer membrane transport energization protein ExbD (TC 2.C.1.1.1)	COG0848 Biopolymer transport protein	pfam02472 ExbD		KO:K03559 exbD biopolymer transport protein ExbD
4E	2265138725	or2328	Biopolymer transport protein	COG0848 Biopolymer transport protein	pfam02472 ExbD		KO:K03559 exbD biopolymer transport protein ExbD
	2265142941	POR3G_00336	Biopolymer transport protein	COG0848 Biopolymer transport protein	pfam02472 ExbD		KO:K03559 exbD biopolymer transport protein ExbD
3G	2265142942	POR3G_00337	Biopolymer transport	COG0848 Biopolymer transport	pfam02472 ExbD		KO:K03559 exbD biopolymer transport

			protein	protein			protein ExbD
	2265142943	POR3G_00338	Biopolymer transport proteins	COG0811 Biopolymer transport proteins	pfam13620 CarboxypepD_reg<<>>pfam01618 MotA_ExbB		
	2265142944	POR3G_00339	hypothetical protein				
	2265142945	POR3G_00340	Uncharacterized protein involved in exopolysaccharide biosynthesis	COG3206 Uncharacterized protein involved in exopolysaccharide biosynthesis	pfam13807 GNVR<<>>pfam02706 Wzz		
	2265142946	POR3G_00341	hypothetical protein				
	2265142947	POR3G_00342	hypothetical protein				
	2265142948	POR3G_00343	hypothetical protein	COG5422 RhoGEF, Guanine nucleotide exchange factor for Rho/Rac/Cdc42-like GTPases			
	2265142949	POR3G_00344	hypothetical protein				
	2265142950	POR3G_00345	Propanediol utilization protein	COG4869 Propanediol utilization protein	pfam06130 PduL		KO:K15024 K15024 propanediol utilization protein
	2265142951	POR3G_00346	Carbon dioxide concentrating mechanism/carboxysome shell protein	COG4577 Carbon dioxide concentrating mechanism/carboxysome shell protein	pfam00936 BMC		
	2265148187	POR4CII_00257	Carbon dioxide concentrating mechanism/carboxysome shell protein	COG4577 Carbon dioxide concentrating mechanism/carboxysome shell protein	pfam00936 BMC		
4CII	2265148188	POR4CII_00258	Propanediol utilization protein	COG4869 Propanediol utilization protein	pfam06130 PduL		KO:K15024 K15024 propanediol utilization protein

	2265148189	POR4CII_00259	hypothetical protein					
	2265148190	POR4CII_00260	hypothetical protein	COG5422 RhoGEF, Guanine nucleotide exchange factor for Rho/Rac/Cdc42-like GTPases				
	2265148191	POR4CII_00261	hypothetical protein					
	2265148192	POR4CII_00262	hypothetical protein					
	2265148193	POR4CII_00263	Uncharacterized protein involved in exopolysaccharide biosynthesis	COG3206 Uncharacterized protein involved in exopolysaccharide biosynthesis	pfam02706 Wzz<<>>pfam13807 GNVR			
	2265148194	POR4CII_00264	hypothetical protein					
	2265148195	POR4CII_00265	Biopolymer transport proteins	COG0811 Biopolymer transport proteins	pfam01618 MotA_ExbB<<>>pfam13620 CarboxypepD_reg			
	2265148196	POR4CII_00266	Biopolymer transport protein	COG0848 Biopolymer transport protein	pfam02472 ExbD		KO:K03559 exbD biopolymer transport protein ExbD	
	2265148197	POR4CII_00267	Biopolymer transport protein	COG0848 Biopolymer transport protein	pfam02472 ExbD		KO:K03559 exbD biopolymer transport protein ExbD	
