# Supplementary material for: The Candidate Phylum Poribacteria by Single-Cell Genomics: New Insights into Phylogeny, Cell-Compartmentation, Eukaryote-Like Repeat Proteins, and Other Genomic Features
Source: PLoS One. 2014 Jan 31;9(1):e87353. doi: 10.1371/journal.pone.0087353 (PMC3909097; doi:10.1371/journal.pone.0087353)
Supplement: Table S3 — BMC group B genes with annotation. (PDF) [file pone.0087353.s003.pdf]

Table S3: BMC group B genes with annotation.

Genome	Gene ID	Locus Tag	Gene Product Name	COG	Pfam	Tigrfam	Enzyme	KO
	2265140756	or1091	hypothetical protein					
	2265140757	or1092	hypothetical protein					
	2265140758	or1093	Trypsin-like serine proteases, typically periplasmic, contain C-terminal PDZ domain	COG0265Trypsin-like serine proteases, typically periplasmic, contain C-terminal PDZ domain				KO:K01362E3.4.21.-
	2265140759	or1094	Predicted O-methyltransferase	COG4122Predicted O-methyltransferase	pfam01596Methyltransf_3			
	2265140760	or1095	hypothetical protein					
	2265140761	or1096	Uncharacterized membrane protein (homolog of Drosophila rhomboid)	COG0705Uncharacterized membrane protein (homolog of Drosophila rhomboid)	pfam01694Rhomboid			
4E	2265140762	or1097	Carbon dioxide concentrating mechanism/c	COG4576Carbon dioxide concentrating mechanism/c	pfam03319EutN_CcmL			KO:K04028eutNethanolamine utilization protein EutN

			arboxysome shell protein	arboxysome shell protein				
	2265140763	or1098	Carbon dioxide concentrating mechanism/carboxysome shell protein	COG4576Carbon dioxide concentrating mechanism/carboxysome shell protein	pfam03319EutN_CcmL			KO:K04028eutNethanolamine utilization protein EutN
	2265140764	or1099	two component, sigma54 specific, transcriptional regulator, Fis family	COG2204Response regulator containing CheY-like receiver, AAA-type ATPase, and DNA-binding domains	pfam02954HTH_8<<>>pfam00158Sigma54_activat<<>>pfam00072Response_reg			KO:K07712glnG, ntrCtwo-component system, NtrC family, nitrogen regulation response regulator GlnG
	2265140765	or1100	Uncharacterized conserved protein containing a coiled-coil domain	COG5493Uncharacterized conserved protein containing a coiled-coil domain	pfam07788DUF1626<<>>pfam12644DUF3782			
	2265140766	or1101	Signal transduction histidine kinase, nitrogen specific	COG3852Signal transduction histidine kinase, nitrogen specific	pfam13492GAF_3<<>>pfam00512HisKA<<>>pfam02518HATPase_c			
	2265140767	or1102	imidazole glycerol phosphate synthase	COG0118Glutamine amidotransfer	pfam00117GATase	TIGR01855imidazole glycerol phosphate synthase,	EC:2.4.2.-Transferases. Glycosyltransferases. Pentosyltransferases.	KO:K02501hisHglutamine amidotransferase [EC:2.4.2.-]

			subunit hisH (EC 2.4.2.-)	ase		glutamine amidotransferase subunit		
	2265140768	or1103	hypothetical protein					
	2265140769	or1104	DNA modification methylase	COG0863DNA modification methylase	pfam01555N6_N4_Mtase		EC:2.1.1.72Site-specific DNA-methyltransferase (adenine-specific).	KO:K13581ccrMmodification methylase [EC:2.1.1.72]
	2265140770	or1105	6,7-dimethyl-8-ribityllumazine synthase (EC 2.5.1.78)	COG0054Riboflavin synthase beta-chain	pfam00885DMRL_synthase	TIGR001146,7-dimethyl-8-ribityllumazine synthase	EC:2.5.1.786,7-dimethyl-8-ribityllumazine synthase.	KO:K00794ribH, RIB46,7-dimethyl-8-ribityllumazine synthase [EC:2.5.1.78]
	2265140771	or1106	3,4-dihydroxy-2-butanone 4-phosphate synthase	COG01083,4-dihydroxy-2-butanone 4-phosphate synthase	pfam00925GTP_cyclohydro2<<>>pfam00926DHBP_synthase	TIGR00505GTP cyclohydrolase II<<>>TIGR005063,4-dihydroxy-2-butanone 4-phosphate synthase	EC:3.5.4.25GTP cyclohydrolase II.<<>>EC:4.1.99.123,4-dihydroxy-2-butanone-4-phosphate synthase.	KO:K14652ribBA3,4-dihydroxy 2-butanone 4-phosphate synthase / GTP cyclohydrolase II [EC:4.1.99.12 3.5.4.25]
	2265140772	or1107	riboflavin synthase alpha chain (EC 2.5.1.9)	COG0307Riboflavin synthase alpha chain	pfam00677Lum_binding	TIGR00187riboflavin synthase, alpha subunit	EC:2.5.1.9Riboflavin synthase.	KO:K00793ribE, RIB5riboflavin synthase [EC:2.5.1.9]
	2265140773	or1108	Leucine Rich Repeat./Cadherin domain.	COG4886Leucine-rich repeat (LRR) protein	pfam00028Cadherin<<>>pfam13855LRR_8			
3G	2265146660	POR3G_04171	Archaeal ATPase.					
	2265146661	POR3G_04172	Peroxiredoxin	COG1225Peroxiredoxin	pfam00578AhpC-TSA			

	2265146662	POR3G_04173	riboflavin biosynthesis protein RibD	COG0117Pyrimidine deaminase	pfam00383dCMP_cyt_deam_1<<>>pfam01872RibD_C	TIGR00227riboflavin-specific deaminase C-terminal domain<<>>TIGR00326riboflavin biosynthesis protein RibD	EC:1.1.1.1935-amino-6-(5-phosphoribosylamino)uracil reductase.<<>>EC:3.5.4.26Diaminohydroxyphosphoribosylaminopyrimidine deaminase.	KO:K11752ribDdiaminohydroxyphosphoribosylaminopyrimidine deaminase / 5-amino-6-(5-phosphoribosylamino)uracil reductase [EC:3.5.4.26 1.1.1.193]
	2265146663	POR3G_04174	riboflavin synthase, alpha subunit	COG0307Riboflavin synthase alpha chain	pfam00677Lum_binding	TIGR00187riboflavin synthase, alpha subunit	EC:2.5.1.9Riboflavin synthase.	KO:K00793ribE, RIB5riboflavin synthase [EC:2.5.1.9]
	2265146664	POR3G_04175	GTP cyclohydrolase II/3,4-dihydroxy-2-butanone 4-phosphate synthase	COG01083,4-dihydroxy-2-butanone 4-phosphate synthase	pfam00925GTP_cyclohydro2<<>>pfam00926DHBP_synthase	TIGR00505GTP cyclohydrolase II<<>>TIGR005063,4-dihydroxy-2-butanone 4-phosphate synthase	EC:4.1.99.123,4-dihydroxy-2-butanone-4-phosphate synthase.<<>>EC:3.5.4.25GTP cyclohydrolase II.	KO:K14652ribBA3,4-dihydroxy 2-butanone 4-phosphate synthase / GTP cyclohydrolase II [EC:4.1.99.12 3.5.4.25]
	2265146665	POR3G_04176	6,7-dimethyl-8-ribityllumazine synthase	COG0054Riboflavin synthase beta-chain	pfam00885DMRL_synthase	TIGR001146,7-dimethyl-8-ribityllumazine synthase	EC:2.5.1.786,7-dimethyl-8-ribityllumazine synthase.	KO:K00794ribH, RIB46,7-dimethyl-8-ribityllumazine synthase [EC:2.5.1.78]
	2265146666	POR3G_04177	imidazole glycerol phosphate synthase, glutamine amidotransferase subunit	COG0118Glutamine amidotransferase	pfam00117GATase	TIGR01855imidazole glycerol phosphate synthase, glutamine amidotransferase subunit	EC:2.4.2.-Transferases. Glycosyltransferases. Pentosyltransferases.	KO:K02501hisHglutamine amidotransferase [EC:2.4.2.-]
	2265146667	POR3G_04178	Signal transduction histidine kinase, nitrogen	COG3852Signal transduction histidine kinase,	pfam02518HATPase_c<<>>pfam13492GAF_3<<>>pfam00512HisKA			

			specific	nitrogen specific				
	2265146668	POR3G_04179	Response regulator containing CheY-like receiver, AAA-type ATPase, and DNA-binding domains	COG2204Response regulator containing CheY-like receiver, AAA-type ATPase, and DNA-binding domains	pfam00072Response_reg<<>>pfam00158Sigma54_activat<<>>pfam02954HTH_8			KO:K07712glnG, ntrCtwo-component system, NtrC family, nitrogen regulation response regulator GlnG
	2265146669	POR3G_04180	Carbon dioxide concentrating mechanism/carboxysome shell protein	COG4576Carbon dioxide concentrating mechanism/carboxysome shell protein	pfam03319EutN_CcmL			KO:K04028eutNethanolamine utilization protein EutN
	2265146670	POR3G_04181	Carbon dioxide concentrating mechanism/carboxysome shell protein	COG4576Carbon dioxide concentrating mechanism/carboxysome shell protein	pfam03319EutN_CcmL			KO:K04028eutNethanolamine utilization protein EutN
	2265146671	POR3G_04182	hypothetical protein					
	2265146672	POR3G_04183	Propeptide_C25.		pfam08126Propeptide_C25			
3A	2265147537	POR_0191.00000010	Response regulator containing CheY-like receiver, AAA-type ATPase, and DNA-	COG2204Response regulator containing CheY-like receiver, AAA-type ATPase, and DNA-	pfam02954HTH_8			

			binding domains	binding domains					
	2265147538	POR_0191.00000020	Carbon dioxide concentrating mechanism/carboxysome shell protein	COG4576Carbon dioxide concentrating mechanism/carboxysome shell protein	pfam03319EutN_CcmL				
