# Supplementary material for: The Candidate Phylum Poribacteria by Single-Cell Genomics: New Insights into Phylogeny, Cell-Compartmentation, Eukaryote-Like Repeat Proteins, and Other Genomic Features
Source: PLoS One. 2014 Jan 31;9(1):e87353. doi: 10.1371/journal.pone.0087353 (PMC3909097; doi:10.1371/journal.pone.0087353)
Supplement: Table S4 — BMC group C genes with annotations. (PDF) [file pone.0087353.s004.pdf]

Table S4: BMC group C genes with annotations.

Genome	Gene ID	Locus Tag	Gene Product Name	COG	Pfam	Tigrfam	Enzyme	KO
	2265140346	or0682	transposase, IS4 family	COG5433Transposase	pfam01609DDE_Tnp_1			
	2265140347	or0683	Coenzyme F390 synthetase	COG1541Coenzyme F390 synthetase	pfam00501AMP-binding		EC:6.2.1.30Phenylacetate--CoA ligase.	KO:K01912paaKphenylacetate-CoA ligase [EC:6.2.1.30]
	2265140348	or0684	hypothetical protein					
	2265140349	or0685	hypothetical protein	COG1196Chromosome segregation ATPases	pfam14346DUF4398			
	2265140350	or0686	hypothetical protein					
	2265140351	or0687	hypothetical protein					
	2265140352	or0688	Soluble lytic murein transglycosylase and related regulatory proteins (some contain LysM/invasin domains)	COG0741Soluble lytic murein transglycosylase and related regulatory proteins (some contain LysM/invasin domains)	pfam01464SLT			
4E	2265140353	or0689	Predicted dehydrogenases and related proteins	COG0673Predicted dehydrogenases and related proteins	pfam02894GFO_IDH_MocA_C<<>>pfam01408GFO_IDH_MocA			

	2265140354	or0690	Carbon dioxide concentrating mechanism/carboxysome shell protein	COG4576Carbon dioxide concentrating mechanism/carboxysome shell protein	pfam03319EutN_CcmL			KO:K04028eutNethanolamine utilization protein EutN
	2265140355	or0691	Signal transduction histidine kinase	COG0642Signal transduction histidine kinase	pfam13596PAS_10<<>>pfam02518HATPase_c<<>>pfam00512HisKA			
	2265140356	or0692	Response regulator containing CheY-like receiver, AAA-type ATPase, and DNA-binding domains	COG2204Response regulator containing CheY-like receiver, AAA-type ATPase, and DNA-binding domains	pfam02954HTH_8<<>>pfam00158Sigma54_activat<<>>pfam00072Response_reg			KO:K02481K02481two-component system, NtrC family, response regulator
	2265140357	or0693	5-formyltetrahydrofolate cyclo-ligase	COG02125-formyltetrahydrofolate cyclo-ligase	pfam018125-FTHF_cyc-lig	TIGR027275,10-methenyltetrahydrofolate synthetase	EC:6.3.3.25-formyltetrahydrofolate cyclo-ligase.	KO:K01934E6.3.3.25-formyltetrahydrofolate cyclo-ligase [EC:6.3.3.2]
	2265140358	or0694	S-adenosyl-methyltransferase MraW	COG0275Predicted S-adenosylmethionine-dependent methyltransferase involved in cell envelope biogenesis	pfam01795Methyltransf_5	TIGR0000616S rRNA (cytosine(1402)-N(4))-methyltransferase	EC:2.1.1.19916S rRNA (cytosine(1402)-N(4))-methyltransferase.	KO:K03438mraW, rsmH16S rRNA (cytosine1402-N4)-methyltransferase [EC:2.1.1.199]
	2265140359	or0695	Cell division protein	COG3116Cell division protein	pfam04999FtsL	TIGR02209cell division protein FtsL		

	2265140360	or0696	transposase, IS605 OrfB family, central region	COG0675Transposase and inactivated derivatives	pfam01385OrfB_IS605<<>>pfam12323HTH_OrfB_IS605<<>>pfam07282OrfB_Zn_ribbon	TIGR01766transposase, IS605 OrfB family, central region		KO:K07496K07496putative transposase
	2265145155	POR3G_02612	Predicted dehydrogenases and related proteins	COG0673Predicted dehydrogenases and related proteins	pfam01408GFO_IDH_MocA			
	2265145156	POR3G_02613	Uncharacterized protein conserved in cyanobacteria	COG4636Uncharacterized protein conserved in cyanobacteria	pfam05685Uma2			
	2265145157	POR3G_02614	hypothetical protein					
	2265145158	POR3G_02615	RNA polymerase sigma factor, sigma-70 family	COG1595DNA-directed RNA polymerase specialized sigma subunit, sigma24 homolog	pfam04542Sigma70_r2<<>>pfam08281Sigma70_r4_2	TIGR02937RNA polymerase sigma factor, sigma-70 family		KO:K03088SIG3.2, rpoERNA polymerase sigma-70 factor, ECF subfamily
	2265145159	POR3G_02616	hypothetical protein					
	2265145160	POR3G_02617	glycine oxidase ThiO	COG0665Glycine/D-amino acid oxidases (deaminating)	pfam01266DAO	TIGR02352glycine oxidase ThiO	EC:1.4.3.19Glycine oxidase.	KO:K03153thiOglycine oxidase [EC:1.4.3.19]
3G	2265145161	POR3G_02618	Carbon dioxide concentrating mechanism/carboxysome shell protein	COG4576Carbon dioxide concentrating mechanism/carboxysome shell protein	pfam03319EutN_CcmL			KO:K04028eutNethanolamine utilization protein EutN

	2265145162	POR3G_02619	5,10-methenyltetrahydrofolate synthetase	COG02125-formyltetrahydrofolate cyclo-ligase	pfam018125-FTHF_cyc-lig	TIGR027275,10-methenyltetrahydrofolate synthetase	EC:6.3.3.25-formyltetrahydrofolate cyclo-ligase.	KO:K01934E6.3.3.25-formyltetrahydrofolate cyclo-ligase [EC:6.3.3.2]
	2265145163	POR3G_02620	competence protein ComEA helix-hairpin-helix repeat region	COG1555DNA uptake protein and related DNA-binding proteins	pfam12836HHH_3	TIGR00426competence protein ComEA helix-hairpin-helix repeat region<<>>TIGR01259comEA protein		
	2265145164	POR3G_02621	Mn2+ and Fe2+ transporters of the NRAMP family	COG1914Mn2+ and Fe2+ transporters of the NRAMP family				
	2265145165	POR3G_02622	uroporphyrin-III C-methyltransferase	COG0007Uroporphyrinogen-III methylase	pfam02602HEM4<<>>pfam00590TP_methylase	TIGR01469uroporphyrin-III C-methyltransferase	EC:2.1.1.107Uroporphyrinogen-III C-methyltransferase.<<>>EC:4.2.1.75 Uroporphyrinogen-III synthase.	KO:K13542cobA-hemDuroporphyrinogen III methyltransferase / synthase [EC:2.1.1.107 4.2.1.75]
	2265145166	POR3G_02623	porphobilinogen deaminase	COG0181Porphobilinogen deaminase	pfam01379Porphobil_deam<<>>pfam03900Porphobil_deamC	TIGR00212porphobilinogen deaminase	EC:2.5.1.61Hydroxymethylbilane synthase.	KO:K01749hemC, HMBShydroxymethylbilane synthase [EC:2.5.1.61]
	2265145167	POR3G_02624	glutamyl-tRNA reductase	COG0373Glutamyl-tRNA reductase	pfam01488Shikimate_DH<<>>pfam00745GlutR_dimer<<>>pfam05201GlutR_N	TIGR01035glutamyl-tRNA reductase	EC:1.2.1.70Glutamyl-tRNA reductase.	KO:K02492hemAglutamyl-tRNA reductase [EC:1.2.1.70]
	2265145168	POR3G_02625	ABC-type uncharacterized transport	COG4137ABC-type uncharacterized transport system,	pfam01578Cytochrom_C_asm	TIGR03144cytochrome c-type biogenesis protein		

			system, permease component	permease component		CcsB			
	2265145169	POR3G_02626	siroheme synthase, N-terminal domain	COG1648Siroheme synthase (precorrin-2 oxidase/ferrochelatase domain)	pfam13241NAD_binding_7	TIGR01470siroheme synthase, N-terminal domain		
	2265145170	POR3G_02627	Geranylgeranyl pyrophosphate synthase	COG0142Geranylgeranyl pyrophosphate synthase	pfam00348polyprenyl_synt		EC:2.5.1.90All-trans-octaprenyl-diphosphate synthase.	KO:K02523ispBoctaprenyl-diphosphate synthase [EC:2.5.1.90]
	2265145171	POR3G_02628	glutamate-1-semialdehyde-2,1-aminomutase	COG0001Glutamate-1-semialdehyde aminotransferase	pfam00202Aminotran_3	TIGR00713glutamate-1-semialdehyde-2,1-aminomutase	EC:5.4.3.8Glutamate-1-semialdehyde 2,1-aminomutase.	KO:K01845hemLglutamate-1-semialdehyde 2,1-aminomutase [EC:5.4.3.8]
	2265145172	POR3G_02629	Delta-aminolevulinic acid dehydratase	COG0113Delta-aminolevulinic acid dehydratase	pfam00490ALAD		EC:4.2.1.24Porphobilinogen synthase.	KO:K01698hemB, ALADporphobilinogen synthase [EC:4.2.1.24]
	2265145173	POR3G_02630	hypothetical protein		pfam11306DUF3108			
	2265145174	POR3G_02631	hypothetical protein		pfam11306DUF3108			
	2265145175	POR3G_02632	Predicted dehydrogenases and related proteins	COG0673Predicted dehydrogenases and related proteins	pfam01408GFO_IDH_MocA			
