# Supplementary material for: The Candidate Phylum Poribacteria by Single-Cell Genomics: New Insights into Phylogeny, Cell-Compartmentation, Eukaryote-Like Repeat Proteins, and Other Genomic Features
Source: PLoS One. 2014 Jan 31;9(1):e87353. doi: 10.1371/journal.pone.0087353 (PMC3909097; doi:10.1371/journal.pone.0087353)
Supplement: Table S5 — BMC group D genes with annotation. (PDF) [file pone.0087353.s005.pdf]

## Table S5: BMC group D genes with annotation.

| Genome | Gene ID | Locus Tag | Gene Product Name | COG | Pfam | Tigrfam | Enzyme | KO |
|---|---|---|---|---|---|---|---|---|
| 4E | 2265138716 | or2319 | Carbon dioxide concentrating mechanism/carboxysome shell protein | COG4577 Carbon dioxide concentrating mechanism/carboxysome shell protein | pfam00936 BMC | | | |
| | 2265138717 | or2320 | Propanediol utilization protein | COG4869 Propanediol utilization protein | pfam06130 PduL | | KO:K15024 K15024 propanediol utilization protein | |
| | 2265138718 | or2321 | hypothetical protein | | | | | |
| | 2265138719 | or2322 | hypothetical protein | | | | | |
| | 2265138720 | or2323 | hypothetical protein | | | | | |
| | 2265138721 | or2324 | hypothetical protein | | | | | |
| | 2265138722 | or2325 | hypothetical protein | | | | | |
| | 2265138723 | or2326 | Biopolymer transport proteins | COG0811 Biopolymer transport proteins | pfam13620 CarboxypepD_reg<<>>pfam01618 MotA_ExbB | | | |
| | 2265138724 | or2327 | outer membrane transport energization protein | COG0848 Biopolymer transport protein | pfam02472 ExbD | | KO:K03559 exbD biopolymer transport protein | |

| | | | | | | | | |
|---|---|---|---|---|---|---|---|---|
| | | | ExbD (TC 2.C.1.1.1) | | | | ExbD | |
| | 2265138725 | or2328 | Biopolymer transport protein | COG0848 Biopolymer transport protein | pfam02472 ExbD | | KO:K03559 exbD biopolymer transport protein ExbD | |
| | 2265138726 | or2329 | Response regulator containing CheY-like receiver, AAA-type ATPase, and DNA-binding domains | COG2204Response regulator containing CheY-like receiver, AAA-type ATPase, and DNA-binding domains | pfam00072Response_reg<<>>pfam02954HTH_8<<>>pfam00158Sigma54_activat | | | KO:K02481K02481two-component system, NtrC family, response regulator |
| | 2265138727 | or2330 | Uncharacterized membrane protein (homolog of Drosophila rhomboid) | COG0705Uncharacterized membrane protein (homolog of Drosophila rhomboid) | pfam01694Rhomboid | | | |
| | 2265138728 | or2331 | Histidinol phosphatase and related hydrolases of the PHP family | COG1387Histidinol phosphatase and related hydrolases of the PHP family | pfam02811PHP | | | KO:K02347dpxDNA polymerase (family X) |
| | 2265138729 | or2332 | hypothetical protein | COG18074-amino-4-deoxy-L-arabinose transferase and related glycosyltransferases of PMT family | pfam13231PMT_2 | | | |
| | 2265138730 | or2333 | Methionyl-tRNA formyltransferase | COG0223Methionyl-tRNA formyltransferase | pfam00583Acetyltransf_1 | | | |
| | 2265138731 | or2334 | 3-hydroxyisobutyrate dehydrogenase and related beta- | COG20843-hydroxyisobutyrate dehydrogenase and related beta- | pfam03446NAD_binding_2 | | EC:1.1.1.313-hydroxyisobutyrate dehydrogenase. | KO:K00020E1.1.1.31, mmsB3-hydroxyisobutyrate dehydrogenase |

| | | | | | | | | |
|---|---|---|---|---|---|---|---|---|
| | | | hydroxyacid dehydrogenases | hydroxyacid dehydrogenases | | | | [EC:1.1.1.31] |
| | 2265138732 | or2335 | Nucleoside-diphosphate-sugar epimerases | COG0451Nucleoside-diphosphate-sugar epimerases | pfam01370Epimerase | | | |
| | 2265138733 | or2336 | conserved hypothetical protein | COG0392Predicted integral membrane protein | pfam03706UPF0104 | TIGR00374conserved hypothetical protein | | |
| | 2265138734 | or2337 | Predicted acetyltransferase | COG3981Predicted acetyltransferase | pfam13302Acetyltransf_3 | | | |
| | 2265138735 | or2338 | nicotinate-nucleotide pyrophosphorylase [carboxylating] (EC 2.4.2.19) | COG0157Nicotinate-nucleotide pyrophosphorylase | pfam01729QRPTase_C<<>>pfam02749QRPTase_N | TIGR00078nicotinate-nucleotide pyrophosphorylase | EC:2.4.2.19Nicotinate-nucleotide diphosphorylase (carboxylating). | KO:K00767nadC, QPRTnicotinate-nucleotide pyrophosphorylase (carboxylating) [EC:2.4.2.19] |
| | 2265138736 | or2339 | Uncharacterized protein conserved in cyanobacteria | COG4636Uncharacterized protein conserved in cyanobacteria | pfam05685Uma2 | | | |
| | 2265138737 | or2340 | hypothetical protein | | | | | |
| | 2265138738 | or2341 | Carbon dioxide concentrating mechanism/carboxysome shell protein | COG4577Carbon dioxide concentrating mechanism/carboxysome shell protein | pfam00936BMC | | | |
| | 2265138739 | or2342 | NAD-dependent aldehyde dehydrogenases | COG1012NAD-dependent aldehyde dehydrogenases | pfam00171Aldedh | | | KO:K13922pduPpropionaldehyde dehydrogenase |
| | 22651387 | or23 | Predicted N-acetylglucosaminyl | COG2956Predicted N-acetylglucosaminyl | pfam13414TPR_11<<>>pfam13174TPR_6 | | | |

| | | | | | | | | |
|---|---|---|---|---|---|---|---|---|
| | 40 | 43 | transferase | transferase | | | | |
| | 2265138741 | or2344 | Predicted pyridoxal phosphate-dependent enzyme apparently involved in regulation of cell wall biogenesis | COG0399Predicted pyridoxal phosphate-dependent enzyme apparently involved in regulation of cell wall biogenesis | pfam01041DegT_DnrJ_EryC1 | | | |
| | 2265138742 | or2345 | hypothetical protein | | | | | |
| | 2265138743 | or2346 | hypothetical protein | | | | | |
| | 2265138745 | or2347 | Transposase and inactivated derivatives | COG2963Transposase and inactivated derivatives | pfam01527HTH_Tnp_1 | | | |
| | 2265138746 | or2348 | hypothetical protein | | pfam13358DDE_3 | | | |
| | 2265138747 | or2349 | NADH:flavin oxidoreductases, Old Yellow Enzyme family | COG1902NADH:flavin oxidoreductases, Old Yellow Enzyme family | pfam07992Pyr_redox_2<<>>pfam00724Oxidored_FMN<<>>pfam00070Pyr_redox | | | |
