# Supplementary material for: The Candidate Phylum Poribacteria by Single-Cell Genomics: New Insights into Phylogeny, Cell-Compartmentation, Eukaryote-Like Repeat Proteins, and Other Genomic Features
Source: PLoS One. 2014 Jan 31;9(1):e87353. doi: 10.1371/journal.pone.0087353 (PMC3909097; doi:10.1371/journal.pone.0087353)
Supplement: Table S6 — BMC shell protein pfam domain distribution on all genomes with either domain in IMG in July 2013. (PDF) [file pone.0087353.s006.pdf]

Table S6: BMC shell protein pfam domain distribution on all genomes with either domain in IMG in July 2013.

Genome	pfam00936	pfam003319
**Candidatus Poribacteria WGA 3A**	**0**	**1**
**Candidatus Poribacteria WGA 3G**	**1**	**3**
**Candidatus Poribacteria WGA 4CII**	**1**	**0**
**Candidatus Poribacteria WGA 4E**	**2**	**3**
**Atribacteria bacterium JGI 0000079-F20 (TAbiofilm_001_232)**	**1**	**4**
**Atribacteria bacterium SCGC AAA252-M02 (Combined_Assembly_OP9_1__OP9)**	**2**	**4**
**Atribacteria bacterium SCGC AAA252-M02 (SAK_001_71)**	**0**	**1**
**Atribacteria bacterium SCGC AB-164-D21 (SAK_001_216)**	**0**	**1**
**Atribacteria bacterium SCGC AB-164-L03 (SAK_001_213)**	**0**	**3**
**Atribacteria bacterium SCGC AB-164-M20 (SAK_001_218)**	**2**	**4**
**Bacillus chagannorensis DSM 18086**	**2**	**3**
**Bacillus sp. M3-13**	**2**	**3**
**Blastopirellula marina SH 106T, DSM 3645**	**2**	**3**
**Chloroherpeton thalassium ATCC 35110**	**2**	**3**
**Gemmatimonadetes bacterium JGI 0000112-M07**	**7**	**8**
**Latescibacteria bacterium SCGC AAA257-K07 (Etoliko_001_145)**	**1**	**3**
**Melioribacter roseus P3M**	**6**	**7**
**Planctomyces brasiliensis IFAM 1448, DSM 5305**	**2**	**3**
**Planctomyces limnophilus Mu 290, DSM 3776**	**2**	**3**
**Planctomyces maris DSM 8797**	**2**	**3**
**Porphyra umbilicalis P.um.1-endophyte06694 (Porphyra_umbilicalis_P.um.1-endophyte06694)**	**2**	**3**
**Rhodopirellula baltica SH 1**	**2**	**3**
Acaryochloris marina MBIC11017	8	1
Acaryochloris sp CCMEE 5410	7	1
Acetobacterium dehalogenans DSM 11527	14	2
Acetobacterium woodii WB1, DSM 1030	8	1
Acetonema longum APO-1, DSM 6540	6	1
Acidimicrobium ferrooxidans ICP, DSM 10331	3	1
Acidithiobacillus caldus ATCC 51756	4	2

Acidithiobacillus caldus SM-1	3	2
Acidithiobacillus ferrivorans SS3	2	2
Acidithiobacillus ferrooxidans ATCC 23270	3	2
Acidithiobacillus ferrooxidans ATCC 53993	3	2
Acidithiobacillus sp. GGI-221	2	2
Aeromonas hydrophila hydrophila ATCC 7966	4	1
Alkaliphilus metalliredigens QYMF	15	3
Alkaliphilus oremlandii OhILAs	14	3
Allochromatium vinosum DSM 180	3	2
Aminomonas paucivorans GLU-3, DSM 12260	6	1
Anabaena circinalis AWQC131C (Submitted file with automatic translation by Kostas)	6	1
Anabaena circinalis AWQC310F (Submitted file with automatic translation by Kostas)	6	1
Anabaena cylindrica PCC 7122	7	1
Anabaena sp. PCC 7108	5	1
Anabaena variabilis ATCC 29413	6	1
Anaeroarcus burkinensis DSM 6283	7	1
Anaerobaculum hydrogeniformans ATCC BAA-1850	9	1
Anaerobaculum mobile NGA, DSM 13181	6	1
Anaerococcus hydrogenalis ACS-025-V-Sch4	7	1
Anaerococcus hydrogenalis DSM 7454	7	1
Anaerococcus tetradius ATCC 35098	6	1
Anaerococcus vaginalis ATCC 51170	6	1
Anaerolinea thermophila UNI-1	4	1
Anaeromusa acidaminophila DSM 3853	7	1
Anaerotruncus colihominis DSM 17241	3	3
Aneurinibacillus terranovensis DSM 18919	8	2
Arthrospira maxima CS-328	6	1
Arthrospira platensis C1 (Draft2 circular genome)	6	1
Arthrospira platensis NIES-39	6	1
Arthrospira platensis Paraca	6	1
Arthrospira sp. PCC 8005	6	1
Atopobium fossor DSM 15642	4	1
Atribacteria bacterium JGI 0000079-L04 (TAbiofilm_001_231)	2	2

Bacillus azotoformans LMG 9581	4	1
Bacillus fordii DSM 16014	3	1
Bacillus gelatini DSM 15865	4	1
Bacillus macauensis ZFHKF-1	4	1
Bacillus megaterium DSM 319	4	1
Bacillus megaterium QM B1551	4	1
Bacillus selenitireducens MLS10	5	1
Bacillus sp. 2_A_57_CT2	7	2
Bacillus sp. 37MA	4	1
Bacillus sp. B14905	4	1
Bacillus sp. UNC438CL73TsuS30	10	6
Bacillus sp. URHB0009	4	1
Bilophila sp. 4_1_30	7	1
Bilophila wadsworthia 3_1_6	6	1
Blautia producta ATCC 27340	3	1
Blautia producta DSM 2950	3	1
Blautia wexlerae AGR2146	8	1
Blautia wexlerae DSM 19850	10	1
Brachyspira innocens ATCC 29796	6	1
Brachyspira intermedia PWS/A	7	1
Bradyrhizobium sp. BTAi1	2	2
Bradyrhizobium sp. EC3.3	3	1
Bradyrhizobium sp. ORS278	2	2
Bradyrhizobium sp. STM 3809	2	2
Brevibacillus agri BAB-2500	8	2
Brevibacillus brevis NBRC 100599	4	1
Brevibacillus laterosporus DSM 25	9	2
Brevibacillus laterosporus NRS 682, LMG 15441	9	2
Brevibacillus sp. BC25	4	1
Brevibacillus sp. CF112	8	2
Budvicia aquatica DSM 5075	6	1
Caldalkalibacillus thermarum TA2.A1	3	1
Caldanaerobius polysaccharolyticum KMTHCJ, DSM 13641	5	1

Caldithrix abyssi LF13, DSM 13497	4	4
Caloramator australicus RC3	3	1
Calothrix desertica PCC 7102	7	1
Calothrix sp. PCC 6303	6	1
Calothrix sp. PCC 7103	7	1
Calothrix sp. PCC 7507	6	1
Candidatus Solibacter usitatus Ellin6076	6	3
Carboxydothermus ferrireducens DSM 11255	10	2
Carboxydothermus hydrogenoformans Z-2901, DSM 6008	4	1
Cellulosilyticum lentocellum RHM5, DSM 5427	5	1
Chamaesiphon minutus PCC 6605	6	1
Chroococcidiopsis sp. PCC 6712	7	1
Chroococcidiopsis thermalis PCC 7203	7	1
Citrobacter freundii 4_7_47CFAA	10	2
Citrobacter freundii ATCC 8090	10	2
Citrobacter koseri ATCC BAA-895	10	2
Citrobacter rodentium ICC168	10	2
Citrobacter sp. 30_2	10	2
Citrobacter sp. A1	10	2
Citrobacter sp. L17	10	2
Citrobacter youngae ATCC 29220	10	2
Cloacibacillus evryensis 158, DSM 19522	6	1
Clostridiales sp. 1_7_47FAA	6	1
Clostridium acidurici 9a, DSM 604	5	1
Clostridium asparagiforme DSM 15981	15	3
Clostridium beijerinckii G117	6	1
Clostridium beijerinckii NCIMB 8052	6	1
Clostridium bolteae ATCC BAA-613	7	1
Clostridium botulinum B Eklund 17B	12	2
Clostridium botulinum Ba4 657	5	1
Clostridium botulinum Bf	5	1
Clostridium botulinum BKT015925	4	1
Clostridium botulinum BoNT/A1 Hall	6	1

Clostridium botulinum BoNT/A1, ATCC 19397	6	1
Clostridium botulinum BoNT/A2 Kyoto-F	6	1
Clostridium botulinum BoNT/A3 Loch Maree	7	1
Clostridium botulinum BoNT/B1 Okra	7	1
Clostridium botulinum CFSAN001627	7	1
Clostridium botulinum CFSAN001628	5	1
Clostridium botulinum D 1873	4	1
Clostridium botulinum E1 BoNT E Beluga	12	2
Clostridium botulinum E3 Alaska E43	12	2
Clostridium botulinum F 230613	6	1
Clostridium botulinum F Langeland	7	1
Clostridium botulinum H04402 065	6	1
Clostridium botulinum NCTC 2916	5	1
Clostridium botulinum type A - Hall	6	1
Clostridium cadaveris AGR2141	14	3
Clostridium carboxidivorans P7, DSM 15243	16	3
Clostridium carboxidivorans P7, DSM 15243	16	3
Clostridium celatum DSM 1785	6	1
Clostridium cf. saccharolyticum K10	6	1
Clostridium citroniae WAL-17108	7	1
Clostridium clostridioforme 2_1_49FAA	6	1
Clostridium clostridioforme AGR2157	13	2
Clostridium difficile 630 (epidemic type X)	5	1
Clostridium difficile ATCC 9689	5	1
Clostridium difficile BI9	5	1
Clostridium difficile CD196	5	1
Clostridium difficile CIP 107932	5	1
Clostridium difficile DSM 1296	5	1
Clostridium difficile NAP07	5	1
Clostridium difficile NAP08	5	1
Clostridium difficile QCD-32g58	5	1
Clostridium difficile QCD-37x79, NAP1a/001	5	1
Clostridium difficile QCD-63q42	5	1

Clostridium difficile QCD-66c26	5	1
Clostridium difficile QCD-76w55, NAP1	5	1
Clostridium difficile QCD-97b34, NAP1b/006	5	1
Clostridium difficile R20291	5	1
Clostridium difficile VPI 10463, ATCC 43255	5	1
Clostridium glycolicum DSM 1288	5	1
Clostridium halophilum ATCC 49637	28	6
Clostridium hathewayi DSM 13479	7	1
Clostridium hathewayi WAL-18680	2	1
Clostridium hiranonis TO-931 DSM 13275	3	1
Clostridium hylemonae DSM 15053	3	1
Clostridium indolis DSM 755	11	2
Clostridium kluyveri DSM 555	10	1
Clostridium kluyveri NBRC 12016	10	1
Clostridium ljungdahlii PETC, DSM 13528	14	3
Clostridium methoxybenzovorans SR3, DSM 12182	11	2
Clostridium methylpentosum R2, DSM 5476	7	1
Clostridium novyi NT	3	1
Clostridium paraputrificum AGR2156	6	1
Clostridium perfringens 13	5	1
Clostridium perfringens ATCC 13124	5	1
Clostridium perfringens CPE F4969	5	1
Clostridium perfringens NCTC 8239	5	1
Clostridium perfringens type B - ATCC 3626	5	1
Clostridium perfringens type C - JGS1495	5	1
Clostridium perfringens type D - JGS1721	5	1
Clostridium perfringens type E - JGS1987	5	1
Clostridium phytofermentans ISDg	17	3
Clostridium ragsdalei P11	10	2
Clostridium saccharolyticum WM1, DSM 2544	16	3
Clostridium sp. 7_3_54FAA	5	1
Clostridium sp. ASBs410	16	3
Clostridium sp. D5	10	1

Clostridium sp. MSTE9	6	1
Clostridium sporogenes ATCC 15579	7	1
Clostridium sporosphaeroides DSM 1294	6	1
Clostridium sticklandii DSM 519	9	2
Clostridium symbiosum WAL-14163	5	1
Clostridium symbiosum WAL-14673	5	1
Collinsella tanakaei YIT 12063	7	1
Coprococcus catus GD/7	4	1
Crinalium epipsammum PCC 9333	5	1
Crocosphaera watsonii WH 8501	6	1
Cyanobacterium aponinum PCC 10605	6	1
cyanobacterium PCC 7702	6	1
Cyanobacterium sp. ESFC-1	6	1
Cyanobacterium stanieri PCC 7202	7	1
Cyanobium gracile PCC 6307	3	2
Cyanobium sp. PCC 7001	3	2
Cyanothece sp. BH63E, ATCC 51472	6	1
Cyanothece sp. BH68, ATCC 51142	6	1
Cyanothece sp. CCY 0110	6	1
Cyanothece sp. PCC 7424	7	1
Cyanothece sp. PCC 7425	7	1
Cyanothece sp. PCC 7822	7	1
Cyanothece sp. PCC 8801	6	1
Cyanothece sp. PCC 8802	6	1
Cylindrospermopsis raciborskii CS-505	6	1
Cylindrospermum stagnale PCC 7417	6	1
Dactylococcopsis salina PCC 8305	6	1
Desulfatibacillum aliphaticivorans DSM 15576	5	1
Desulfatibacillum alkenivorans AK-01 (Desulfatibacillum alkenivorans AK-01)	5	1
Desulfitobacterium dehalogenans JW/IU-DC1, ATCC 51507	10	2
Desulfitobacterium hafniense DCB-2	11	3
Desulfitobacterium hafniense DP7 (draft 151 contigs)	11	3
Desulfitobacterium hafniense PCP-1	12	3

Desulfitobacterium hafniense TCE-1	12	3
Desulfitobacterium hafniense TCP-A	8	2
Desulfitobacterium hafniense Y51	11	3
Desulfitobacterium sp. LBE	12	3
Desulfitobacterium sp. PCE1, DSM 10344	10	2
Desulfobulbus japonicus DSM 18378	6	1
Desulfospira joergensenii DSM 10085	3	1
Desulfosporosinus acidiphilus SJ4, DSM 22704	7	1
Desulfosporosinus meridiei S10, DSM 13257	15	3
Desulfosporosinus orientis Singapore I, DSM 765	16	3
Desulfosporosinus sp. OT	17	3
Desulfosporosinus youngiae JW/YJL-B18, DSM 17734	15	3
Desulfotalea psychrophila LSv54	11	2
Desulfotomaculum guttoideum DSM 4024	11	2
Desulfotomaculum reducens MI-1	13	2
Desulfotomaculum ruminis DL, DSM 2154	7	1
Desulfovibrio acrylicus DSM 10141	11	2
Desulfovibrio alaskensis DSM 16109	7	1
Desulfovibrio alaskensis G20	7	1
Desulfovibrio cuneatus DSM 11391	7	1
Desulfovibrio desulfuricans aestuarii ATCC 29578	11	2
Desulfovibrio desulfuricans desulfuricans ATCC 27774	7	1
Desulfovibrio desulfuricans desulfuricans DSM 642	7	1
Desulfovibrio hydrothermalis DSM 14728	7	1
Desulfovibrio salexigens DSM 2638	7	1
Desulfovibrio zosterae DSM 11974	7	1
Desulfurispora thermophila DSM 16022	4	1
Dethiosulfovibrio peptidovorans SEBR 4207, DSM 11002	7	1
Ectothiorhodospira haloalkaliphila Imhoff 51/7, ATCC 51935	2	2
Endozoicomonas elysicola DSM 22380	5	1
Enterobacter aerogenes FGI35	4	1
Enterobacter aerogenes KCTC 2190	4	1
Enterobacter cloacae SCF1	10	2

Enterobacteriaceae bacterium 9_2_54FAA	6	1
Enterococcus faecalis 599	4	1
Enterococcus faecalis 62	4	1
Enterococcus faecalis AR01/DG	4	1
Enterococcus faecalis ATCC 29200	4	1
Enterococcus faecalis ATCC 29212	4	1
Enterococcus faecalis ATCC 4200	4	1
Enterococcus faecalis CH188	4	1
Enterococcus faecalis D6	4	1
Enterococcus faecalis DAPTO 512	4	1
Enterococcus faecalis DAPTO 516	4	1
Enterococcus faecalis DS5, ATCC 14508	4	1
Enterococcus faecalis E1Sol	4	1
Enterococcus faecalis ERV103	4	1
Enterococcus faecalis ERV116	4	1
Enterococcus faecalis ERV129	4	1
Enterococcus faecalis ERV25	4	1
Enterococcus faecalis ERV31	4	1
Enterococcus faecalis ERV37	4	1
Enterococcus faecalis ERV41	4	1
Enterococcus faecalis ERV62	4	1
Enterococcus faecalis ERV63	4	1
Enterococcus faecalis ERV65	4	1
Enterococcus faecalis ERV68	4	1
Enterococcus faecalis ERV72	4	1
Enterococcus faecalis ERV73	4	1
Enterococcus faecalis ERV81	4	1
Enterococcus faecalis ERV85	4	1
Enterococcus faecalis ERV93	4	1
Enterococcus faecalis Fly1	4	1
Enterococcus faecalis HH22	4	1
Enterococcus faecalis HIP11704	4	1
Enterococcus faecalis JH1	4	1

Enterococcus faecalis Merz96	4	1
Enterococcus faecalis OG1RF, ATCC 47077	4	1
Enterococcus faecalis PC1.1	4	1
Enterococcus faecalis R712	4	1
Enterococcus faecalis S613	4	1
Enterococcus faecalis T1	4	1
Enterococcus faecalis T11 (Okinawa-C1)	4	1
Enterococcus faecalis T2, Sapporo-603	4	1
Enterococcus faecalis T3, Sapporo-109	4	1
Enterococcus faecalis T8	4	1
Enterococcus faecalis TUSoD Ef11	4	1
Enterococcus faecalis TX0102	4	1
Enterococcus faecalis TX0104	4	1
Enterococcus faecalis TX0109	4	1
Enterococcus faecalis TX0411	4	1
Enterococcus faecalis TX0470	4	1
Enterococcus faecalis TX0635	4	1
Enterococcus faecalis TX0855	4	1
Enterococcus faecalis TX0860	4	1
Enterococcus faecalis TX1322	4	1
Enterococcus faecalis TX2134	4	1
Enterococcus faecalis TX4248	4	1
Enterococcus faecalis V583	4	1
Enterococcus faecalis X98, ATCC 27276	4	1
Escherichia albertii TW07627	6	1
Escherichia coli 101-1	8	2
Escherichia coli 11-02030	4	1
Escherichia coli 11-02033-1	4	1
Escherichia coli 11-03943	4	1
Escherichia coli 1827-70	4	1
Escherichia coli 2362-75	10	2
Escherichia coli 55989	4	1
Escherichia coli 83972	4	1

Escherichia coli 99.0713	4	1
Escherichia coli A0 34/86	6	1
Escherichia coli ABU 83972	4	1
Escherichia coli AIEC UM146	8	2
Escherichia coli B REL606	4	1
Escherichia coli B088	4	1
Escherichia coli B185	4	1
Escherichia coli B354	10	2
Escherichia coli BL21 (DE3)	4	1
Escherichia coli BL21(DE3)	4	1
Escherichia coli BL21(DE3)	4	1
Escherichia coli BW2952	4	1
Escherichia coli C ATCC 8739	4	1
Escherichia coli clone D i14	4	1
Escherichia coli clone D i2	4	1
Escherichia coli DEC10A	4	1
Escherichia coli DEC10B	4	1
Escherichia coli DEC10C	4	1
Escherichia coli DEC10D	4	1
Escherichia coli DEC10E	4	1
Escherichia coli DEC10F	4	1
Escherichia coli DEC11A	4	1
Escherichia coli DEC11B	4	1
Escherichia coli DEC11C	4	1
Escherichia coli DEC11D	4	1
Escherichia coli DEC12A	4	1
Escherichia coli DEC12B	4	1
Escherichia coli DEC12C	4	1
Escherichia coli DEC12D	4	1
Escherichia coli DEC13A	4	1
Escherichia coli DEC13C	4	1
Escherichia coli DEC13D	4	1
Escherichia coli DEC14A	4	1

Escherichia coli DEC14D	4	1
Escherichia coli DEC15A	4	1
Escherichia coli DEC15B	4	1
Escherichia coli DEC15C	4	1
Escherichia coli DEC15D	4	1
Escherichia coli DEC15E	4	1
Escherichia coli DEC5B	4	1
Escherichia coli DEC8C	4	1
Escherichia coli DEC8D	4	1
Escherichia coli DEC9A	4	1
Escherichia coli DEC9B	4	1
Escherichia coli DEC9D	4	1
Escherichia coli DEC9E	4	1
Escherichia coli DH1 (ME8569)	4	1
Escherichia coli DH10B	4	1
Escherichia coli E22 (EPEC)	4	1
Escherichia coli Ec12-0465	4	1
Escherichia coli Ec12-0466	4	1
Escherichia coli EC1734	4	1
Escherichia coli EC1738	4	1
Escherichia coli EC4100B	10	2
Escherichia coli ETEC H10407	4	1
Escherichia coli FVEC1302	10	2
Escherichia coli FVEC1412	10	2
Escherichia coli H299	4	1
Escherichia coli H591	4	1
Escherichia coli H736	4	1
Escherichia coli IAI1	4	1
Escherichia coli IAI39	8	2
Escherichia coli J53	4	1
Escherichia coli J96	4	1
Escherichia coli K-12, MG1655	4	1
Escherichia coli K12 DH1, ATCC 33849	4	1

Escherichia coli K12- W3110	4	1
Escherichia coli KO11FL	4	1
Escherichia coli LF82	10	2
Escherichia coli M605	4	1
Escherichia coli M718	4	1
Escherichia coli MS 107-1	4	1
Escherichia coli MS 115-1	10	2
Escherichia coli MS 116-1	8	2
Escherichia coli MS 119-7	4	1
Escherichia coli MS 124-1	4	1
Escherichia coli MS 145-7	4	1
Escherichia coli MS 146-1	4	1
Escherichia coli MS 175-1	4	1
Escherichia coli MS 182-1	4	1
Escherichia coli MS 185-1	4	1
Escherichia coli MS 187-1	4	1
Escherichia coli MS 196-1	4	1
Escherichia coli MS 198-1	4	1
Escherichia coli MS 200-1	8	2
Escherichia coli MS 21-1	8	2
Escherichia coli MS 45-1	4	1
Escherichia coli MS 69-1	8	2
Escherichia coli MS 78-1	4	1
Escherichia coli MS 84-1	4	1
Escherichia coli NA114	4	1
Escherichia coli NC101	10	2
Escherichia coli O1:K1:H7 (APEC)	7	2
Escherichia coli O1:K1:H7 U5/41, DSM 30083	8	2
Escherichia coli O103:H2 12009	4	1
Escherichia coli O104:H4 04-8351	4	1
Escherichia coli O104:H4 09-7901	4	1
Escherichia coli O104:H4 11-3677	4	1
Escherichia coli O104:H4 2009EL-2050	4	1

Escherichia coli O104:H4 2009EL-2071	4	1
Escherichia coli O104:H4 2011C-3493	4	1
Escherichia coli O104:H4 C227-11	4	1
Escherichia coli O104:H4 Ec11-4986	4	1
Escherichia coli O104:H4 Ec11-4987	4	1
Escherichia coli O104:H4 Ec11-4988	4	1
Escherichia coli O104:H4 Ec11-5603	4	1
Escherichia coli O104:H4 Ec11-5604	4	1
Escherichia coli O104:H4 Ec11-9450	4	1
Escherichia coli O104:H4 Ec11-9941	4	1
Escherichia coli O111 CVM9455	4	1
Escherichia coli O111:H 11128	4	1
Escherichia coli O111:H11 CVM9553	4	1
Escherichia coli O111:H8 CVM9602	4	1
Escherichia coli O111:H8 CVM9634	4	1
Escherichia coli O111:H9 E110019 (EPEC)	8	1
Escherichia coli O111:NM B171 (EPEC2)	4	1
Escherichia coli O127:H6 E2348/69 (EPEC)	10	2
Escherichia coli O139:H28 E24377A (ETEC)	10	2
Escherichia coli O139:H28 F11 (ETEC)	8	2
Escherichia coli O144 53638	4	1
Escherichia coli O148:H28 B7A (CS6:LT+:ST+)(ETEC)	4	1
Escherichia coli O150:H5 SE15	4	1
Escherichia coli O157:H43 T22	5	1
Escherichia coli O157:H7 EC4024	4	1
Escherichia coli O157:H7 EC4042	4	1
Escherichia coli O157:H7 EC4045	4	1
Escherichia coli O157:H7 EC4076	4	1
Escherichia coli O157:H7 EC4113	4	1
Escherichia coli O157:H7 EC4115	4	1
Escherichia coli O157:H7 EC4196	4	1
Escherichia coli O157:H7 EC4206	4	1
Escherichia coli O157:H7 EC4401	4	1

Escherichia coli O157:H7 EC4486	4	1
Escherichia coli O157:H7 EC4501	4	1
Escherichia coli O157:H7 EC508	4	1
Escherichia coli O157:H7 EC869	4	1
Escherichia coli O157:H7 EDL933 (EHEC)	4	1
Escherichia coli O157:H7 FRIK2000	4	1
Escherichia coli O157:H7 FRIK966	4	1
Escherichia coli O157:H7 Sakai (EHEC)	4	1
Escherichia coli O157:H7 TW14359	4	1
Escherichia coli O157:H7 TW14588	4	1
Escherichia coli O17:K52:H18 UMN026	9	2
Escherichia coli O18:K1:H7 IHE3034	4	1
Escherichia coli O26:H11 11368	4	1
Escherichia coli O26:H11 CVM10021	4	1
Escherichia coli O26:H11 CVM10030	4	1
Escherichia coli O26:H11 CVM10224	4	1
Escherichia coli O26:H11 CVM9952	4	1
Escherichia coli O32:H37 P4	4	1
Escherichia coli O44:H18 042 (EAEC)	4	1
Escherichia coli O45:K1 S88	7	2
Escherichia coli O55:H7 CB9615	4	1
Escherichia coli O55:H7 RM12579	4	1
Escherichia coli O6:K15:H31 536 (UPEC)	8	2
Escherichia coli O6:K2:H1 CFT073 (UPEC)	8	2
Escherichia coli O7:K1 CE10	8	2
Escherichia coli O81 ED1a	8	2
Escherichia coli O83:H1 NRG 857C	10	2
Escherichia coli O9 HS	4	1
Escherichia coli P12b	4	1
Escherichia coli SCI-07	10	2
Escherichia coli SE11	4	1
Escherichia coli SECEC SMS-3-5	4	1
Escherichia coli TA143	10	2

Escherichia coli TA206	10	2
Escherichia coli TA271	10	2
Escherichia coli TA280	4	1
Escherichia coli TW10119	4	1
Escherichia coli TW10246	4	1
Escherichia coli TW11039	4	1
Escherichia coli UMNF18	4	1
Escherichia coli UMNK88	4	1
Escherichia coli UTI89 (UPEC)	8	2
Escherichia coli W	4	1
Escherichia coli W, ATCC 9637	4	1
Escherichia coli W, ATCC 9739	4	1
Escherichia coli Xuzhou21	4	1
Escherichia fergusonii UMN026, ATCC 35469	13	3
Escherichia hermannii NBRC 105704	4	1
Escherichia sp. 3_2_53FAA	6	2
Escherichia sp. 4_1_40B	4	1
Escherichia sp. TW09308	10	2
Eubacterium hallii DSM 3353	12	1
Eubacterium limosum KIST612	7	1
Eubacterium sp. ACC19a	5	1
Eubacterium sp. CM2	5	1
Eubacterium sp. CM5	5	1
Eubacterium yurii margaretiae ATCC 43715	6	1
Faecalibacterium cf. prausnitzii KLE1255	1	1
Fischerella sp. JSC-11	6	1
Fischerella sp. PCC 9339	6	1
Fischerella sp. PCC 9431	6	1
Fischerella sp. PCC 9605	6	1
Flavonifractor plautii ATCC 29863	16	3
Fusobacterium nucleatum nucleatum ATCC 23726	4	1
Fusobacterium nucleatum nucleatum ATCC 25586	4	1
Fusobacterium nucleatum polymorphum ATCC 10953	7	2

Fusobacterium sp. 1_1_41FAA	4	1
Fusobacterium sp. 11_3_2	7	2
Fusobacterium sp. 12_1B	4	1
Fusobacterium sp. 2_1_31	4	1
Fusobacterium sp. 21_1A	4	1
Fusobacterium sp. 3_1_27	4	1
Fusobacterium sp. 3_1_33	7	2
Fusobacterium sp. 3_1_36A2	4	1
Fusobacterium sp. 4_1_13	4	1
Fusobacterium sp. 7_1	7	2
Fusobacterium sp. D11	7	2
Fusobacterium ulcerans ATCC 49185	4	1
Fusobacterium varium ATCC 27725	9	2
Geitlerinema sp. PCC 7105	6	1
Geitlerinema sp. PCC 7407	6	1
Geminocystis herdmanii PCC 6308	6	1
Geobacillus sp. Y4.1MC1	4	1
Geobacillus thermoglucosidasius C56-YS93	4	1
Gloeobacter violaceus PCC 7421	4	1
Gloeocapsa sp. PCC 73106	7	1
Gloeocapsa sp. PCC 7428	5	1
Hafnia alvei ATCC 51873	6	1
Halanaerobium hydrogenoformans	9	1
Halanaerobium praevalens GSL, DSM 2228	7	1
Haliangium ochraceum SMP-2, DSM 14365	4	3
Halothece sp. PCC 7418	6	1
Halothiobacillus neapolitanus c2, ATCC 23641	4	2
Ilyobacter polytropus CuHBu1, DSM 2926	5	1
Isosphaera pallida IS1B, ATCC 43644	3	3
Kineosphaera limosa NBRC 100340	3	1
Klebsiella oxytoca E718	14	3
Klebsiella oxytoca KCTC 1686	14	3
Klebsiella oxytoca M5al	14	3

Klebsiella pneumoniae 342	14	3
Klebsiella pneumoniae KCTC 2242	10	2
Klebsiella pneumoniae NTUH-K2044	10	2
Klebsiella pneumoniae pneumoniae HS11286	10	2
Klebsiella pneumoniae pneumoniae KPNIH1	10	2
Klebsiella pneumoniae pneumoniae KPNIH10	10	2
Klebsiella pneumoniae pneumoniae KPNIH11	10	2
Klebsiella pneumoniae pneumoniae KPNIH12	10	2
Klebsiella pneumoniae pneumoniae KPNIH14	10	2
Klebsiella pneumoniae pneumoniae KPNIH2	10	2
Klebsiella pneumoniae pneumoniae KPNIH4	10	2
Klebsiella pneumoniae pneumoniae KPNIH5	10	2
Klebsiella pneumoniae pneumoniae KPNIH6	10	2
Klebsiella pneumoniae pneumoniae KPNIH7	10	2
Klebsiella pneumoniae pneumoniae KPNIH8	10	2
Klebsiella pneumoniae pneumoniae KPNIH9	10	2
Klebsiella pneumoniae pneumoniae MGH78578	10	2
Klebsiella pneumoniae rhinoscleromatis ATCC 13884	14	3
Klebsiella sp. 1_1_55	10	2
Klebsiella sp. 4_1_44FAA	10	2
Klebsiella sp. MS 92-3	14	3
Klebsiella variicola At-22	14	3
Lachnospiraceae bacterium 1_4_56FAA	8	1
Lachnospiraceae bacterium 2_1_58FAA	10	1
Lachnospiraceae bacterium 3_1_57FAA_CT1	6	1
Lachnospiraceae bacterium NK4A144	7	1
Lachnospiraceae bacterium sp. 7_1_58FAA	15	3
Lactobacillus brevis ATCC 367	5	2
Lactobacillus coryniformis coryniformis 34, KCTC 3167	6	1
Lactobacillus coryniformis torquens 30, KCTC 3535	6	1
Lactobacillus kisonensis F0435	5	1
Lactobacillus reuteri CF48-3A	5	1
Lactobacillus reuteri F275, JCM 1112	5	1

Lactobacillus reuteri F275, JCM 1112	5	1
Lactobacillus reuteri MM2-3	5	1
Lactobacillus reuteri MM4-1A	5	1
Lactobacillus reuteri SD2112, ATCC 55730	5	1
Lactobacillus rossiae DSM 15814	8	1
Lactobacillus versmoldensis KCTC 3814	6	1
Lamprocystis purpurea DSM 4197	2	2
Latescibacteria bacterium SCGC AAA252-B13 (SAK_001_32)	4	3
Leptolyngbya boryana PCC 6306	5	1
Leptolyngbya sp. PCC 6406	6	1
Leptolyngbya sp. PCC 7375	6	1
Leptolyngbya sp. PCC 7376	6	1
Leptotrichia buccalis C-1013-b, DSM 1135	6	1
Leptotrichia hofstadii DSM 21651	6	1
Leptotrichia hofstadii F0254	6	1
Leptotrichia trevisanii DSM 22070	5	1
Listeria innocua Clip11262	10	2
Listeria ivanovii ivanovii PAM 55	10	2
Listeria monocytogenes 07PF0776	10	2
Listeria monocytogenes 08-5578	10	2
Listeria monocytogenes 08-5923	10	2
Listeria monocytogenes 1/2a F6854	10	2
Listeria monocytogenes 10403S	10	2
Listeria monocytogenes 4a L99	10	2
Listeria monocytogenes 4b CLIP 80459	10	2
Listeria monocytogenes 4b F2365	10	2
Listeria monocytogenes 4b H7858	10	2
Listeria monocytogenes EGD-e	10	2
Listeria monocytogenes F6900	10	2
Listeria monocytogenes Finland 1988	10	2
Listeria monocytogenes FSL J2-064	10	3
Listeria monocytogenes FSL J2-071	8	2
Listeria monocytogenes FSL N1-017	10	2

Listeria monocytogenes FSL N3-165	10	2
Listeria monocytogenes FSL R2-561	10	2
Listeria monocytogenes HCC23	10	2
Listeria monocytogenes HPB2262	10	2
Listeria monocytogenes J0161, FSL R2-499	10	2
Listeria monocytogenes J2818	10	2
Listeria monocytogenes M7	10	2
Listeria monocytogenes serotype 1/2c SLCC 2372	10	2
Listeria monocytogenes serotype 1/2c SLCC 2755	10	2
Listeria monocytogenes SLCC 2378	11	2
Listeria monocytogenes SLCC 2540	10	2
Listeria seeligeri sv 1/2b, SLCC3954	10	2
Listeria welshimeri sv 6b, SLCC5334	10	2
Lyngbya sp. CCY 8106	6	1
Lysinibacillus fusiformis ZB2	8	2
Lysinibacillus fusiformis ZC1	8	2
Lysinibacillus sphaericus C3-41	4	1
Mahella australiensis 50-1 BON, DSM 15567	5	1
Marichromatium purpuratum 984	2	2
Marinimicrobia bacterium SCGC AAA257-N23 (Etoliko_001_146)	3	3
Marinobacter aquaeolei VT8	5	1
Marvinbryantia formatexigens I-52, DSM 14469	6	1
Mastigocladopsis repens PCC 10914	7	1
Mastigocoleus testarum BC008	6	1
Megasphaera cerevisiae DSM 20462	10	3
Megasphaera genomosp. type_1 28L	3	1
Megasphaera genomosp. UPII 135-E	3	1
Methylibium petroleiphilum PM1	3	1
Microchaete sp. PCC 7126	6	1
Microcoleus chthonoplastes PCC 7420	6	1
Microcoleus sp. PCC 7113	6	1
Microcoleus vaginatus FGP-2	5	1
Microcoleus vaginatus PCC 9802	5	1

Microcystis aeruginosa NIES-843	6	1
Micromonospora sp. CNB394	2	1
Mycobacterium chubuense NBB4	2	1
Mycobacterium fortuitum fortuitum DSM 46621	2	1
Mycobacterium gilvum PYR-GCK	2	1
Mycobacterium gilvum Spyr1	2	1
Mycobacterium hassiacum DSM 44199	2	1
Mycobacterium phlei RIVM601174	2	1
Mycobacterium smegmatis MC2 155	2	1
Mycobacterium smegmatis MC2 155	2	1
Mycobacterium sp. JLS	2	1
Mycobacterium sp. KMS	2	1
Mycobacterium sp. MCS	2	1
Mycobacterium sp. URHD0025	2	1
Mycobacterium vanbaalenii PYR-1	2	1
Nakamurella multipartita Y-104, DSM 44233	3	1
Nitriliruptor alkaliphilus DSM 45188	3	1
Nitrobacter hamburgensis X14	3	2
Nitrobacter sp. Nb-311A	3	2
Nitrobacter winogradskyi Nb-255	3	2
Nitrococcus mobilis Nb-231	4	2
Nitrosomonas eutropha C91	3	2
Nocardioides sp. JS614	3	1
Nodosilinea nodulosa PCC 7104	7	1
Nodularia spumigena CCY9414	6	1
Nostoc azollae 0708	7	1
Nostoc punctiforme PCC 73102	6	1
Nostoc sp. PCC 7107	6	1
Nostoc sp. PCC 7120	6	1
Nostoc sp. PCC 7524	7	1
Olsenella uli VPI, DSM 7084	8	1
Opitutaceae sp. TAV1	3	3
Opitutaceae sp. TAV2	3	3

Opitutaceae sp. TAV5	3	3
Opitutus terrae PB90-1	4	3
Oscillatoria acuminata PCC 6304	6	1
Oscillatoria formosa PCC 6407	5	1
Oscillatoria nigro-viridis PCC 7112	5	1
Oscillatoria sp. PCC 10802	5	1
Oscillatoria sp. PCC 6506	5	1
Oscillatoriales sp. JSC-1	7	1
Oscillatoriales sp. JSC-12	6	1
Oscillibacter valericigenes Sjm18-20	6	1
Paenibacillus alginolyticus DSM 5050	4	1
Paenibacillus alvei DSM 29	12	2
Paenibacillus assamensis DSM 18201	5	1
Paenibacillus dendritiformis C454	12	2
Paenibacillus larvae larvae BRL-230010	5	1
Paenibacillus sp. HW567	3	1
Paenibacillus taiwanensis DSM 18679	5	1
Paenibacillus terrigena DSM 21567	4	1
Pectobacterium sp. SCC3193	4	1
Pectobacterium wasabiae WPP163	4	1
Pediococcus acidilactici 7_4	5	1
Pediococcus claussenii P06, ATCC BAA-344	5	1
Pelosinus sp. HCF1	6	1
Peptoniphilus asaccharolyticus UW 228, DSM 20463	4	1
Peptoniphilus harei ACS-146-V-Sch2b	4	1
Peptoniphilus indolicus ATCC 29427	3	1
Peptoniphilus sp. F0436	6	1
Photobacterium profundum 3TCK	5	1
Pirellula staleyi DSM 6068	3	3
Planococcus donghaensis MPA1U2	4	1
Pleurocapsa sp. PCC 7319	7	1
Pleurocapsa sp. PCC 7327	7	1
Poribacteria bacterium WGA-4CII (final_clean_version)	1	0

Prochlorococcus marinus AS9601	2	2
Prochlorococcus marinus marinus CCMP 1375	3	2
Prochlorococcus marinus MIT 9211	3	2
Prochlorococcus marinus MIT 9215	2	2
Prochlorococcus marinus MIT 9301	2	2
Prochlorococcus marinus MIT 9303	3	2
Prochlorococcus marinus MIT 9312	2	2
Prochlorococcus marinus MIT 9313	3	2
Prochlorococcus marinus MIT 9515	2	2
Prochlorococcus marinus MIT9202	2	2
Prochlorococcus marinus NATL1A	3	2
Prochlorococcus marinus NATL2A	3	2
Prochlorococcus marinus pastoris CCMP 1986	2	2
Prochlorococcus sp. CC9311	3	2
Prochlorococcus sp. CC9605	3	2
Prochlorococcus sp. CC9902	3	2
Prochlorococcus sp. WH 7803	3	2
Prochlorococcus sp. WH8102	3	2
Prochlorothrix hollandica PCC 9006	6	1
Propionibacterium acidifaciens DSM 21887	5	1
Propionibacterium freudenreichii shermanii CIRM-BIA1	5	1
Propionimicrobium lymphophilum DSM 4903	5	1
Proteocatella sphenisci DSM 23131	4	1
Proteus mirabilis ATCC 29906	4	1
Proteus mirabilis HI4320	4	1
Proteus penneri ATCC 35198	4	1
Providencia alcalifaciens DSM 30120	4	1
Providencia rettgeri DSM 1131	4	1
Providencia rustigianii DSM 4541	4	1
Pseudanabaena sp. PCC 6802	6	1
Pseudanabaena sp. PCC 7367	6	1
Pseudanabaena sp. PCC 7429	5	1
Pseudoflavonifractor capillosus ATCC 29799	12	2

Pseudomonas sp. M1	5	1
Pseudonocardia asaccharolytica DSM 44247	5	2
Pseudonocardia dioxanivorans CB1190	3	1
Pseudonocardia spinosispora DSM 44797	4	2
Pseudoramibacter alactolyticus ATCC 23263	12	2
Psychrilyobacter atlanticus DSM 19335	5	1
Psychromonas arctica DSM 14288	5	1
Psychromonas hadalis ATCC BAA-638	5	1
Raphidiopsis brookii D9	6	1
Rhodobacter capsulatus SB1003	4	1
Rhodobacter sphaeroides WS8N	3	1
Rhodococcus erythropolis SK121	2	1
Rhodococcus jostii RHA1	4	2
Rhodopseudomonas palustris BisB18	5	1
Rhodospirillum rubrum F11	5	1
Rhodospirillum rubrum S1, ATCC 11170	5	1
Rivularia sp. PCC 7116	6	1
Roseburia inulinivorans DSM 16841	6	1
Ruminococcus gauvreauii DSM 19829	10	2
Ruminococcus gnavus ATCC 29149	10	1
Ruminococcus obeum A2-162	10	1
Ruminococcus obeum ATCC 29174	10	1
Ruminococcus sp. 5_1_39BFAA	10	1
Ruminococcus sp. SR1/5	10	1
Ruminococcus torques L2-14	10	1
Salmonella enterica Agona SL483	9	2
Salmonella enterica arizonae sv 62:z4,z23 RSK2980	10	2
Salmonella enterica enetrica sv. S4,[5],12:i: CVM23701	10	2
Salmonella enterica enterica Heidelberg SL486	10	2
Salmonella enterica enterica sv Choleraesuis SC-B67	10	2
Salmonella enterica enterica sv Enteritidis P125109	10	2
Salmonella enterica enterica sv Gallinarum 287/91	10	2
Salmonella enterica enterica sv Hadar RI_05P066	10	2

Salmonella enterica enterica sv Javiana GA_MM04042433	10	2
Salmonella enterica enterica sv Kentucky CDC 191	10	2
Salmonella enterica enterica sv Kentucky CVM29188	10	2
Salmonella enterica enterica sv Newport SL317	10	2
Salmonella enterica enterica sv Paratyphi A, ATCC 9150	10	2
Salmonella enterica enterica sv Paratyphi B, SPB7	10	2
Salmonella enterica enterica sv Saint Paul SARA23	14	2
Salmonella enterica enterica sv Saint Paul SARA29	10	2
Salmonella enterica enterica sv Schwarzengrund CVM19633	9	1
Salmonella enterica enterica sv Schwarzengrund SL480	10	2
Salmonella enterica enterica sv Tennessee CDC07-0191	10	2
Salmonella enterica enterica sv Typhi 404ty	10	2
Salmonella enterica enterica sv Typhi AG3	12	2
Salmonella enterica enterica sv Typhi CT18	10	1
Salmonella enterica enterica sv Typhi E00-7866	9	2
Salmonella enterica enterica sv Typhi E01-6750	10	1
Salmonella enterica enterica sv Typhi E02-1180	9	2
Salmonella enterica enterica sv Typhi E98-0664	12	2
Salmonella enterica enterica sv Typhi E98-2068	11	3
Salmonella enterica enterica sv Typhi E98-3139	9	2
Salmonella enterica enterica sv Typhi J185	10	2
Salmonella enterica enterica sv Typhi M223	10	2
Salmonella enterica enterica sv Typhi Ty2	10	1
Salmonella enterica enterica sv Typhimurium 14028S	10	2
Salmonella enterica enterica sv Typhimurium 4/74	10	2
Salmonella enterica enterica sv Typhimurium D23580	9	2
Salmonella enterica enterica sv Typhimurium LT2	10	2
Salmonella enterica enterica sv Typhimurium SL1344	10	2
Salmonella enterica enterica sv Typhimurium T000240	10	2
Salmonella enterica enterica sv Typhimurium TN061786	10	2
Salmonella enterica enterica sv Typhimurium UK-1	10	2
Salmonella enterica enterica sv Virchow SL491	10	2
Salmonella enterica enterica sv Weltevreden HI_N05-537	11	2

Salmonella enterica enterica sv. Enteritidis 22510-1	10	2
Salmonella enterica enterica sv. Enteritidis 485549-17	10	2
Salmonella enterica enterica sv. Enteritidis 50-3079	10	2
Salmonella enterica enterica sv. Enteritidis 58-6482	10	2
Salmonella enterica enterica sv. Enteritidis 596866-22	10	2
Salmonella enterica enterica sv. Enteritidis 596866-70	10	2
Salmonella enterica enterica sv. Enteritidis 607307-6	10	2
Salmonella enterica enterica sv. Enteritidis 622731-39	10	2
Salmonella enterica enterica sv. Enteritidis 629164-26	10	2
Salmonella enterica enterica sv. Enteritidis 629164-37	10	2
Salmonella enterica enterica sv. Enteritidis 639016-6	11	2
Salmonella enterica enterica sv. Enteritidis 639672-46	10	2
Salmonella enterica enterica sv. Enteritidis 639672-50	10	2
Salmonella enterica enterica sv. Enteritidis 640631	10	2
Salmonella enterica enterica sv. Enteritidis 648905 5-18	10	2
Salmonella enterica enterica sv. Enteritidis 77-0424	10	2
Salmonella enterica enterica sv. Enteritidis 77-1427	10	2
Salmonella enterica enterica sv. Enteritidis 77-2659	10	2
Salmonella enterica enterica sv. Enteritidis 78-1757	10	2
Salmonella enterica enterica sv. Enteritidis 8b-1	10	2
Salmonella enterica enterica sv. Gallinarum RKS5078	10	1
Salmonella enterica enterica sv. Heidelberg B182	10	1
Salmonella enterica enterica sv. Heidelberg CFSAN00322	10	2
Salmonella enterica enterica sv. Heidelberg CFSAN00325	10	2
Salmonella enterica enterica sv. Heidelberg CFSAN00326	10	2
Salmonella enterica enterica sv. Newport Levine 1	10	2
Salmonella enterica enterica sv. Newport Levine 15	10	2
Salmonella enterica enterica sv. Typhi P-stx-12	10	2
Salmonella enterica enterica sv. Typhimurium 798	10	2
Salmonella enterica enterica sv. Typhimurium STm1	11	2
Salmonella enterica enterica sv. Typhimurium STm11	10	2
Salmonella enterica enterica sv. Typhimurium STm12	10	2
Salmonella enterica enterica sv. Typhimurium STm2	10	2

Salmonella enterica enterica sv. Typhimurium STm3	10	2
Salmonella enterica enterica sv. Typhimurium STm4	10	2
Salmonella enterica enterica sv. Typhimurium STm5	10	1
Salmonella enterica enterica sv. Typhimurium STm6	10	2
Salmonella enterica enterica sv. Typhimurium STm8	10	2
Salmonella enterica enterica sv. Typhimurium STm9	10	2
Salmonella enterica Paratyphi C RKS4594	9	1
Salmonella enterica sv Dublin CT_02021853	9	1
Salmonella enterica sv Heidelberg SL476, CVM30485	9	2
Salmonella enterica sv Newport SL254	9	2
Salmonella enterica sv Paratyphi A AKU_12601	10	2
Scytonema hofmanni UTEX 2349	6	1
Sebaldella termitidis ATCC 33386	8	1
Shewanella benthica KT99	5	1
Shewanella putrefaciens CN-32	4	1
Shewanella sp. W3-18-1	4	1
Shigella boydii BS512, CDC 3083-94	4	1
Shigella boydii Sb227	4	1
Shigella dysenteriae 1012	4	1
Shigella dysenteriae 225-75	4	1
Shigella flexneri K-315	4	1
Shigella sonnei 3233-85	10	2
Shigella sonnei Ss046	10	2
Shigella sp. D9	4	1
Shimwellia blattae DSM 4481	4	1
Shuttleworthia satelles DSM 14600	7	1
Singulisphaera acidiphila MOB10, DSM 18658	3	3
Sodalis glossinidius morsitans	1	1
Spirochaeta alkalica DSM 8900	4	4
Spirulina major PCC 6313	6	1
Spirulina subsalsa PCC 9445	6	1
Sporosarcina newyorkensis 2681	4	1
Sporosarcina ureae DSM 2281	4	1

Stanieria cyanosphaera PCC 7437	7	1
Streptococcus australis ATCC 700641	6	1
Streptococcus castoreus DSM 17536	7	1
Streptococcus devriesei DSM 19639	5	1
Streptococcus dysgalactiae equisimilis D166B, ATCC 12394	7	1
Streptococcus intermedius JTH08	4	1
Streptococcus massiliensis DSM 18628	8	1
Streptococcus merionis DSM 19192	13	2
Streptococcus ovis DSM 16829	8	1
Streptococcus parasanguinis ATCC 903	5	1
Streptococcus sanguinis SK36	6	1
Streptococcus sanguinis VMC66	6	1
Streptomyces scabrisporus DSM 41855	2	1
Synechococcus elongatus PCC 6301	5	1
Synechococcus elongatus PCC 7942	5	1
Synechococcus sp. BL107	3	2
Synechococcus sp. CB0101	3	2
Synechococcus sp. CB0205	3	2
Synechococcus sp. CC9616	3	2
Synechococcus sp. JA-2-3B'a(2-13)	4	1
Synechococcus sp. JA-3-3Ab	4	1
Synechococcus sp. PCC 6312	6	1
Synechococcus sp. PCC 7002	6	1
Synechococcus sp. PCC 7335	6	1
Synechococcus sp. PCC 7336	6	1
Synechococcus sp. PCC 7502	6	1
Synechococcus sp. RCC 307	3	2
Synechococcus sp. RS9916	3	2
Synechococcus sp. RS9917	3	2
Synechococcus sp. WH 8016	3	2
Synechococcus sp. WH 8109	3	2
Synechococcus sp. WH5701	4	2
Synechococcus sp. WH7805	3	2

Synechocystis sp. GT-S, PCC 6803	6	1
Synechocystis sp. PCC 6803	6	1
Synechocystis sp. PCC 6803, GT-I	6	1
Synechocystis sp. PCC 6803, PCC-N	6	1
Synechocystis sp. PCC 6803, PCC-P	6	1
Synechocystis sp. PCC 7509	6	1
Synergistes sp. 3_1_syn1	6	1
Synergistetes bacterium SGP1	3	1
Thermacetogenium phaeum PB, DSM 12270	5	1
Thermanaeromonas toyohensis ToBE, DSM 14490	3	1
Thermanaerovibrio acidaminovorans Su883, DSM 6589	5	1
Thermanaerovibrio velox Z-9701, DSM 12556	5	1
Thermincola potens JR	11	2
Thermithiobacillus tepidarius DSM 3134	2	2
Thermoanaerobacter ethanolicus JW 200	5	2
Thermoanaerobacter sp. X513	5	1
Thermoanaerobacter sp. X514	5	1
Thermoanaerobacter sp. X561	5	1
Thermoanaerobacterium saccharolyticum JW/SL-YS485, DSM 8691	5	1
Thermoanaerobacterium thermosaccharolyticum M0795	4	1
Thermoanaerobacterium xylanolyticum LX-11, DSM 7097	5	1
Thermocrispum agreste DSM 44070	2	1
Thermosediminibacter oceani JW/IW-1228P, DSM 16646	4	1
Thermosynechococcus elongatus BP-1	6	1
Thermovirga lienii Cas60314, DSM 17291	7	1
Thioalkalimicrobium aerophilum AL3	3	2
Thioalkalimicrobium cyclicum ALM1	3	2
Thioalkalivibrio nitratireducens DSM 14787	4	2
Thioalkalivibrio paradoxus ARh 1	4	2
Thioalkalivibrio sp. AKL10	4	2
Thioalkalivibrio sp. AKL11	4	2
Thioalkalivibrio sp. AKL12	4	2
Thioalkalivibrio sp. AKL17	2	2

Thioalkalivibrio sp. AKL19	4	2
Thioalkalivibrio sp. AKL3	4	2
Thioalkalivibrio sp. AKL6	4	2
Thioalkalivibrio sp. AKL7	4	2
Thioalkalivibrio sp. AKL8	4	2
Thioalkalivibrio sp. AKL9	4	2
Thioalkalivibrio sp. AL21	4	2
Thioalkalivibrio sp. AL5	4	2
Thioalkalivibrio sp. ALD1	4	2
Thioalkalivibrio sp. ALE10	2	2
Thioalkalivibrio sp. ALE11	2	2
Thioalkalivibrio sp. ALE12	4	2
Thioalkalivibrio sp. ALE14	4	2
Thioalkalivibrio sp. ALE16	4	2
Thioalkalivibrio sp. ALE17	4	2
Thioalkalivibrio sp. ALE18	4	2
Thioalkalivibrio sp. ALE19	3	2
Thioalkalivibrio sp. ALE20	2	2
Thioalkalivibrio sp. ALE21	4	2
Thioalkalivibrio sp. ALE22	4	2
Thioalkalivibrio sp. ALE23	4	2
Thioalkalivibrio sp. ALE25	4	2
Thioalkalivibrio sp. ALE27	4	2
Thioalkalivibrio sp. ALE28	4	2
Thioalkalivibrio sp. ALE30	4	2
Thioalkalivibrio sp. ALE31	4	2
Thioalkalivibrio sp. ALE6	4	2
Thioalkalivibrio sp. ALE9	4	2
Thioalkalivibrio sp. ALgr1	4	2
Thioalkalivibrio sp. ALgr3	2	2
Thioalkalivibrio sp. ALgr5	2	2
Thioalkalivibrio sp. ALJ10	4	2
Thioalkalivibrio sp. ALJ11	4	2

Thioalkalivibrio sp. ALJ12	4	2
Thioalkalivibrio sp. ALJ15	4	2
Thioalkalivibrio sp. ALJ16	4	2
Thioalkalivibrio sp. ALJ17	3	2
Thioalkalivibrio sp. ALJ2	4	2
Thioalkalivibrio sp. ALJ20	4	2
Thioalkalivibrio sp. ALJ21	4	2
Thioalkalivibrio sp. ALJ24	2	2
Thioalkalivibrio sp. ALJ3	4	2
Thioalkalivibrio sp. ALJ4	4	2
Thioalkalivibrio sp. ALJ5	4	2
Thioalkalivibrio sp. ALJ6	4	2
Thioalkalivibrio sp. ALJ7	4	2
Thioalkalivibrio sp. ALJ8	4	2
Thioalkalivibrio sp. ALJ9	4	2
Thioalkalivibrio sp. ALJT	4	2
Thioalkalivibrio sp. ALM2T	4	2
Thioalkalivibrio sp. ALMg11	4	2
Thioalkalivibrio sp. ALMg13-2	4	2
Thioalkalivibrio sp. ALMg2	4	2
Thioalkalivibrio sp. ALMg3	4	2
Thioalkalivibrio sp. ALMg9	4	2
Thioalkalivibrio sp. ALR17-21	2	2
Thioalkalivibrio sp. ALRh	4	2
Thioalkalivibrio sp. ALSr1	4	2
Thioalkalivibrio sp. ARh3	4	2
Thioalkalivibrio sp. ARh4	4	2
Thioalkalivibrio sp. ARh5	4	2
Thioalkalivibrio sp. HL-Eb18	4	2
Thioalkalivibrio sp. K90mix	4	2
Thioalkalivibrio sulfidophilus HL-EbGR7	4	2
Thioalkalivibrio thiocyanoxidans ARh 4	4	2
Thioalkalivibrio thiocyanoxidans ARh2	4	2

Thioalkalivibrio versutus AL2	8	4
Thiobacillus denitrificans ATCC 25259	3	2
Thiobacillus thioparus DSM 505	3	2
Thiocapsa marina 5811, DSM 5653	3	2
Thioflavicoccus mobilis 8321	3	2
Thiomicrospira crunogena XCL-2	4	2
Thiomicrospira halophila DSM 15072	4	2
Thiomicrospira sp. JR2	4	2
Thiomicrospira sp. KP2	4	2
Thiomonas arsenitoxydans 3As	4	2
Thiomonas intermedia K12	4	2
Thiorhodococcus drewsii AZ1	3	2
Thiorhodovibrio sp. 970	3	2
Thiothrix nivea JP2, DSM 5205	3	2
Tolumonas auensis TA 4, DSM 9187	6	1
Trichodesmium erythraeum IMS101	6	1
Veillonella dispar ATCC 17748	6	1
Veillonella magna DSM 19857	9	2
Veillonella sp. 6_1_27	6	1
Veillonella sp. F0422	5	1
Veillonella sp. oral taxon 158 F0412	6	1
Verminephrobacter eiseniae EF01-2	2	1
Verrucomicrobia bacterium SCGC AAA027-I19	3	3
Verrucomicrobia bacterium SCGC AAA164-E04	3	3
Verrucomicrobiales sp. DG1235	3	3
Verrucosispora maris AB-18-032	2	1
Vibrio furnissii 2510/74, NCTC 11218	4	1
Vibrio litoralis DSM 17657	5	1
Vibrio sp. EJY3	5	1
Xenococcus sp. PCC 7305	6	1
Yersinia aldovae ATCC 35236	3	1
Yersinia bercovieri ATCC 43970	6	1
Yersinia enterocolitica enterocolitica 8081	6	1

Yersinia enterocolitica IP 10393	6	1
Yersinia enterocolitica palearctica 105.5R(r) (O:9)	6	1
Yersinia enterocolitica palearctica sv O:3/4 Y11	6	1
Yersinia frederiksenii ATCC 33641	6	1
Yersinia intermedia ATCC 29909	10	2
Yersinia kristensenii ATCC 33638	10	2
Yersinia mollaretii ATCC 43969	6	1
