# Supplementary material for: The Candidate Phylum Poribacteria by Single-Cell Genomics: New Insights into Phylogeny, Cell-Compartmentation, Eukaryote-Like Repeat Proteins, and Other Genomic Features
Source: PLoS One. 2014 Jan 31;9(1):e87353. doi: 10.1371/journal.pone.0087353 (PMC3909097; doi:10.1371/journal.pone.0087353)
Supplement: Table S7 — Overview of gene copy numbers (no) and percentage of genes per total genes on genome (%) of repeat proteins and eukaryote like protein domain genes on poribacterial SAGs and the maximum and average number of gene copies found on X finished genomes of marine free-living bacteria (n = 101). (PDF) [file pone.0087353.s007.pdf]

Table S7: Overview of gene copy numbers (no) and percentage of genes per total genes on genome (%) of repeat proteins and eukaryote like protein domain genes on poribacterial SAGs and the maximum and average number of gene copies found on X finished genomes of marine free-living bacteria (n=101).

Genome Name			4C	4E	4G	3A	3G	4CII	Max	Average
Taxon OID			2265129007	2265129006	2265129008	2265129010	2265129009	2265129011	Marine free living	Marine free living
Genome Size (bp)			1629923	3647669	189191	414219	5441554	543453		
Genes on genome			1634	3319	178	505	4820	478		
pfam00023	Ankyrin repeat	no	0	0	0	0	1	0	4	0.21
		%	0.00	0.00	0.00	0.00	0.02	0.00	0.10	0.00
pfam00028	Cadherin domain	no	3	2	0	0	3	0	4	0.19
		%	0.18	0.06	0.00	0.00	0.06	0.00	0.06	0.00
pfam00041	Fibronectin type III domain	no	0	0	0	0	3	0	26	0.39
		%	0.00	0.00	0.00	0.00	0.06	0.00	0.13	0.01
pfam00058	Low-density lipoprotein receptor repeat class B	no	1	1	0	0	5	0	1	0.02
		%	0.06	0.03	0.00	0.00	0.10	0.00	0.03	0.00
pfam00069	Protein kinase domain	no	0	1	0	0	3	0	252	7.43
		%	0.00	0.03	0.00	0.00	0.06	0.00	2.95	0.13
pfam00400	WD domain, G-beta repeat	no	34	24	3	4	63	5	36	2.14
		%	2.08	0.72	1.69	0.79	1.31	1.05	0.52	0.05
pfam00404	Dockerin type I repeat	no	3	2	0	0	7	1	20	0.20
		%	0.18	0.06	0.00	0.00	0.15	0.21	0.27	0.00
pfam00515	Tetratricopeptide repeat	no	3	17	0	0	24	3	45	2.20

		%	0.18	0.51	0.00	0.00	0.50	0.63	0.87	0.06
pfam01011	PQQ enzyme repeat	no	0	0	1	0	1	0	4	0.19
		%	0.00	0.00	0.56	0.00	0.02	0.00	0.05	0.00
pfam01436	NHL repeat	no	1	3	0	0	5	1	5	0.24
		%	0.06	0.09	0.00	0.00	0.10	0.21	0.08	0.00
pfam02368	Bacterial Ig-like domain (group 2)	no	0	5	0	0	0	0	7	0.34
		%	0.00	0.15	0.00	0.00	0.00	0.00	0.25	0.01
pfam02369	Bacterial Ig-like domain (group 1)	no	1	0	0	0	1	0	6	0.42
		%	0.06	0.00	0.00	0.00	0.02	0.00	0.13	0.01
pfam05833	Fibronectin-binding protein A N-terminus (FbpA)	no	1	1	0	0	1	0	1	0.34
		%	0.06	0.03	0.00	0.00	0.02	0.00	0.06	0.01
pfam07593	ASPIC and UnbV	no	2	6	2	0	13	0	15	0.89
		%	0.12	0.18	1.12	0.00	0.27	0.00	0.59	0.03
pfam07719	Tetratricopeptide repeat	no	3	4	2	1	16	3	17	1.85
		%	0.18	0.12	1.12	0.20	0.33	0.63	0.27	0.05
pfam08238	Sel1 repeat	no	0	5	0	0	1	0	14	1.79
		%	0	0.15	0	0	0.02	0	0.37	0.05
pfam09976	Tetratricopeptide repeat	no	0	1	0	0	1	0	2	0.46
		%	0.00	0.03	0.00	0.00	0.02	0.00	0.04	0.01
pfam12245	Bacterial Ig-like domain (group 3)	no	0	1	0	0	0	0	2	0.08
		%	0.00	0.03	0.00	0.00	0.00	0.00	0.04	0.00

pfam12796	Ankyrin repeats (3 copies)	no	1	4	0	0	6	0	16	1.27
		%	0.06	0.12	0.00	0.00	0.12	0.00	0.42	0.03
pfam12799	Leucine Rich repeats (2 copies)	no	9	21	0	1	25	0	102	0.71
		%	0.55	0.63	0.00	0.20	0.52	0.00	0.30	0.01
pfam13174	Tetratricopeptide repeat	no	2	6	2	1	8	2	5	0.98
		%	0.12	0.18	1.12	0.20	0.17	0.42	0.13	0.02
pfam13176	Tetratricopeptide repeat	no	0	0	0	0	1	0	6	0.21
		%	0.00	0.00	0.00	0.00	0.02	0.00	0.12	0.00
pfam13181	Tetratricopeptide repeat	no	2	4	0	0	8	1	12	0.75
		%	0.12	0.12	0.00	0.00	0.17	0.21	0.31	0.02
pfam13205	Bacterial Ig-like domain	no	2	0	0	0	4	0	5	0.34
		%	0.12	0.00	0.00	0.00	0.08	0.00	0.08	0.01
pfam13360	PQQ-like domain	no	2	10	1	0	11	0	52	3.18
		%	0.12	0.30	0.56	0.00	0.23	0.00	1.27	0.09
pfam13371	Tetratricopeptide repeat	no	0	1	0	1	2	0	6	0.68
		%	0.00	0.03	0.00	0.20	0.04	0.00	0.15	0.02
pfam13374	Tetratricopeptide repeat	no	0	1	0	0	0	0	13	0.47
		%	0.00	0.03	0.00	0.00	0.00	0.00	0.25	0.01
pfam13414	Tetratricopeptide repeat	no	16	45	3	4	83	7	80	9.83
		%	0.98	1.36	1.69	0.79	1.72	1.46	1.93	0.28
pfam13424	Tetratricopeptide repeat	no	1	1	0	1	4	1	103	2.21

		%	0.06	0.03	0.00	0.20	0.08	0.21	0.63	0.05
pfam13428	Tetratricopeptide repeat	no	0	0	0	0	2	0	4	0.19
		%	0.00	0.00	0.00	0.00	0.04	0.00	0.04	0.00
pfam13429	Tetratricopeptide repeat	no	0	0	0	0	1	0	2	0.16
		%	0.00	0.00	0.00	0.00	0.02	0.00	0.05	0.00
pfam13431	Tetratricopeptide repeat	no	0	0	0	0	1	0	1	0.01
		%	0.00	0.00	0.00	0.00	0.02	0.00	0.02	0.00
pfam13432	Tetratricopeptide repeat	no	1	3	1	2	9	0	33	2.45
		%	0.06	0.09	0.56	0.40	0.19	0.00	0.63	0.07
pfam13516	Leucine Rich repeat	no	1	1	0	0	1	0	6	0.19
		%	0.06	0.03	0.00	0.00	0.02	0.00	0.09	0.00
pfam13517	Repeat domain in Vibrio. Colwellia, Bradyrhizobium and Shewanella	no	4	6	2	0	18	0	28	2.38
		%	0.24	0.18	1.12	0.00	0.37	0.00	0.89	0.06
pfam13570	PQQ-like domain	no	1	2	0	0	1	0	6	0.29
		%	0.06	0.06	0.00	0.00	0.02	0.00	0.15	0.01
pfam13637	Ankyrin repeats (many copies)	no	0	0	0	0	1	0	2	0.06
		%	0.00	0.00	0.00	0.00	0.02	0.00	0.04	0.00
pfam13750	Bacterial Ig-like domain (group 3)	no	0	0	0	0	1	0	2	0.02
		%	0.00	0.00	0.00	0.00	0.02	0.00	0.04	0.00
pfam13754	Bacterial Ig-like domain (group 3)	no	0	1	0	0	0	0	4	0.26
		%	0.00	0.03	0.00	0.00	0.00	0.00	0.08	0.01

pfam13855	Leucine rich repeat	no	0	2	0	0	0	0	96	0.60
		%	0.00	0.06	0.00	0.00	0.00	0.00	0.44	0.01
pfam13857	Ankyrin repeats (many copies)	no	1	0	0	0	0	0	2	0.11
		%	0.06	0.00	0.00	0.00	0.00	0.00	0.05	0.00
