# Supplementary material for: The Candidate Phylum Poribacteria by Single-Cell Genomics: New Insights into Phylogeny, Cell-Compartmentation, Eukaryote-Like Repeat Proteins, and Other Genomic Features
Source: PLoS One. 2014 Jan 31;9(1):e87353. doi: 10.1371/journal.pone.0087353 (PMC3909097; doi:10.1371/journal.pone.0087353)
Supplement: Table S8 — List of all repeat and eukaryote like domain protein encoding genes on poribacterial SAGs. Information is shown as available in IMG/MER system. THM: number of predicted transmembrane helicies. SP: signal peptide predicted yes (Y) or no (N). (PDF) [file pone.0087353.s008.pdf]

Table S8: List of all repeat and eukaryote like domain protein encoding genes on poribacterial SAGs. Information is shown as available in IMG/MER system. THM: number of predicted transmembrane helicies. SP: signal peptide predicted yes (Y) or no (N).

Genome	Gene ID	Locus Tag	Gene Product Name	COG	Pfam	Tigrfam	Enzyme	KO	TMH	SP
3A	2265147464	POR_0122.00 000020	FOG: WD40 repeat	COG2319 FOG: WD40 repeat	pfam00400 WD40				0	Y
	2265147482	POR_0125.00 000020	Leucine-rich repeat (LRR) protein	COG4886 Leucine-rich repeat (LRR) protein	pfam12799 LRR_4 pfam00932 LTD				0	N
	2265147526	POR_0167.00 000040	Tfp pilus assembly protein PilF	COG3063 Tfp pilus assembly protein PilF	pfam13432 TPR_16 pfam13371 TPR_9 pfam13414 TPR_11				1	N
	2265147540	POR_0192.00 000020	hypothetical protein		pfam13174 TPR_6 pfam13432 TPR_16				0	N
	2265147551	POR_0206.00 000020	hypothetical protein	COG3903 Predicted ATPase	pfam07719 TPR_2				0	N
	2265147671	POR_0422.00 000010	FOG: WD40 repeat	COG2319 FOG: WD40 repeat	pfam00400 WD40				0	N
	2265147722	POR_0471.00 000010	FOG: WD40 repeat	COG2319 FOG: WD40 repeat	pfam00400 WD40 pfam00963 Cohesin				0	N
	2265147900	POR_0079.00 000020	Tetratricopeptide repeat.	COG5010 Flp pilus assembly protein TadD, contains TPR	pfam13414 TPR_11				0	N

				repeats						
	2265147902	POR_0079.00 000040	Tetratricopeptide repeat.	COG5010 Flp pilus assembly protein TadD, contains TPR repeats	pfam13414 TPR_11				0	N
	2265147912	POR_0083.00 000070	hypothetical protein	COG0457 FOG: TPR repeat	pfam13435 Cytochrome_C5 54 pfam13424 TPR_12 pfam13414 TPR_11				5	N
	2265147921	POR_0087.00 000040	FOG: WD40 repeat	COG2319 FOG: WD40 repeat	pfam00400 WD40	TIGR04183 Por secretion system C-terminal sorting domain			0	Y
3G	2265142644	POR3G_0003 8	Tfp pilus assembly protein PilF	COG3063 Tfp pilus assembly protein PilF	pfam07494 Reg_prop pfam13414 TPR_11				1	N
	2265142656	POR3G_0005 0	Uncharacterized conserved protein	COG3391 Uncharacterized conserved protein	pfam01436 NHL				0	N
	2265142696	POR3G_0009 0	DNA uptake lipoprotein	COG4105 DNA uptake lipoprotein	pfam13174 TPR_6 pfam13414 TPR_11				0	N
	2265142724	POR3G_0011 8	FOG: WD40 repeat	COG2319 FOG: WD40 repeat	pfam00400 WD40				0	N
	2265142730	POR3G_0012 4	FOG: WD40 repeat	COG2319 FOG: WD40 repeat	pfam00400 WD40 pfam07635				1	Y

					PSCyt1					
	2265142767	POR3G_0016 2	FOG: WD40 repeat	COG2319 FOG: WD40 repeat	pfam00400 WD40				1	N
	2265142809	POR3G_0020 4	FOG: WD40 repeat	COG2319 FOG: WD40 repeat	pfam08662 eIF2A pfam00400 WD40 pfam13583 Reprolysin_4				3	N
	2265142812	POR3G_0020 7	FOG: WD40-like repeat	COG1520 FOG: WD40-like repeat	pfam13360 PQQ_2				0	Y
	2265142893	POR3G_0028 8	FOG: WD40 repeat	COG2319 FOG: WD40 repeat	pfam00400 WD40				0	N
	2265142902	POR3G_0029 7	Serine/threonine protein kinase	COG0515 Serine/threonin e protein kinase	pfam00069 Pkinase				0	N
	2265142931	POR3G_0032 6	FOG: WD40 repeat	COG2319 FOG: WD40 repeat	pfam00400 WD40				0	Y
	2265142952	POR3G_0034 7	Tfp pilus assembly protein PilF	COG3063 Tfp pilus assembly protein PilF	pfam07719 TPR_2 pfam00515 TPR_1 pfam13414 TPR_11				1	N
	2265142982	POR3G_0037 7	Lipoprotein NlpI, contains TPR repeats	COG4785 Lipoprotein NlpI, contains TPR repeats	pfam13414 TPR_11				0	N
	2265143012	POR3G_0040 7	Uncharacterized protein conserved in bacteria	COG1729 Uncharacterized protein conserved in	pfam13525 YfiO pfam13181 TPR_8 pfam13174				1	N

				bacteria	TPR_6						
	2265143015	POR3G_0041 0	hypothetical protein		pfam13205 Big_5					0	Y
	2265143026	POR3G_0042 1	Cadherin domain.		pfam00028 Cadherin					0	N
	2265143051	POR3G_0044 6	Tfp pilus assembly protein PilF	COG3063 Tfp pilus assembly protein PilF	pfam13414 TPR_11 pfam00515 TPR_1					0	N
	2265143127	POR3G_0052 2	Tfp pilus assembly protein PilF	COG3063 Tfp pilus assembly protein PilF	pfam13414 TPR_11 pfam07719 TPR_2					1	N
	2265143151	POR3G_0054 6	Tfp pilus assembly protein PilF	COG3063 Tfp pilus assembly protein PilF	pfam07719 TPR_2					0	N
	2265143165	POR3G_0056 0	Tfp pilus assembly protein PilF	COG3063 Tfp pilus assembly protein PilF	pfam13414 TPR_11 pfam00515 TPR_1					0	N
	2265143168	POR3G_0056 3	Tfp pilus assembly protein PilF	COG3063 Tfp pilus assembly protein PilF	pfam03683 UPF0175 pfam00515 TPR_1 pfam13414 TPR_11					0	N
	2265143182	POR3G_0057 7	Leucine-rich repeat (LRR) protein	COG4886 Leucine-rich repeat (LRR) protein	pfam12799 LRR_4	TIGR04183 Por secretion system C-terminal sorting domain				0	N
	2265143194	POR3G_0058	FOG: WD40 repeat	COG2319 FOG:	pfam00400 WD40					0	N

		9		WD40 repeat	pfam08662 eIF2A						
	2265143209	POR3G_0060 4	hypothetical protein	COG1520 FOG: WD40-like repeat	pfam13360 PQQ_2					1	Y
	2265143211	POR3G_0060 6	Putative Zn-dependent protease, contains TPR repeats	COG4783 Putative Zn-dependent protease, contains TPR repeats	pfam13414 TPR_11					1	Y
	2265143213	POR3G_0060 8	ASPIC and UnbV.		pfam07593 UnbV_ASPIC pfam13517 VCBS					0	Y
	2265143231	POR3G_0062 6	hypothetical protein		pfam13414 TPR_11					1	N
	2265143312	POR3G_0070 7	hypothetical protein	COG1520 FOG: WD40-like repeat	pfam13360 PQQ_2					1	Y
	2265143317	POR3G_0071 3	Tfp pilus assembly protein PilF	COG3063 Tfp pilus assembly protein PilF	pfam13676 TIR_2 pfam13414 TPR_11					0	N
	2265143324	POR3G_0072 0	RNA polymerase sigma factor, sigma-70 family	COG1595 DNA-directed RNA polymerase specialized sigma subunit, sigma24 homolog	pfam04542 Sigma70_r2 pfam04545 Sigma70_r4 pfam00400 WD40	TIGR02937 RNA polymerase sigma factor, sigma-70 family				0	N
	2265143366	POR3G_0076 2	Flp pilus assembly protein TadD, contains TPR repeats	COG5010 Flp pilus assembly protein TadD,	pfam07719 TPR_2 pfam13414					1	N

				contains TPR repeats	TPR_11					
	2265143378	POR3G_0077 4	FOG: WD40 repeat	COG2319 FOG: WD40 repeat	pfam00400 WD40				0	N
	2265143389	POR3G_0078 5	FOG: WD40 repeat	COG2319 FOG: WD40 repeat	pfam00400 WD40				0	N
	2265143397	POR3G_0079 3	Dockerin type I repeat.		pfam00404 Dockerin_1 pfam11306 DUF3108				0	Y
	2265143407	POR3G_0080 3	FOG: WD40 repeat	COG2319 FOG: WD40 repeat	pfam13860 FlgD_ig pfam00400 WD40	TIGR04183 Por secretion system C-terminal sorting domain			0	Y
	2265143472	POR3G_0086 8	FOG: WD40 repeat	COG2319 FOG: WD40 repeat	pfam13860 FlgD_ig pfam00400 WD40	TIGR04183 Por secretion system C-terminal sorting domain			0	Y
	2265143484	POR3G_0088 0	FOG: WD40-like repeat	COG1520 FOG: WD40-like repeat	pfam13570 PQQ_3 pfam13360 PQQ_2				1	N
	2265143503	POR3G_0089 9	Tetratricopeptide repeat.		pfam13414 TPR_11				1	N
	2265143543	POR3G_0093 9	ASPIC and UnbV.		pfam07593 UnbV_ASPIC pfam13517 VCBS				1	N
	2265143550	POR3G_0094 6	PQQ enzyme repeat.	COG1520 FOG: WD40-like	pfam13360 PQQ_2 pfam01011				3	N

				repeat	PQQ					
	2265143555	POR3G_00951	hypothetical protein	COG4105 DNA uptake lipoprotein	pfam00515 TPR_1 pfam13432 TPR_16				1	Y
	2265143557	POR3G_00953	Cytochrome c biogenesis factor	COG4235 Cytochrome c biogenesis factor	pfam13432 TPR_16 pfam13517 VCBS pfam13428 TPR_14 pfam07593 UnbV_ASPIC				0	N
	2265143559	POR3G_00955	ASPIC and UnbV.		pfam13517 VCBS pfam07593 UnbV_ASPIC				0	Y
	2265143567	POR3G_00963	FG-GAP repeat.		pfam13517 VCBS				0	N
	2265143587	POR3G_00983	FOG: WD40 repeat	COG2319 FOG: WD40 repeat	pfam00400 WD40				0	N
	2265143625	POR3G_01021	Tfp pilus assembly protein PilF	COG3063 Tfp pilus assembly protein PilF	pfam13414 TPR_11				1	N
	2265143655	POR3G_01051	FOG: TPR repeat	COG0457 FOG: TPR repeat	pfam08867 FRG pfam13414 TPR_11				0	N
	2265143667	POR3G_01063	Tetratricopeptide repeat.		pfam13414 TPR_11				0	N
	2265143691	POR3G_01087	IPT/TIG domain.		pfam01833 TIG pfam13205	TIGR03437 Solibacter uncharacterized			1	Y

					Big_5	C-terminal domain				
	2265143695	POR3G_0109 1	Tfp pilus assembly protein PilF	COG3063 Tfp pilus assembly protein PilF	pfam13424 TPR_12 pfam13432 TPR_16 pfam13371 TPR_9 pfam13414 TPR_11				1	N
	2265143741	POR3G_0113 7	Lipoprotein NlpI, contains TPR repeats	COG4785 Lipoprotein NlpI, contains TPR repeats	pfam13414 TPR_11				1	Y
	2265143756	POR3G_0115 2	FOG: TPR repeat	COG0457 FOG: TPR repeat	pfam11074 DUF2779 pfam00515 TPR_1 pfam13414 TPR_11				0	N
	2265143771	POR3G_0116 7	ASPIC and UnbV./FG-GAP repeat.		pfam13517 VCBS pfam07593 UnbV_ASPIC				1	N
	2265143886	POR3G_0128 3	FOG: WD40 repeat	COG2319 FOG: WD40 repeat	pfam00400 WD40				1	N
	2265143887	POR3G_0128 4	FOG: WD40 repeat	COG2319 FOG: WD40 repeat	pfam00400 WD40				0	Y
	2265143929	POR3G_0132 6	ASPIC and UnbV.		pfam07593 UnbV_ASPIC pfam13517 VCBS	TIGR04183 Por secretion system C-terminal sorting domain			0	N

	2265143955	POR3G_0135 2	RNA polymerase sigma factor, sigma-70 family	COG2319 FOG: WD40 repeat	pfam00400 WD40 pfam04542 Sigma70_r2 pfam04545 Sigma70_r4	TIGR02937 RNA polymerase sigma factor, sigma-70 family				0	N
	2265143956	POR3G_0135 3	FOG: WD40 repeat	COG2319 FOG: WD40 repeat	pfam00400 WD40					0	N
	2265143970	POR3G_0136 7	Tfp pilus assembly protein PilF	COG3063 Tfp pilus assembly protein PilF	pfam13414 TPR_11 pfam12950 TaqI_C					0	N
	2265143975	POR3G_0137 2	Cytochrome c biogenesis factor	COG4235 Cytochrome c biogenesis factor	pfam13414 TPR_11					0	N
	2265144026	POR3G_0142 4	FOG: WD40 repeat	COG2319 FOG: WD40 repeat	pfam00400 WD40					0	N
	2265144027	POR3G_0142 5	FOG: WD40 repeat	COG2319 FOG: WD40 repeat	pfam00400 WD40 pfam00404 Dockerin_1					0	N
	2265144028	POR3G_0142 6	FOG: WD40 repeat	COG2319 FOG: WD40 repeat	pfam00400 WD40					1	Y
	2265144029	POR3G_0142 7	FOG: WD40 repeat	COG2319 FOG: WD40 repeat	pfam00400 WD40	TIGR04183 Por secretion system C-terminal sorting domain				1	N
	2265144075	POR3G_0147 3	Flp pilus assembly protein TadD, contains TPR repeats	COG5010 Flp pilus assembly protein TadD, contains TPR	pfam13414 TPR_11					0	N

				repeats						
	2265144109	POR3G_0150 7	Putative Zn-dependent protease, contains TPR repeats	COG4783 Putative Zn-dependent protease, contains TPR repeats	pfam00515 TPR_1 pfam13414 TPR_11				0	N
	2265144130	POR3G_0152 8	FOG: Ankyrin repeat	COG0666 FOG: Ankyrin repeat	pfam13637 Ank_4 pfam12796 Ank_2				0	N
	2265144137	POR3G_0153 5	ASPIC and UnbV.		pfam07593 UnbV_ASPIC pfam13517 VCBS				1	N
	2265144159	POR3G_0155 7	Tfp pilus assembly protein PilF	COG3063 Tfp pilus assembly protein PilF	pfam13428 TPR_14 pfam13181 TPR_8 pfam13414 TPR_11				0	Y
	2265144160	POR3G_0155 8	ASPIC and UnbV./FG-GAP repeat.		pfam13517 VCBS pfam07593 UnbV_ASPIC				0	N
	2265144190	POR3G_0158 8	FOG: WD40 repeat	COG2319 FOG: WD40 repeat	pfam13860 FlgD_ig pfam00963 Cohesin pfam00400 WD40	TIGR04183 Por secretion system C-terminal sorting domain			0	Y
	2265144255	POR3G_0165 3	Tfp pilus assembly protein PilF	COG3063 Tfp pilus assembly protein PilF	pfam13414 TPR_11 pfam07719				0	Y

					TPR_2					
	2265144256	POR3G_01654	ASPIC and UnbV./FG-GAP repeat.		pfam07593 UnbV_ASPIC pfam13517 VCBS				0	Y
	2265144279	POR3G_01677	FOG: WD40 repeat	COG2319 FOG: WD40 repeat	pfam13574 Reprolysin_2 pfam00400 WD40				0	Y
	2265144285	POR3G_01683	FOG: TPR repeat, SEL1 subfamily	COG0790 FOG: TPR repeat, SEL1 subfamily	pfam01844 HNH pfam08238 Sel1				0	N
	2265144294	POR3G_01692	Tfp pilus assembly protein PilF	COG3063 Tfp pilus assembly protein PilF	pfam00515 TPR_1 pfam13414 TPR_11				0	N
	2265144329	POR3G_01727	FOG: WD40 repeat	COG2319 FOG: WD40 repeat	pfam00400 WD40				1	N
	2265144402	POR3G_01800	Tfp pilus assembly protein PilF	COG3063 Tfp pilus assembly protein PilF	pfam00515 TPR_1 pfam13414 TPR_11				0	N
	2265144404	POR3G_01802	FOG: TPR repeat	COG0457 FOG: TPR repeat	pfam13414 TPR_11				0	N
	2265144406	POR3G_01804	Leucine-rich repeat (LRR) protein	COG4886 Leucine-rich repeat (LRR) protein	pfam05345 He_PIG pfam02369 Big_1 pfam13860 FlgD_ig pfam12799 LRR_4	TIGR04183 Por secretion system C-terminal sorting domain			0	N

	2265144417	POR3G_0181 5	Fibronectin type III domain.		pfam00041 fn3				4	Y
	2265144438	POR3G_0183 6	Dockerin type I repeat.		pfam00404 Dockerin_1				0	N
	2265144444	POR3G_0184 2	FOG: WD40 repeat	COG2319 FOG: WD40 repeat	pfam00400 WD40				0	Y
	2265144445	POR3G_0184 3	FOG: WD40 repeat	COG2319 FOG: WD40 repeat	pfam00400 WD40				0	N
	2265144459	POR3G_0185 7	Subtilisin-like serine proteases	COG1404 Subtilisin-like serine proteases	pfam13517 VCBS pfam00082 Peptidase_S8	TIGR04183 Por secretion system C-terminal sorting domain			1	N
	2265144460	POR3G_0185 8	FOG: HEAT repeat	COG1413 FOG: HEAT repeat	pfam13646 HEAT_2 pfam13414 TPR_11				0	N
	2265144470	POR3G_0186 8	FG-GAP repeat.		pfam13517 VCBS pfam01839 FG-GAP				0	N
	2265144491	POR3G_0188 9	FOG: WD40-like repeat	COG1520 FOG: WD40-like repeat	pfam13360 PQQ_2 pfam07676 PD40				0	N
	2265144529	POR3G_0192 7	FOG: WD40 repeat	COG2319 FOG: WD40 repeat	pfam00400 WD40				0	N
	2265144530	POR3G_0192 8	FOG: WD40 repeat	COG2319 FOG: WD40 repeat	pfam00400 WD40				0	N
	2265144531	POR3G_0192 9	FOG: WD40 repeat	COG2319 FOG: WD40 repeat	pfam00400 WD40				0	N

	2265144532	POR3G_0193 0	Protein kinase domain.		pfam00069 Pkinase		EC:2.7.11.1 Non-specific serine/threonine protein kinase.	KO:K08884 K08884 serine/threonine protein kinase, bacterial [EC:2.7.11.1]	0	N
	2265144534	POR3G_0193 2	FOG: WD40 repeat	COG2319 FOG: WD40 repeat	pfam00400 WD40				1	N
	2265144541	POR3G_0193 9	Trypsin-like serine proteases, typically periplasmic, contain C-terminal PDZ domain	COG0265 Trypsin-like serine proteases, typically periplasmic, contain C-terminal PDZ domain	pfam13414 TPR_11 pfam13365 Trypsin_2 pfam00515 TPR_1				0	N
	2265144549	POR3G_0194 7	hypothetical protein		pfam13485 Peptidase_MA_2 pfam13414 TPR_11				0	N
	2265144568	POR3G_0196 6	Tfp pilus assembly protein PilF	COG3063 Tfp pilus assembly protein PilF	pfam07719 TPR_2 pfam00515 TPR_1 pfam13414 TPR_11 pfam13676 TIR_2				0	N
	2265144570	POR3G_0196 8	Tfp pilus assembly protein PilF	COG3063 Tfp pilus assembly protein PilF	pfam13414 TPR_11 pfam00515 TPR_1				0	N
	2265144571	POR3G_0196 9	Flp pilus assembly protein TadD, contains TPR repeats	COG5010 Flp pilus assembly protein TadD,	pfam13676 TIR_2 pfam13414				0	N

				contains TPR repeats	TPR_11					
	2265144576	POR3G_0197 4	Gluconolactonase	COG3386 Gluconolactona se	pfam01436 NHL				0	N
	2265144603	POR3G_0200 1	FOG: WD40 repeat	COG2319 FOG: WD40 repeat	pfam13860 FlgD_ig pfam00400 WD40	TIGR04183 Por secretion system C-terminal sorting domain			1	Y
	2265144604	POR3G_0200 2	FOG: WD40 repeat	COG2319 FOG: WD40 repeat	pfam00963 Cohesin pfam00400 WD40	TIGR04183 Por secretion system C-terminal sorting domain			0	Y
	2265144605	POR3G_0200 3	FOG: WD40 repeat	COG2319 FOG: WD40 repeat	pfam00400 WD40	TIGR02608 delta-60 repeat domain			0	Y
	2265144606	POR3G_0200 4	FOG: WD40 repeat	COG2319 FOG: WD40 repeat	pfam13516 LRR_6 pfam00400 WD40 pfam12799 LRR_4 pfam13385 Laminin_G_3 pfam13860 FlgD_ig	TIGR04183 Por secretion system C-terminal sorting domain			0	Y
	2265144613	POR3G_0201 1	Spore coat assembly protein	COG5337 Spore coat assembly protein	pfam12796 Ank_2 pfam08757 CotH				0	Y
	2265144694	POR3G_0209 2	Tetratricopeptide repeat.		pfam13414 TPR_11				1	N

	2265144710	POR3G_02108	FOG: WD40 repeat	COG2319 FOG: WD40 repeat	pfam00400 WD40 pfam08662 eIF2A				0	N
	2265144736	POR3G_02134	FOG: Ankyrin repeat	COG0666 FOG: Ankyrin repeat	pfam00023 Ank pfam12796 Ank_2				0	N
	2265144758	POR3G_02156	hypothetical protein		pfam13205 Big_5				0	N
	2265144809	POR3G_02207	Fibronectin type III domain.		pfam00932 LTD pfam00041 fn3				1	N
	2265144884	POR3G_02282	Uncharacterized protein conserved in bacteria	COG1729 Uncharacterized protein conserved in bacteria	pfam09976 TPR_21 pfam13174 TPR_6 pfam13432 TPR_16				1	N
	2265144967	POR3G_02424	hypothetical protein	COG1520 FOG: WD40-like repeat	pfam13360 PQQ_2				3	N
	2265144971	POR3G_02428	Dockerin type I repeat.		pfam13205 Big_5 pfam00404 Dockerin_1				0	Y
	2265145047	POR3G_02504	FOG: WD40 repeat	COG2319 FOG: WD40 repeat	pfam00400 WD40				0	N
	2265145080	POR3G_02537	ASPIC and UnbV.		pfam07593 UnbV_ASPIC pfam13860 FlgD_ig pfam13517 VCBS	TIGR04183 Por secretion system C-terminal sorting domain			1	Y

	2265145083	POR3G_02540	FOG: WD40 repeat	COG2319 FOG: WD40 repeat	pfam00400 WD40				0	Y
	2265145095	POR3G_02552	hypothetical protein		pfam13517 VCBS				0	N
	2265145096	POR3G_02553	Flp pilus assembly protein TadD, contains TPR repeats	COG5010 Flp pilus assembly protein TadD, contains TPR repeats	pfam07719 TPR_2 pfam13414 TPR_11				1	N
	2265145098	POR3G_02555	FOG: WD40 repeat	COG2319 FOG: WD40 repeat	pfam00400 WD40 pfam04151 PPC	TIGR04183 Por secretion system C-terminal sorting domain			1	N
	2265145121	POR3G_02578	Tfp pilus assembly protein PilF	COG3063 Tfp pilus assembly protein PilF	pfam13414 TPR_11 pfam13181 TPR_8 pfam07719 TPR_2				0	N
	2265145187	POR3G_02644	Leucine-rich repeat (LRR) protein	COG4886 Leucine-rich repeat (LRR) protein	pfam12799 LRR_4				1	N
	2265145193	POR3G_02650	Lipoprotein NlpI, contains TPR repeats	COG4785 Lipoprotein NlpI, contains TPR repeats	pfam13414 TPR_11				0	N
	2265145204	POR3G_02661	FOG: Ankyrin repeat	COG0666 FOG: Ankyrin repeat	pfam12796 Ank_2				0	N
	2265145237	POR3G_02694	hypothetical protein	COG3063 Tfp pilus assembly protein PilF	pfam13414 TPR_11				0	N

	2265145239	POR3G_0269 6	Trypsin-like serine proteases, typically periplasmic, contain C-terminal PDZ domain	COG0265 Trypsin-like serine proteases, typically periplasmic, contain C-terminal PDZ domain	pfam13414 TPR_11 pfam00515 TPR_1 pfam13365 Trypsin_2				1	Y
	2265145240	POR3G_0269 7	Trypsin-like serine proteases, typically periplasmic, contain C-terminal PDZ domain	COG0265 Trypsin-like serine proteases, typically periplasmic, contain C-terminal PDZ domain	pfam13414 TPR_11 pfam13365 Trypsin_2				0	N
	2265145400	POR3G_0285 8	ASPIC and UnbV.		pfam13517 VCBS pfam07593 UnbV_ASPIC				0	N
	2265145401	POR3G_0285 9	Tfp pilus assembly protein PilF	COG3063 Tfp pilus assembly protein PilF	pfam00515 TPR_1 pfam13414 TPR_11				0	Y
	2265145409	POR3G_0286 7	Putative Zn-dependent protease, contains TPR repeats	COG4783 Putative Zn-dependent protease, contains TPR repeats	pfam08867 FRG pfam13414 TPR_11 pfam12950 TaqI_C				0	N
	2265145411	POR3G_0286 9	Tfp pilus assembly protein PilF	COG3063 Tfp pilus assembly protein PilF	pfam00515 TPR_1 pfam13414 TPR_11 pfam13181				0	N

					TPR_8					
	2265145415	POR3G_0287 3	Tfp pilus assembly protein PilF	COG3063 Tfp pilus assembly protein PilF	pfam13174 TPR_6 pfam13414 TPR_11				0	N
	2265145638	POR3G_0309 6	FOG: WD40-like repeat	COG1520 FOG: WD40-like repeat	pfam13360 PQQ_2				1	N
	2265145640	POR3G_0309 8	FOG: WD40-like repeat	COG1520 FOG: WD40-like repeat	pfam13360 PQQ_2				1	Y
	2265145672	POR3G_0313 0	Leucine-rich repeat (LRR) protein	COG4886 Leucine-rich repeat (LRR) protein	pfam07635 PSCyt1 pfam12799 LRR_4 pfam00963 Cohesin				1	N
	2265145674	POR3G_0313 2	RNA polymerase sigma factor, sigma-70 family	COG1595 DNA-directed RNA polymerase specialized sigma subunit, sigma24 homolog	pfam08281 Sigma70_r4_2 pfam04542 Sigma70_r2 pfam12799 LRR_4	TIGR02937 RNA polymerase sigma factor, sigma-70 family			0	N
	2265145675	POR3G_0313 3	Leucine-rich repeat (LRR) protein	COG4886 Leucine-rich repeat (LRR) protein	pfam12799 LRR_4 pfam00963 Cohesin pfam07635 PSCyt1	TIGR04183 Por secretion system C-terminal sorting domain			0	Y
	2265145676	POR3G_0313 4	Leucine-rich repeat (LRR) protein	COG4886 Leucine-rich repeat (LRR) protein	pfam12799 LRR_4				0	N

	2265145677	POR3G_0313 5	Leucine-rich repeat (LRR) protein	COG4886 Leucine-rich repeat (LRR) protein	pfam12799 LRR_4				1	N
	2265145678	POR3G_0313 6	Leucine-rich repeat (LRR) protein	COG4886 Leucine-rich repeat (LRR) protein	pfam12799 LRR_4				0	N
	2265145679	POR3G_0313 7	Leucine-rich repeat (LRR) protein	COG4886 Leucine-rich repeat (LRR) protein	pfam03537 Glyco_hydro_11 4 pfam12799 LRR_4				1	N
	2265145680	POR3G_0313 8	Leucine-rich repeat (LRR) protein	COG4886 Leucine-rich repeat (LRR) protein	pfam12799 LRR_4				0	N
	2265145705	POR3G_0316 3	Glucose/sorbosone dehydrogenases	COG2133 Glucose/sorbos one dehydrogenase s	pfam00058 Ldl_recept_b pfam13860 FlgD_ig	TIGR04183 Por secretion system C-terminal sorting domain			0	Y
	2265145707	POR3G_0316 5	Leucine-rich repeat (LRR) protein	COG4886 Leucine-rich repeat (LRR) protein	pfam12799 LRR_4 pfam13688 Reprolysin_5 pfam13860 FlgD_ig	TIGR04183 Por secretion system C-terminal sorting domain			0	Y
	2265145708	POR3G_0316 6	ABC-type branched-chain amino acid transport systems, periplasmic component	COG0683 ABC-type branched-chain amino acid transport systems, periplasmic component	pfam00404 Dockerin_1 pfam13458 Peripla_BP_6				0	N

	2265145709	POR3G_0316 7	Leucine-rich repeat (LRR) protein	COG4886 Leucine-rich repeat (LRR) protein	pfam12799 LRR_4 pfam13860 FlgD_ig	TIGR04183 Por secretion system C-terminal sorting domain			1	Y
	2265145710	POR3G_0316 8	Leucine-rich repeat (LRR) protein	COG4886 Leucine-rich repeat (LRR) protein	pfam12799 LRR_4 pfam12044 Metallopep				1	Y
	2265145711	POR3G_0316 9	FOG: WD40 repeat	COG2319 FOG: WD40 repeat	pfam13860 FlgD_ig pfam00400 WD40	TIGR04183 Por secretion system C-terminal sorting domain			0	Y
	2265145712	POR3G_0317 0	FOG: WD40 repeat	COG2319 FOG: WD40 repeat	pfam00400 WD40 pfam13860 FlgD_ig	TIGR04183 Por secretion system C-terminal sorting domain			0	N
	2265145719	POR3G_0317 7	FOG: WD40 repeat	COG2319 FOG: WD40 repeat	pfam07676 PD40 pfam00400 WD40				0	Y
	2265145723	POR3G_0318 1	Uncharacterized conserved protein	COG3391 Uncharacterized conserved protein	pfam13860 FlgD_ig pfam00058 Ldl_recept_b	TIGR04183 Por secretion system C-terminal sorting domain			1	N
	2265145724	POR3G_0318 2	Low-density lipoprotein receptor repeat class B.	COG3391 Uncharacterized conserved protein	pfam13860 FlgD_ig pfam00058 Ldl_recept_b	TIGR04183 Por secretion system C-terminal sorting domain			1	N
	2265145745	POR3G_0320	Zn-dependent hydrolases, including	COG0491 Zn-dependent	pfam07719 TPR_2				0	N

		3	glyoxylases	hydrolases, including glyoxylases	pfam00753 Lactamase_B					
	2265145750	POR3G_03208	Periplasmic protein TonB, links inner and outer membranes	COG0810 Periplasmic protein TonB, links inner and outer membranes	pfam13750 Big_3_3				1	Y
	2265145762	POR3G_03220	FOG: TPR repeat	COG0457 FOG: TPR repeat	pfam12969 DUF3857 pfam01841 Transglut_core pfam07719 TPR_2 pfam13432 TPR_16				1	N
	2265145786	POR3G_03244	FOG: WD40 repeat	COG2319 FOG: WD40 repeat	pfam00400 WD40				1	N
	2265145787	POR3G_03245	FOG: WD40 repeat	COG2319 FOG: WD40 repeat	pfam00400 WD40				0	N
	2265145788	POR3G_03246	FOG: WD40 repeat	COG2319 FOG: WD40 repeat	pfam00400 WD40 pfam08662 eIF2A				0	Y
	2265145843	POR3G_03301	Tfp pilus assembly protein PilF	COG3063 Tfp pilus assembly protein PilF	pfam00515 TPR_1 pfam13414 TPR_11 pfam13174 TPR_6				0	N
	2265145848	POR3G_03306	FOG: WD40 repeat	COG2319 FOG: WD40 repeat	pfam00400 WD40				0	N

	2265145849	POR3G_03307	FOG: WD40 repeat	COG2319 FOG: WD40 repeat	pfam00400 WD40					0	N
	2265145912	POR3G_03370	Tfp pilus assembly protein PilF	COG3063 Tfp pilus assembly protein PilF	pfam13432 TPR_16 pfam13429 TPR_15 pfam13414 TPR_11 pfam13431 TPR_17					1	N
	2265145913	POR3G_03371	Tetratricopeptide repeat.	COG2956 Predicted N-acetylglucosaminyl transferase	pfam13414 TPR_11 pfam13432 TPR_16 pfam13385 Laminin_G_3 pfam00515 TPR_1					1	N
	2265145937	POR3G_03395	Tfp pilus assembly protein PilF	COG3063 Tfp pilus assembly protein PilF	pfam13414 TPR_11 pfam00515 TPR_1					0	N
	2265145959	POR3G_03417	Tfp pilus assembly protein PilF	COG3063 Tfp pilus assembly protein PilF	pfam13676 TIR_2 pfam13414 TPR_11					0	N
	2265145965	POR3G_03423	Flp pilus assembly protein TadD, contains TPR repeats	COG5010 Flp pilus assembly protein TadD, contains TPR repeats	pfam13414 TPR_11 pfam00515 TPR_1 pfam08867 FRG					0	N
	2265145967	POR3G_03425	Lipoprotein NlpI, contains TPR repeats	COG4785 Lipoprotein NlpI, contains TPR	pfam13414 TPR_11					0	N

				repeats						
	2265146104	POR3G_0356 2	FOG: WD40 repeat	COG2319 FOG: WD40 repeat	pfam00400 WD40				0	N
	2265146124	POR3G_0358 2	Flp pilus assembly protein TadD, contains TPR repeats	COG5010 Flp pilus assembly protein TadD, contains TPR repeats	pfam13414 TPR_11 pfam13176 TPR_7 pfam13432 TPR_16				0	N
	2265146178	POR3G_0363 6	Lipoprotein NlpI, contains TPR repeats	COG4785 Lipoprotein NlpI, contains TPR repeats	pfam13414 TPR_11				0	N
	2265146187	POR3G_0364 5	Tfp pilus assembly protein PilF	COG3063 Tfp pilus assembly protein PilF	pfam00515 TPR_1 pfam13414 TPR_11				0	N
	2265146191	POR3G_0364 9	FOG: WD40 repeat	COG2319 FOG: WD40 repeat	pfam13860 FlgD_ig pfam00400 WD40 pfam05345 He_PIG	TIGR04183 Por secretion system C-terminal sorting domain			1	N
	2265146230	POR3G_0368 8	FOG: WD40-like repeat	COG1520 FOG: WD40-like repeat	pfam13360 PQQ_2				0	N
	2265146232	POR3G_0369 0	FOG: WD40-like repeat	COG1520 FOG: WD40-like repeat	pfam13360 PQQ_2				1	Y
	2265146255	POR3G_0371 3	FOG: WD40 repeat	COG2319 FOG: WD40 repeat	pfam00400 WD40				0	N
	2265146280	POR3G_0373	Tfp pilus assembly protein PilF	COG3063 Tfp pilus assembly	pfam13414 TPR_11				0	N

		8		protein PilF	pfam03683 UPF0175					
	2265146285	POR3G_0374 3	Uncharacterized protein conserved in bacteria	COG1729 Uncharacterized protein conserved in bacteria	pfam13414 TPR_11				1	N
	2265146331	POR3G_0378 9	Uncharacterized protein conserved in bacteria	COG1729 Uncharacterized protein conserved in bacteria	pfam13174 TPR_6				1	N
	2265146370	POR3G_0382 8	Predicted Zn-dependent protease	COG5549 Predicted Zn-dependent protease	pfam00413 Peptidase_M10 pfam13181 TPR_8 pfam13424 TPR_12 pfam13414 TPR_11				1	N
	2265146393	POR3G_0385 1	FOG: WD40 repeat	COG2319 FOG: WD40 repeat	pfam00400 WD40 pfam12044 Metallopep	TIGR04183 Por secretion system C-terminal sorting domain			1	Y
	2265146424	POR3G_0388 2	Predicted spermidine synthase with an N-terminal membrane domain	COG4262 Predicted spermidine synthase with an N-terminal membrane domain	pfam01564 Spermine_synth pfam13414 TPR_11 pfam13424 TPR_12		EC:2.5.1.16 Spermidine synthase.	KO:K00797 speE, SRM spermidine synthase [EC:2.5.1.16]	13	N
	2265146429	POR3G_0390 9	FOG: WD40 repeat	COG2319 FOG: WD40 repeat	pfam00400 WD40				0	N

	2265146482	POR3G_03982	FOG: WD40 repeat	COG2319 FOG: WD40 repeat	pfam00400 WD40				0	Y
	2265146514	POR3G_04014	FOG: WD40 repeat	COG2319 FOG: WD40 repeat	pfam00400 WD40				0	N
	2265146522	POR3G_04022	RNA polymerase sigma factor, sigma-70 family	COG2319 FOG: WD40 repeat	pfam08281 Sigma70_r4_2 pfam00400 WD40 pfam04542 Sigma70_r2	TIGR02937 RNA polymerase sigma factor, sigma-70 family			0	N
	2265146524	POR3G_04024	Uncharacterized conserved protein	COG3391 Uncharacterized conserved protein	pfam01436 NHL				0	N
	2265146576	POR3G_04076	Dockerin type I repeat.		pfam13860 FlgD_ig pfam00404 Dockerin_1	TIGR04183 Por secretion system C-terminal sorting domain			0	N
	2265146578	POR3G_04078	Leucine-rich repeat (LRR) protein	COG4886 Leucine-rich repeat (LRR) protein	pfam00963 Cohesin pfam12799 LRR_4 pfam13860 FlgD_ig	TIGR04183 Por secretion system C-terminal sorting domain			0	Y
	2265146581	POR3G_04081	Leucine-rich repeat (LRR) protein	COG4886 Leucine-rich repeat (LRR) protein	pfam07635 PSCyt1 pfam12799 LRR_4 pfam00963 Cohesin pfam13860 FlgD_ig	TIGR04183 Por secretion system C-terminal sorting domain			1	Y
	2265146594	POR3G_0409	RNA polymerase sigma factor,	COG1595 DNA-directed RNA	pfam04542 Sigma70_r2	TIGR02937 RNA			0	N

		4	sigma-70 family	polymerase specialized sigma subunit, sigma24 homolog	pfam08281 Sigma70_r4_2 pfam00400 WD40	polymerase sigma factor, sigma-70 family				
	2265146597	POR3G_0409 7	Protein kinase domain./Tetratricopeptide repeat.	COG1196 Chromosome segregation ATPases	pfam00069 Pkinase pfam13414 TPR_11				1	N
	2265146601	POR3G_0410 1	Tfp pilus assembly protein PilF	COG3063 Tfp pilus assembly protein PilF	pfam13414 TPR_11				1	N
	2265146706	POR3G_0423 1	Trypsin-like serine proteases, typically periplasmic, contain C-terminal PDZ domain	COG0265 Trypsin-like serine proteases, typically periplasmic, contain C-terminal PDZ domain	pfam13414 TPR_11 pfam13365 Trypsin_2				1	N
	2265146709	POR3G_0423 4	Flp pilus assembly protein TadD, contains TPR repeats	COG5010 Flp pilus assembly protein TadD, contains TPR repeats	pfam13414 TPR_11				0	N
	2265146726	POR3G_0425 1	Leucine-rich repeat (LRR) protein	COG4886 Leucine-rich repeat (LRR) protein	pfam13860 FlgD_ig pfam12799 LRR_4	TIGR04183 Por secretion system C-terminal sorting domain			1	N
	2265146727	POR3G_0425 2	Low-density lipoprotein receptor repeat class B./CARDB./Trypsin.	COG3391 Uncharacterized conserved protein	pfam00058 Ldl_recept_b pfam07705 CARDB pfam13365	TIGR04183 Por secretion system C-terminal sorting			1	N

					Trypsin_2 pfam13860 FlgD_ig	domain				
	2265146737	POR3G_04262	NHL repeat.	COG4257 Streptogramin lyase	pfam01436 NHL				0	N
	2265146755	POR3G_04307	FOG: WD40 repeat	COG2319 FOG: WD40 repeat	pfam00400 WD40				1	N
	2265146756	POR3G_04308	FOG: WD40 repeat	COG2319 FOG: WD40 repeat	pfam00400 WD40				0	N
	2265146783	POR3G_04335	Cytochrome c biogenesis factor	COG4235 Cytochrome c biogenesis factor	pfam13414 TPR_11 pfam13517 VCBS pfam13181 TPR_8				0	Y
	2265146808	POR3G_04360	hypothetical protein		pfam13414 TPR_11				0	N
	2265146827	POR3G_04379	FOG: WD40 repeat	COG2319 FOG: WD40 repeat	pfam00963 Cohesin pfam00400 WD40	TIGR04183 Por secretion system C-terminal sorting domain			1	N
	2265146838	POR3G_04390	Uncharacterized conserved protein	COG1262 Uncharacterized conserved protein	pfam13365 Trypsin_2 pfam13414 TPR_11 pfam03781 FGE-sulfatase				1	N
	2265146839	POR3G_04391	hypothetical protein	COG0265 Trypsin-like serine proteases, typically	pfam13414 TPR_11				0	N

				periplasmic, contain C-terminal PDZ domain							
	2265146840	POR3G_04392	Trypsin-like serine proteases, typically periplasmic, contain C-terminal PDZ domain	COG0265 Trypsin-like serine proteases, typically periplasmic, contain C-terminal PDZ domain	pfam13414 TPR_11					0	N
	2265146848	POR3G_04400	Leucine-rich repeat (LRR) protein	COG4886 Leucine-rich repeat (LRR) protein	pfam00932 LTD pfam12799 LRR_4 pfam00028 Cadherin					0	Y
	2265146854	POR3G_04406	Gluconolactonase	COG3386 Gluconolactonase	pfam01436 NHL					1	N
	2265146875	POR3G_04427	Leucine-rich repeat (LRR) protein	COG4886 Leucine-rich repeat (LRR) protein	pfam12799 LRR_4 pfam00963 Cohesin	TIGR04183 Por secretion system C-terminal sorting domain				0	N
	2265146884	POR3G_04436	FOG: Ankyrin repeat	COG0666 FOG: Ankyrin repeat	pfam12796 Ank_2					0	N
	2265146899	POR3G_04451	FOG: Ankyrin repeat	COG0666 FOG: Ankyrin repeat	pfam13517 VCBS pfam07593 UnbV_ASPIC pfam12796 Ank_2					1	N

	2265146901	POR3G_04453	Tetratricopeptide repeat.	COG4783 Putative Zn-dependent protease, contains TPR repeats	pfam00515 TPR_1 pfam13414 TPR_11					0	N
	2265146931	POR3G_04491	Predicted RNA-binding protein homologous to eukaryotic snRNP	COG1293 Predicted RNA-binding protein homologous to eukaryotic snRNP	pfam05670 DUF814 pfam05833 FbpA					0	N
	2265146937	POR3G_04497	Tfp pilus assembly protein PilF	COG3063 Tfp pilus assembly protein PilF	pfam13414 TPR_11 pfam00515 TPR_1					0	N
	2265146960	POR3G_04527	FOG: WD40 repeat	COG2319 FOG: WD40 repeat	pfam13365 Trypsin_2 pfam00930 DPPIV_N pfam00400 WD40					1	N
	2265146961	POR3G_04528	Putative Zn-dependent protease, contains TPR repeats	COG4783 Putative Zn-dependent protease, contains TPR repeats	pfam13174 TPR_6 pfam13414 TPR_11 pfam07719 TPR_2					1	N
	2265146962	POR3G_04529	Uncharacterized protein conserved in bacteria	COG1729 Uncharacterized protein conserved in bacteria	pfam13432 TPR_16 pfam07719 TPR_2 pfam13414 TPR_11					1	N
	2265146970	POR3G_0453	hypothetical protein	COG1729 Uncharacterized	pfam13414 TPR_11					0	N

		7		protein conserved in bacteria	pfam07719 TPR_2					
	2265147034	POR3G_0460 1	Uncharacterized conserved protein	COG2912 Uncharacterized conserved protein	pfam13371 TPR_9				0	N
	2265147040	POR3G_0460 7	Tfp pilus assembly protein PilF	COG3063 Tfp pilus assembly protein PilF	pfam13414 TPR_11 pfam03683 UPF0175				0	N
	2265147043	POR3G_0461 0	Tfp pilus assembly protein PilF	COG3063 Tfp pilus assembly protein PilF	pfam13414 TPR_11 pfam13181 TPR_8				0	Y
	2265147054	POR3G_0462 1	Trypsin-like serine proteases, typically periplasmic, contain C-terminal PDZ domain	COG0265 Trypsin-like serine proteases, typically periplasmic, contain C-terminal PDZ domain	pfam13365 Trypsin_2 pfam13414 TPR_11				0	Y
	2265147063	POR3G_0463 0	Uncharacterized protein conserved in bacteria	COG1729 Uncharacterized protein conserved in bacteria	pfam13174 TPR_6				0	Y
	2265147082	POR3G_0465 4	Leucine-rich repeat (LRR) protein	COG4886 Leucine-rich repeat (LRR) protein	pfam12799 LRR_4				0	N
	2265147083	POR3G_0465	Leucine-rich repeat (LRR) protein	COG4886 Leucine-rich	pfam12799			KO:K13730 inlA	1	N

		5		repeat (LRR) protein	LRR_4			internalin A		
	2265147089	POR3G_0466 1	Tfp pilus assembly protein PilF	COG3063 Tfp pilus assembly protein PilF	pfam13414 TPR_11				0	N
	2265147102	POR3G_0467 4	Flp pilus assembly protein TadD, contains TPR repeats	COG5010 Flp pilus assembly protein TadD, contains TPR repeats	pfam00515 TPR_1 pfam13414 TPR_11 pfam07719 TPR_2			KO:K12600 SKI3, TTC37 superkiller protein 3	0	N
	2265147136	POR3G_0470 8	Cadherin domain.		pfam00028 Cadherin				1	Y
	2265147152	POR3G_0472 4	Leucine-rich repeat (LRR) protein	COG4886 Leucine-rich repeat (LRR) protein	pfam12799 LRR_4				0	Y
	2265147191	POR3G_0476 3	Predicted N-acetylglucosaminyl transferase	COG2956 Predicted N-acetylglucosaminyl transferase	pfam13414 TPR_11				1	N
	2265147201	POR3G_0477 3	Leucine-rich repeat (LRR) protein	COG4886 Leucine-rich repeat (LRR) protein	pfam00404 Dockerin_1 pfam12799 LRR_4	TIGR04183 Por secretion system C-terminal sorting domain			1	N
	2265147217	POR3G_0478 9	FOG: WD40 repeat	COG2319 FOG: WD40 repeat	pfam00400 WD40				0	N
	2265147241	POR3G_0481 3	Lipoprotein NlpI, contains TPR repeats	COG4785 Lipoprotein NlpI, contains TPR repeats	pfam13414 TPR_11 pfam07719 TPR_2				0	N

	2265147251	POR3G_04828	FOG: WD40 repeat	COG2319 FOG: WD40 repeat	pfam00400 WD40				0	N
	2265147265	POR3G_04844	ASPIC and UnbV.		pfam07593 UnbV_ASPIC pfam13517 VCBS				1	Y
	2265147281	POR3G_04861	Tfp pilus assembly protein PilF	COG3063 Tfp pilus assembly protein PilF	pfam13414 TPR_11				0	N
	2265147285	POR3G_04865	Flp pilus assembly protein TadD, contains TPR repeats	COG5010 Flp pilus assembly protein TadD, contains TPR repeats	pfam00515 TPR_1 pfam13414 TPR_11 pfam13181 TPR_8				0	N
	2265147298	POR3G_04878	FOG: WD40 repeat	COG2319 FOG: WD40 repeat	pfam00400 WD40				0	N
	2265147302	POR3G_04882	Cytochrome c biogenesis factor	COG4235 Cytochrome c biogenesis factor	pfam13424 TPR_12 pfam07719 TPR_2 pfam13414 TPR_11				0	N
	2265147318	POR3G_04898	Low-density lipoprotein receptor repeat class B.	COG3391 Uncharacterized conserved protein	pfam00058 Ldl_recept_b				1	N
	2265147321	POR3G_04901	Leucine-rich repeat (LRR) protein	COG4886 Leucine-rich repeat (LRR) protein	pfam12799 LRR_4				0	N
	2265147323	POR3G_04903	FOG: WD40 repeat	COG2319 FOG: WD40 repeat	pfam00400 WD40				1	Y

	2265147368	POR3G_04950	Fibronectin type III domain.	COG3401 Fibronectin type 3 domain-containing protein	pfam00041 fn3				0	N
	2265140811	or0807	FOG: WD40 repeat	COG2319 FOG: WD40 repeat	pfam00400 WD40				0	N
	2265140812	or0808	FOG: WD40 repeat	COG2319 FOG: WD40 repeat	pfam00400 WD40				0	N
	2265140825	or0137	Leucine-rich repeat (LRR) protein	COG4886 Leucine-rich repeat (LRR) protein	pfam00963 Cohesin pfam12799 LRR_4 pfam13860 FlgD_ig	TIGR04183 Por secretion system C-terminal sorting domain			0	N
	2265140848	or0850	Tfp pilus assembly protein PilF	COG3063 Tfp pilus assembly protein PilF	pfam07719 TPR_2 pfam13414 TPR_11				1	N
4C	2265140865	or0871	Low-density lipoprotein receptor repeat class B.	COG3391 Uncharacterized conserved protein	pfam00058 Ldl_recept_b	TIGR04183 Por secretion system C-terminal sorting domain			1	N
	2265140866	or0873	FOG: WD40 repeat	COG2319 FOG: WD40 repeat	pfam00400 WD40				0	N
	2265140867	or0874	FOG: WD40 repeat	COG2319 FOG: WD40 repeat	pfam00400 WD40				0	N
	2265140898	or0904	FOG: WD40 repeat	COG2319 FOG: WD40 repeat	pfam00400 WD40				0	N
	2265140901	or0907	Cadherin domain.		pfam00932 LTD pfam13860 FlgD_ig	TIGR04183 Por secretion system C-			1	N

					pfam00028 Cadherin	terminal sorting domain				
	2265140959	or0974	Large extracellular alpha-helical protein	COG2373 Large extracellular alpha-helical protein	pfam01835 A2M_N pfam10569 Thiol-ester_cl pfam07703 A2M_N_2 pfam11974 MG1 pfam13205 Big_5 pfam00207 A2M			KO:K06894 K06894	1	N
	2265141061	or0173	Leucine-rich repeat (LRR) protein	COG4886 Leucine-rich repeat (LRR) protein	pfam12799 LRR_4				1	N
	2265141062	or0174	Leucine-rich repeat (LRR) protein	COG4886 Leucine-rich repeat (LRR) protein	pfam12799 LRR_4				0	N
	2265141147	or1172	Uncharacterized protein conserved in bacteria	COG1729 Uncharacterized protein conserved in bacteria	pfam13174 TPR_6				0	N
	2265141181	or0026	Tetratricopeptide repeat.	COG3063 Tfp pilus assembly protein PilF	pfam07719 TPR_2 pfam13414 TPR_11				0	N
	2265141254	or1235	hypothetical protein		pfam13517 VCBS				0	N
	2265141303	or1295	FOG: WD40 repeat	COG2319 FOG:	pfam00400 WD40				1	N

				WD40 repeat	pfam07676 PD40					
	2265141304	or1296	FOG: WD40 repeat	COG2319 FOG: WD40 repeat	pfam00400 WD40				1	N
	2265141305	or1297	FOG: WD40 repeat	COG2319 FOG: WD40 repeat	pfam00400 WD40				1	N
	2265141397	or1390	FOG: WD40 repeat	COG2319 FOG: WD40 repeat	pfam00400 WD40				0	N
	2265141413	or1407	Tetratricopeptide repeat.	COG4783 Putative Zn-dependent protease, contains TPR repeats	pfam13414 TPR_11				0	N
	2265141418	or1412	WD-40 repeat-containing protein	COG2319 FOG: WD40 repeat	pfam00400 WD40				0	N
	2265141425	or0235	Spermidine synthase	COG0421 Spermidine synthase	pfam13414 TPR_11 pfam07690 MFS_1 pfam01564 Spermine_synth		EC:2.5.1.16 Spermidine synthase.	KO:K00797 speE, SRM spermidine synthase [EC:2.5.1.16]	13	N
	2265141448	or1427	RNA polymerase sigma factor, sigma-70 family	COG1595 DNA-directed RNA polymerase specialized sigma subunit, sigma24 homolog	pfam00400 WD40 pfam04542 Sigma70_r2 pfam08281 Sigma70_r4_2 pfam13360 PQQ_2	TIGR02937 RNA polymerase sigma factor, sigma-70 family			0	N
	2265141493	or1477	ASPIC and UnbV.		pfam07593 UnbV_ASPIC pfam13517				1	Y

					VCBS						
	2265141504	or1488	hypothetical protein		pfam13414 TPR_11				1	Y
	2265141514	or1498	FOG: WD40 repeat	COG2319 FOG: WD40 repeat	pfam00400 WD40				0	N
	2265141523	or1508	FOG: WD40 repeat	COG2319 FOG: WD40 repeat	pfam00400 WD40				0	N
	2265141532	or1517	IPT/TIG domain.		pfam13205 Big_5 pfam01833 TIG				1	N
	2265141534	or1519	Tetratricopeptide repeat.	COG5010 Flp pilus assembly protein TadD, contains TPR repeats	pfam13414 TPR_11 pfam13424 TPR_12 pfam13432 TPR_16				0	N
	2265141556	or1542	Tetratricopeptide repeat./FRG domain.	COG3063 Tfp pilus assembly protein PilF	pfam13414 TPR_11 pfam08867 FRG pfam00515 TPR_1				0	N
	2265141557	or1543	Tetratricopeptide repeat.	COG3063 Tfp pilus assembly protein PilF	pfam13414 TPR_11 pfam12950 TaqI_C pfam00515 TPR_1				0	N
	2265141559	or1546	FOG: WD40 repeat	COG2319 FOG: WD40 repeat	pfam00400 WD40				0	N
	2265141635	or1600	hypothetical protein		pfam13517 VCBS				0	N

	2265141649	or1616	NHL repeat.	COG3391 Uncharacterized conserved protein	pfam01436 NHL				0	N
	2265141682	or1653	FOG: WD40 repeat	COG2319 FOG: WD40 repeat	pfam00400 WD40				1	N
	2265141683	or1654	FOG: WD40 repeat	COG2319 FOG: WD40 repeat	pfam00400 WD40				0	Y
	2265141696	or1669	FOG: WD40 repeat	COG2319 FOG: WD40 repeat	pfam00400 WD40	TIGR04183 Por secretion system C-terminal sorting domain			0	N
	2265141785	or1764	FOG: WD40 repeat	COG2319 FOG: WD40 repeat	pfam00400 WD40 pfam12044 Metallopep				1	N
	2265141793	or1774	Predicted RNA-binding protein homologous to eukaryotic snRNP	COG1293 Predicted RNA-binding protein homologous to eukaryotic snRNP	pfam05833 FbpA pfam05670 DUF814				0	N
	2265141819	or1800	FOG: WD40 repeat	COG2319 FOG: WD40 repeat	pfam00400 WD40				1	N
	2265141832	or1814	Putative Zn-dependent protease, contains TPR repeats	COG4783 Putative Zn-dependent protease, contains TPR repeats	pfam13181 TPR_8 pfam13414 TPR_11				1	N
	2265141848	or1830	FOG: WD40 repeat	COG2319 FOG: WD40 repeat	pfam00400 WD40				0	N

	2265141849	or1832	FOG: WD40 repeat	COG2319 FOG: WD40 repeat	pfam00400 WD40					1	N
	2265141858	or1842	Tetratricopeptide repeat./FRG domain.	COG4783 Putative Zn-dependent protease, contains TPR repeats	pfam13414 TPR_11 pfam08867 FRG					0	N
	2265141868	or1854	Tetratricopeptide repeat.	COG5010 Flp pilus assembly protein TadD, contains TPR repeats	pfam00515 TPR_1 pfam07719 TPR_2 pfam13414 TPR_11					1	N
	2265141898	or1888	FOG: WD40 repeat	COG2319 FOG: WD40 repeat	pfam12894 Apc4_WD40 pfam00400 WD40					1	Y
	2265141899	or1889	FOG: WD40 repeat	COG2319 FOG: WD40 repeat	pfam00400 WD40					0	N
	2265141900	or1891	FOG: WD40 repeat	COG2319 FOG: WD40 repeat	pfam00963 Cohesin pfam13860 FlgD_ig pfam00400 WD40	TIGR04183 Por secretion system C-terminal sorting domain				0	N
	2265141908	or1902	FOG: WD40 repeat	COG2319 FOG: WD40 repeat	pfam00400 WD40					1	Y
	2265141936	or1933	Tfp pilus assembly protein PilF	COG3063 Tfp pilus assembly protein PilF	pfam13485 Peptidase_MA_2 pfam13414 TPR_11					0	N
	2265141943	or1940	FOG: WD40 repeat	COG2319 FOG:	pfam00400					0	N

				WD40 repeat	WD40						
	2265141955	or1954	Trypsin-like serine proteases, typically periplasmic, contain C-terminal PDZ domain	COG0265 Trypsin-like serine proteases, typically periplasmic, contain C-terminal PDZ domain	pfam13181 TPR_8 pfam13365 Trypsin_2 pfam13414 TPR_11				1	Y	
	2265142021	or0346	Trypsin-like serine proteases, typically periplasmic, contain C-terminal PDZ domain	COG0265 Trypsin-like serine proteases, typically periplasmic, contain C-terminal PDZ domain	pfam13365 Trypsin_2 pfam13414 TPR_11				1	N	
	2265142022	or0347	Trypsin-like serine proteases, typically periplasmic, contain C-terminal PDZ domain	COG0265 Trypsin-like serine proteases, typically periplasmic, contain C-terminal PDZ domain	pfam13414 TPR_11 pfam13365 Trypsin_2				0	N	
	2265142066	or0388	Leucine Rich Repeat./Cadherin domain.	COG4886 Leucine-rich repeat (LRR) protein	pfam00028 Cadherin pfam12799 LRR_4				1	N	
	2265142067	or0389	Leucine Rich Repeat./Cadherin domain.	COG4886 Leucine-rich repeat (LRR) protein	pfam00028 Cadherin pfam13516 LRR_6 pfam12799				1	N	

					LRR_4					
	2265142081	or0405	FOG: WD40 repeat	COG2319 FOG: WD40 repeat	pfam00400 WD40				1	N
	2265142084	or0408	Leucine-rich repeat (LRR) protein	COG4886 Leucine-rich repeat (LRR) protein	pfam00963 Cohesin pfam07635 PSCyt1 pfam12799 LRR_4	TIGR04183 Por secretion system C-terminal sorting domain			1	Y
	2265142085	or0409	Leucine Rich Repeat./Dockerin type I repeat.	COG4886 Leucine-rich repeat (LRR) protein	pfam00404 Dockerin_1 pfam13860 FlgD_ig pfam12799 LRR_4	TIGR04183 Por secretion system C-terminal sorting domain			1	N
	2265142087	or0412	FOG: WD40 repeat	COG2319 FOG: WD40 repeat	pfam00400 WD40				0	N
	2265142101	or0429	FOG: WD40 repeat	COG2319 FOG: WD40 repeat	pfam00400 WD40 pfam01551 Peptidase_M23				1	N
	2265142102	or0431	FOG: WD40 repeat	COG2319 FOG: WD40 repeat	pfam00400 WD40	TIGR04183 Por secretion system C-terminal sorting domain			0	N
	2265142116	or0443	Periplasmic protein TonB, links inner and outer membranes	COG0810 Periplasmic protein TonB, links inner and outer membranes	pfam12799 LRR_4				0	Y
	2265142134	or0463	Arylsulfatase A and related enzymes	COG3119 Arylsulfatase A	pfam00884 Sulfatase	TIGR04183 Por secretion			0	N

				and related enzymes	pfam00404 Dockerin_1	system C-terminal sorting domain				
	2265142146	or0476	ASPIC and UnbV.		pfam07593 UnbV_ASPIC pfam13517 VCBS	TIGR04183 Por secretion system C-terminal sorting domain			0	N
	2265142171	or0504	FOG: Ankyrin repeat	COG0666 FOG: Ankyrin repeat	pfam13857 Ank_5 pfam12796 Ank_2				0	N
	2265142186	or0093	Trypsin-like serine proteases, typically periplasmic, contain C-terminal PDZ domain	COG0265 Trypsin-like serine proteases, typically periplasmic, contain C-terminal PDZ domain	pfam13365 Trypsin_2 pfam00400 WD40				0	N
	2265142188	or0095	FOG: WD40 repeat	COG2319 FOG: WD40 repeat	pfam08662 eIF2A pfam11715 Nup160 pfam00400 WD40				0	N
	2265142239	or0556	Uncharacterized protein conserved in bacteria	COG1729 Uncharacterized protein conserved in bacteria	pfam13174 TPR_6				1	Y
	2265142246	or0563	FOG: WD40-like repeat	COG1520 FOG: WD40-like repeat	pfam13360 PQQ_2 pfam13570				3	Y

					PQQ_3					
	2265142364	or0694	Bacterial Ig-like domain (group 1).		pfam02369 Big_1 pfam13860 FlgD_ig	TIGR04183 Por secretion system C-terminal sorting domain			0	N
	2265142391	or0127	Leucine Rich Repeat./Dockerin type I repeat.	COG4886 Leucine-rich repeat (LRR) protein	pfam00404 Dockerin_1 pfam12799 LRR_4				1	N
	2265142405	or0740	FOG: WD40 repeat	COG2319 FOG: WD40 repeat	pfam00400 WD40				0	N
	2265142406	or0741	FOG: WD40 repeat	COG2319 FOG: WD40 repeat	pfam00400 WD40				0	N
	2265142425	or0132	Tetratricopeptide repeat.	COG4785 Lipoprotein NlpI, contains TPR repeats	pfam13414 TPR_11				0	N
4CII	2265147986	POR4CII_00055	FOG: WD40 repeat	COG2319 FOG: WD40 repeat	pfam13860 FlgD_ig pfam00400 WD40	TIGR04183 Por secretion system C-terminal sorting domain			0	Y
	2265148035	POR4CII_00104	Gluconolactonase	COG3386 Gluconolactonase	pfam01436 NHL				1	N
	2265148046	POR4CII_00115	Soluble lytic murein transglycosylase and related regulatory proteins (some contain LysM/invasin domains)	COG0741 Soluble lytic murein transglycosylase and related regulatory proteins (some contain	pfam01464 SLT pfam13174 TPR_6 pfam13525 YfiO				0	N

				LysM/invasin domains)						
	2265148123	POR4CII_00192	FOG: WD40 repeat	COG2319 FOG: WD40 repeat	pfam00400 WD40				1	N
	2265148156	POR4CII_00226	Lipoprotein NlpI, contains TPR repeats	COG4785 Lipoprotein NlpI, contains TPR repeats	pfam13414 TPR_11				0	N
	2265148186	POR4CII_00256	Tfp pilus assembly protein PilF	COG3063 Tfp pilus assembly protein PilF	pfam13414 TPR_11 pfam00515 TPR_1 pfam07719 TPR_2				1	N
	2265148266	POR4CII_00336	FOG: WD40 repeat	COG2319 FOG: WD40 repeat	pfam00400 WD40 pfam13860 FlgD_ig	TIGR04183 Por secretion system C-terminal sorting domain			0	Y
	2265148276	POR4CII_00346	Dockerin type I repeat.		pfam11306 DUF3108 pfam00404 Dockerin_1				0	Y
	2265148298	POR4CII_00368	Tfp pilus assembly protein PilF	COG3063 Tfp pilus assembly protein PilF	pfam13414 TPR_11 pfam13181 TPR_8				0	Y
	2265148310	POR4CII_00380	Putative Zn-dependent protease, contains TPR repeats	COG4783 Putative Zn-dependent protease, contains TPR repeats	pfam00515 TPR_1 pfam13414 TPR_11				0	N

	2265148327	POR4CII_00397	Tetratricopeptide repeat.		pfam13414 TPR_11					0	N
	2265148336	POR4CII_00444	Tfp pilus assembly protein PilF	COG3063 Tfp pilus assembly protein PilF	pfam13424 TPR_12 pfam07719 TPR_2 pfam13414 TPR_11					0	N
	2265148358	POR4CII_00480	FOG: WD40 repeat	COG2319 FOG: WD40 repeat	pfam00400 WD40					1	N
	2265148365	POR4CII_00502	FOG: WD40 repeat	COG2319 FOG: WD40 repeat	pfam00400 WD40					0	N
	2265148395	POR4CII_00536	Trypsin-like serine proteases, typically periplasmic, contain C-terminal PDZ domain	COG0265 Trypsin-like serine proteases, typically periplasmic, contain C-terminal PDZ domain	pfam07719 TPR_2 pfam00515 TPR_1					0	N
	2265148408	POR4CII_00549	Tetratricopeptide repeat.		pfam13414 TPR_11 pfam13174 TPR_6					0	N
4E	2265137499	or1151	Leucine Rich Repeat.	COG4886 Leucine-rich repeat (LRR) protein	pfam12799 LRR_4					0	Y
	2265137511	or1165	Tetratricopeptide repeat.	COG4785 Lipoprotein NlpI, contains TPR repeats	pfam13676 TIR_2 pfam13414 TPR_11					0	N

	2265137512	or1167	Tetratricopeptide repeat.	COG2956 Predicted N-acetylglucosaminyl transferase	pfam00515 TPR_1 pfam13414 TPR_11				0	N
	2265137513	or1168	Tetratricopeptide repeat.	COG5010 Flp pilus assembly protein TadD, contains TPR repeats	pfam13414 TPR_11 pfam00515 TPR_1				1	N
	2265137532	or0120	Leucine-rich repeat (LRR) protein	COG4886 Leucine-rich repeat (LRR) protein	pfam12799 LRR_4 pfam00963 Cohesin pfam13860 FlgD_ig	TIGR04183 Por secretion system C-terminal sorting domain			0	N
	2265137554	or0142	FOG: WD40 repeat	COG2319 FOG: WD40 repeat	pfam00400 WD40				0	N
	2265137572	or1182	Tetratricopeptide repeat.	COG5010 Flp pilus assembly protein TadD, contains TPR repeats	pfam13414 TPR_11 pfam00515 TPR_1				0	N
	2265137594	or1205	WD-40 repeat-containing protein	COG2319 FOG: WD40 repeat	pfam00400 WD40				0	Y
	2265137595	or1206	NHL repeat.	COG3391 Uncharacterized conserved protein	pfam01436 NHL				0	N
	2265137598	or1209	Signal transduction histidine kinase	COG0642 Signal transduction histidine kinase	pfam13414 TPR_11 pfam02518 HATPase_c				0	N
	2265137627	or1238	Tetratricopeptide repeat.	COG4785 Lipoprotein NlpI,	pfam13414				0	N

				contains TPR repeats	TPR_11					
	2265137656	or0168	ASPIC and UnbV./FG-GAP repeat.		pfam13517 VCBS pfam07593 UnbV_ASPIC				0	N
	2265137723	or1338	Nucleosome binding factor SPN, SPT16 subunit	COG5406 Nucleosome binding factor SPN, SPT16 subunit	pfam13414 TPR_11				0	N
	2265137725	or0171	Peroxiredoxin	COG1225 Peroxiredoxin	pfam00578 AhpC-TSA pfam13414 TPR_11				1	N
	2265137748	or1362	Tetratricopeptide repeat.	COG3063 Tfp pilus assembly protein PilF	pfam00515 TPR_1 pfam13414 TPR_11				1	N
	2265137767	or1377	Bacterial Ig-like domain (group 1)./Leucine Rich Repeat.	COG4886 Leucine-rich repeat (LRR) protein	pfam12799 LRR_4	TIGR04183 Por secretion system C-terminal sorting domain			0	N
	2265137841	or1453	Leucine Rich Repeat.	COG4886 Leucine-rich repeat (LRR) protein	pfam12799 LRR_4				1	Y
	2265137858	or1472	Leucine-rich repeat (LRR) protein	COG4886 Leucine-rich repeat (LRR) protein	pfam12799 LRR_4				1	N
	2265137882	or1496	FOG: WD40 repeat	COG2319 FOG:	pfam00400				0	N

				WD40 repeat	WD40					
	2265137883	or1497	FOG: WD40 repeat	COG2319 FOG: WD40 repeat	pfam00400 WD40				0	Y
	2265137886	or1500	FOG: WD40 repeat	COG2319 FOG: WD40 repeat	pfam00400 WD40				0	Y
	2265137887	or1501	FOG: WD40 repeat	COG2319 FOG: WD40 repeat	pfam00400 WD40				0	N
	2265137888	or1502	FOG: WD40 repeat	COG2319 FOG: WD40 repeat	pfam00400 WD40				0	Y
	2265137890	or1503	FOG: WD40 repeat	COG2319 FOG: WD40 repeat	pfam00400 WD40				0	Y
	2265137891	or1504	FOG: WD40 repeat	COG2319 FOG: WD40 repeat	pfam00400 WD40				0	Y
	2265137892	or1505	Divergent AAA domain./Tetratricopeptide repeat.		pfam04326 AAA_4 pfam13414 TPR_11				0	N
	2265137899	or1513	Uncharacterized protein conserved in bacteria	COG1729 Uncharacterized protein conserved in bacteria	pfam13174 TPR_6 pfam13414 TPR_11 pfam13181 TPR_8				1	N
	2265137901	or1516	RNA polymerase sigma factor, sigma-70 family	COG1595 DNA-directed RNA polymerase specialized sigma subunit, sigma24 homolog	pfam04542 Sigma70_r2 pfam08281 Sigma70_r4_2 pfam13855 LRR_8	TIGR02937 RNA polymerase sigma factor, sigma-70 family			0	N
	2265137912	or0194	ASPIC and UnbV.	COG0457 FOG:	pfam07593 UnbV_ASPIC				0	N

				TPR repeat	pfam13517 VCBS pfam13414 TPR_11					
	2265137924	or0206	FOG: TPR repeat	COG0457 FOG: TPR repeat	pfam13435 Cytochrome_C554 pfam13414 TPR_11				5	N
	2265137991	or1576	FOG: WD40 repeat	COG2319 FOG: WD40 repeat	pfam00400 WD40				0	Y
	2265137996	or1582	FOG: WD40 repeat	COG2319 FOG: WD40 repeat	pfam00400 WD40				0	Y
	2265137997	or1583	FOG: Ankyrin repeat	COG0666 FOG: Ankyrin repeat	pfam12796 Ank_2				0	N
	2265138040	or0225	Glucose dehydrogenase	COG4993 Glucose dehydrogenase	pfam13360 PQQ_2		EC:1.1.5.2 Quinoprotein glucose dehydrogenase.	KO:K00117 gcd quinoprotein glucose dehydrogenase [EC:1.1.5.2]	1	N
	2265138063	or1646	FOG: Ankyrin repeat	COG0666 FOG: Ankyrin repeat	pfam12796 Ank_2 pfam01757 Acyl_transf_3				11	N
	2265138090	or1678	ASPIC and UnbV.		pfam13517 VCBS pfam07593 UnbV_ASPIC				0	N
	2265138119	or1707	Trypsin-like serine proteases, typically periplasmic, contain C-terminal PDZ domain	COG0265 Trypsin-like serine proteases, typically periplasmic, contain C-	pfam13365 Trypsin_2 pfam13414 TPR_11 pfam00515 TPR_1				1	Y

				terminal PDZ domain							
	2265138121	or1709	hypothetical protein		pfam13754 Big_3_4	TIGR04183 Por secretion system C-terminal sorting domain				1	N
	2265138174	or1760	Tetratricopeptide repeat.	COG2956 Predicted N-acetylglucosaminyl transferase	pfam00515 TPR_1 pfam13414 TPR_11 pfam13174 TPR_6					1	N
	2265138305	or1903	Uncharacterized conserved protein	COG2912 Uncharacterized conserved protein	pfam13371 TPR_9 pfam13369 Transglut_core2					0	N
	2265138320	or1920	Tetratricopeptide repeat.	COG4783 Putative Zn-dependent protease, contains TPR repeats	pfam13414 TPR_11					0	Y
	2265138332	or1934	Tetratricopeptide repeat.	COG3063 Tfp pilus assembly protein PilF	pfam13414 TPR_11					0	N
	2265138417	or2002	Leucine-rich repeat (LRR) protein	COG4886 Leucine-rich repeat (LRR) protein	pfam12799 LRR_4					0	N
	2265138446	or2038	Tetratricopeptide repeat./FRG domain.	COG3063 Tfp pilus assembly protein PilF	pfam13374 TPR_10 pfam13414 TPR_11 pfam08867					0	N

					FRG						
	2265138513	or2088	PQQ enzyme repeat.	COG1520 FOG: WD40-like repeat	pfam13360 PQQ_2					1	N
	2265138552	or2135	PQQ enzyme repeat.	COG1520 FOG: WD40-like repeat	pfam13360 PQQ_2					1	Y
	2265138560	or2143	FOG: WD40-like repeat	COG1520 FOG: WD40-like repeat	pfam13360 PQQ_2					1	N
	2265138568	or2151	FOG: WD40-like repeat	COG1520 FOG: WD40-like repeat	pfam13360 PQQ_2					0	N
	2265138585	or2172	FOG: WD40 repeat	COG2319 FOG: WD40 repeat	pfam13860 FlgD_ig pfam00400 WD40	TIGR04183 Por secretion system C-terminal sorting domain				0	Y
	2265138615	or2206	hypothetical protein		pfam13570 PQQ_3					1	N
	2265138618	or2209	FOG: WD40-like repeat	COG1520 FOG: WD40-like repeat	pfam13360 PQQ_2 pfam13570 PQQ_3					1	N
	2265138634	or0296	ASPIC and UnbV./Tetratricopeptide repeat.	COG0457 FOG: TPR repeat	pfam13414 TPR_11 pfam07593 UnbV_ASPIC pfam00515 TPR_1 pfam13517 VCBS					1	N

	2265138641	or2238	FOG: WD40 repeat	COG2319 FOG: WD40 repeat	pfam00400 WD40				0	Y
	2265138642	or2239	FOG: WD40 repeat	COG2319 FOG: WD40 repeat	pfam00400 WD40				0	N
	2265138664	or2265	ASPIC and UnbV.		pfam13517 VCBS pfam07593 UnbV_ASPIC				0	N
	2265138703	or2301	FOG: WD40 repeat	COG2319 FOG: WD40 repeat	pfam07635 PSCyt1 pfam00403 HMA pfam00400 WD40				0	N
	2265138740	or2343	Predicted N-acetylglucosaminyl transferase	COG2956 Predicted N-acetylglucosaminyl transferase	pfam13414 TPR_11 pfam13174 TPR_6				0	N
	2265138794	or0322	Tetratricopeptide repeat.	COG3063 Tfp pilus assembly protein PilF	pfam13414 TPR_11				0	N
	2265138819	or0346	Transglutaminase-like superfamily./Tetratricopeptide repeat.	COG5010 Flp pilus assembly protein TadD, contains TPR repeats	pfam00515 TPR_1 pfam01841 Transglut_core pfam12969 DUF3857 pfam13432 TPR_16 pfam07719 TPR_2				0	N
	2265138839	or2398	Tetratricopeptide repeat.	COG3063 Tfp pilus assembly protein PilF	pfam13414 TPR_11				0	N

	2265138869	or0374	FOG: WD40 repeat	COG2319 FOG: WD40 repeat	pfam00400 WD40				0	Y
	2265138908	or2447	Tetratricopeptide repeat.	COG3063 Tfp pilus assembly protein PilF	pfam13414 TPR_11 pfam12950 TaqI_C				0	N
	2265138970	or2481	Sel1 repeat.	COG0790 FOG: TPR repeat, SEL1 subfamily	pfam08238 Sel1				0	N
	2265139044	or0430	Leucine-rich repeat (LRR) protein	COG4886 Leucine-rich repeat (LRR) protein	pfam00963 Cohesin pfam13860 FlgD_ig pfam12799 LRR_4	TIGR04183 Por secretion system C-terminal sorting domain			0	N
	2265139061	or2569	FOG: WD40 repeat	COG2319 FOG: WD40 repeat	pfam00400 WD40 pfam13860 FlgD_ig	TIGR04183 Por secretion system C-terminal sorting domain			0	N
	2265139079	or2592	WD40-like Beta Propeller Repeat.	COG1520 FOG: WD40-like repeat	pfam13360 PQQ_2				0	N
	2265139111	or2622	Tetratricopeptide repeat.	COG3063 Tfp pilus assembly protein PilF	pfam13414 TPR_11 pfam00515 TPR_1				0	N
	2265139137	or2653	Tetratricopeptide repeat.	COG5010 Flp pilus assembly protein TadD, contains TPR repeats	pfam00515 TPR_1 pfam13414 TPR_11				0	N
	2265139162	or2677	FOG: TPR repeat, SEL1 subfamily	COG0790 FOG: TPR repeat,	pfam08238 Sel1			KO:K07126	0	N

				SEL1 subfamily				K07126		
	2265139174	or2692	Cytochrome c biogenesis factor	COG4235 Cytochrome c biogenesis factor	pfam13414 TPR_11				0	N
	2265139204	or2727	N-acetylmuramoyl-L-alanine amidase	COG0860 N-acetylmuramoyl -L-alanine amidase	pfam01520 Amidase_3 pfam13432 TPR_16		EC:3.5.1.28 N-acetylmuramoyl -L-alanine amidase.	KO:K01448 E3.5.1.28B, amiA, amiB, amiC N-acetylmuramoyl -L-alanine amidase [EC:3.5.1.28]	0	Y
	2265139257	or2756	Cohesin domain.	COG4886 Leucine-rich repeat (LRR) protein	pfam12799 LRR_4 pfam00963 Cohesin				0	N
	2265139279	or2769	FOG: TPR repeat, SEL1 subfamily	COG0790 FOG: TPR repeat, SEL1 subfamily	pfam08238 Sel1				0	N
	2265139314	or2809	Cohesin domain./Leucine Rich Repeat.	COG4886 Leucine-rich repeat (LRR) protein	pfam00963 Cohesin pfam12799 LRR_4				0	N
	2265139323	or2823	FOG: WD40 repeat	COG2319 FOG: WD40 repeat	pfam00400 WD40				0	N
	2265139367	or2855	Cohesin domain./Dockerin type I repeat.	COG4886 Leucine-rich repeat (LRR) protein	pfam00404 Dockerin_1 pfam00963 Cohesin pfam12799 LRR_4	TIGR04183 Por secretion system C-terminal sorting domain			0	N
	2265139466	or2936	FOG: WD40 repeat	COG2319 FOG:	pfam00400				0	N

				WD40 repeat	WD40					
	2265139500	or2976	hypothetical protein		pfam13414 TPR_11 pfam07719 TPR_2				0	Y
	2265139535	or0592	FOG: WD40-like repeat	COG1520 FOG: WD40-like repeat	pfam13360 PQQ_2				0	N
	2265139607	or3084	FOG: WD40 repeat	COG2319 FOG: WD40 repeat	pfam00400 WD40				0	Y
	2265139610	or3089	Cohesin domain.	COG4886 Leucine-rich repeat (LRR) protein	pfam00963 Cohesin pfam12799 LRR_4				0	N
	2265139614	or3098	Uncharacterized protein conserved in bacteria	COG1729 Uncharacterized protein conserved in bacteria	pfam00515 TPR_1 pfam13174 TPR_6 pfam13414 TPR_11 pfam13424 TPR_12				0	N
	2265139617	or3102	Bacterial Ig-like domain (group 2).		pfam02368 Big_2				0	N
	2265139627	or0610	Uncharacterized protein conserved in bacteria	COG1729 Uncharacterized protein conserved in bacteria	pfam13414 TPR_11 pfam08239 SH3_3				3	N
	2265139629	or0612	Tetratricopeptide repeat.	COG5271 AAA ATPase containing von Willebrand factor type A	pfam13414 TPR_11 pfam00515 TPR_1				1	N

				(vWA) domain						
	2265139639	or3125	Bacterial Ig-like domain (group 2).		pfam02368 Big_2				0	N
	2265139650	or3136	Bacterial Ig-like domain (group 2).		pfam02368 Big_2				0	N
	2265139672	or3161	Bacterial Ig-like domain (group 2).		pfam02368 Big_2				0	N
	2265139697	or3193	FOG: Ankyrin repeat	COG0666 FOG: Ankyrin repeat	pfam12796 Ank_2				0	N
	2265139730	or3227	Leucine-rich repeat (LRR) protein	COG4886 Leucine-rich repeat (LRR) protein	pfam00963 Cohesin pfam12799 LRR_4				0	N
	2265139862	or3344	DnaJ-class molecular chaperone with C-terminal Zn finger domain	COG0484 DnaJ-class molecular chaperone with C-terminal Zn finger domain	pfam00226 DnaJ pfam13181 TPR_8				0	N
	2265139865	or3348	Cohesin domain./Planctomycete cytochrome C.	COG4886 Leucine-rich repeat (LRR) protein	pfam13860 FlgD_ig pfam07635 PSCyt1 pfam12799 LRR_4 pfam00963 Cohesin	TIGR04183 Por secretion system C-terminal sorting domain			0	N
	2265139929	or3410	Tetratricopeptide repeat.	COG4783 Putative Zn-dependent protease, contains TPR repeats	pfam00515 TPR_1 pfam13414 TPR_11				0	N

	2265139935	or3416	Predicted RNA-binding protein homologous to eukaryotic snRNP	COG1293 Predicted RNA-binding protein homologous to eukaryotic snRNP	pfam05670 DUF814 pfam05833 FbpA					0	N
	2265140001	or3488	Tetratricopeptide repeat.	COG3063 Tfp pilus assembly protein PilF	pfam13414 TPR_11					1	N
	2265140005	or3492	hypothetical protein	COG1520 FOG: WD40-like repeat	pfam13360 PQQ_2					1	N
	2265140009	or3496	NHL repeat.	COG3391 Uncharacterized conserved protein	pfam01436 NHL					0	N
	2265140033	or3517	Leucine-rich repeat (LRR) protein	COG4886 Leucine-rich repeat (LRR) protein	pfam00963 Cohesin pfam12799 LRR_4					0	N
	2265140126	or3610	Spermidine synthase	COG0421 Spermidine synthase	pfam01564 Spermine_synth pfam13414 TPR_11					9	N
	2265140136	or3621	FOG: WD40 repeat	COG2319 FOG: WD40 repeat	pfam00400 WD40	TIGR04183 Por secretion system C-terminal sorting domain				1	N
	2265140153	or0666	hypothetical protein		pfam12245 Big_3_2 pfam13753 SWM_repeat					1	N

	2265140161	or3632	Leucine-rich repeat (LRR) protein	COG4886 Leucine-rich repeat (LRR) protein	pfam00963 Cohesin pfam13860 FlgD_ig pfam12799 LRR_4	TIGR04183 Por secretion system C-terminal sorting domain			0	N
	2265140251	or3723	Serine/threonine protein kinase	COG0515 Serine/threonine protein kinase	pfam00069 Pkinase				0	N
	2265140264	or3737	FOG: Ankyrin repeat	COG0666 FOG: Ankyrin repeat	pfam12796 Ank_2				0	N
	2265140269	or3742	FOG: WD40 repeat	COG2319 FOG: WD40 repeat	pfam13860 FlgD_ig pfam00400 WD40	TIGR04183 Por secretion system C-terminal sorting domain			0	Y
	2265140277	or3751	Cadherin domain./Bacterial Ig-like domain (group 2).		pfam00028 Cadherin pfam02368 Big_2				0	N
	2265140286	or3766	Leucine-rich repeat (LRR) protein	COG4886 Leucine-rich repeat (LRR) protein	pfam00963 Cohesin pfam00404 Dockerin_1 pfam07635 PSCyt1 pfam12799 LRR_4 pfam13860 FlgD_ig	TIGR04183 Por secretion system C-terminal sorting domain			0	Y
	2265140288	or3768	Leucine Rich Repeat.	COG4886 Leucine-rich repeat (LRR) protein	pfam12799 LRR_4	TIGR04183 Por secretion system C-terminal sorting domain			1	N

	2265140295	or3775	Flp pilus assembly protein TadD, contains TPR repeats	COG5010 Flp pilus assembly protein TadD, contains TPR repeats	pfam04480 DUF559 pfam00515 TPR_1 pfam13414 TPR_11				0	N
	2265140302	or3782	Leucine-rich repeat (LRR) protein	COG4886 Leucine-rich repeat (LRR) protein	pfam12799 LRR_4				0	N
	2265140399	or0739	Low-density lipoprotein receptor repeat class B./SMP-30/Gluconolaconase/LRE-like region.	COG3391 Uncharacterized conserved protein	pfam08450 SGL pfam00058 Ldl_recept_b				1	N
	2265140425	or0073	FOG: WD40-like repeat	COG1520 FOG: WD40-like repeat	pfam13360 PQQ_2				0	N
	2265140429	or0077	Large extracellular alpha-helical protein	COG2373 Large extracellular alpha-helical protein	pfam00515 TPR_1 pfam13432 TPR_16 pfam13174 TPR_6 pfam07703 A2M_N_2 pfam13181 TPR_8 pfam00207 A2M pfam01835 A2M_N				0	Y
	2265140447	or0758	Leucine Rich Repeat.	COG4886 Leucine-rich repeat (LRR) protein	pfam12799 LRR_4				0	N

	2265140454	or0765	Uncharacterized protein conserved in bacteria (DUF2133).	COG2976 Uncharacterized protein conserved in bacteria	pfam13181 TPR_8 pfam09976 TPR_21				1	N
	2265140542	or0098	Periplasmic component of the Tol biopolymer transport system	COG0823 Periplasmic component of the Tol biopolymer transport system	pfam13414 TPR_11 pfam07676 PD40				0	N
	2265140551	or0866	NHL repeat.	COG3391 Uncharacterized conserved protein	pfam01436 NHL				0	N
	2265140560	or0876	FOG: WD40 repeat	COG2319 FOG: WD40 repeat	pfam00400 WD40 pfam13860 FlgD_ig	TIGR04183 Por secretion system C-terminal sorting domain			0	Y
	2265140573	or0889	Tetratricopeptide repeat.	COG5010 Flp pilus assembly protein TadD, contains TPR repeats	pfam13414 TPR_11				1	N
	2265140587	or0906	Two component regulator propeller.	COG0457 FOG: TPR repeat	pfam07494 Reg_prop pfam13414 TPR_11				1	N
	2265140591	or0912	Tetratricopeptide repeat./FRG domain.	COG3063 Tfp pilus assembly protein PilF	pfam08867 FRG pfam13414 TPR_11				0	N

	2265140593	or0914	FOG: TPR repeat	COG0457 FOG: TPR repeat	pfam07719 TPR_2 pfam12950 TaqI_C pfam13414 TPR_11				0	N
	2265140594	or0916	Tetratricopeptide repeat./TIR domain.	COG5010 Flp pilus assembly protein TadD, contains TPR repeats	pfam13676 TIR_2 pfam13414 TPR_11				0	N
	2265140595	or0917	Tetratricopeptide repeat.	COG5010 Flp pilus assembly protein TadD, contains TPR repeats	pfam13414 TPR_11 pfam07719 TPR_2 pfam13676 TIR_2				0	N
	2265140596	or0918	Tetratricopeptide repeat.	COG4916 Uncharacterized protein containing a TIR (Toll-Interleukin 1-resistance) domain	pfam13676 TIR_2 pfam13414 TPR_11				0	N
	2265140624	or0948	ASPIC and UnbV.		pfam13517 VCBS pfam07593 UnbV_ASPIC				1	Y
	2265140694	or1024	Tfp pilus assembly protein PilF	COG3063 Tfp pilus assembly protein PilF	pfam00515 TPR_1 pfam13414 TPR_11 pfam13174 TPR_6				0	N
	2265140705	or1035	Sel1 repeat.	COG0790 FOG: TPR repeat,	pfam08238 Sel1				0	N

				SEL1 subfamily						
	2265140709	or1040	Sel1 repeat./HNH endonuclease.	COG0790 FOG: TPR repeat, SEL1 subfamily	pfam08238 Sel1 pfam01844 HNH				0	N
	2265140710	or1042	Leucine Rich Repeat.	COG4886 Leucine-rich repeat (LRR) protein	pfam12799 LRR_4 pfam13516 LRR_6				1	Y
	2265140753	or1087	Uncharacterized protein conserved in bacteria	COG2852 Uncharacterized protein conserved in bacteria	pfam13414 TPR_11 pfam00515 TPR_1 pfam04480 DUF559				0	N
	2265140755	or1089	Tetratricopeptide repeat.	COG5010 Flp pilus assembly protein TadD, contains TPR repeats	pfam13414 TPR_11				0	N
	2265140773	or1108	Leucine Rich Repeat./Cadherin domain.	COG4886 Leucine-rich repeat (LRR) protein	pfam00028 Cadherin pfam13855 LRR_8				1	N
	2265140777	or1113	Leucine-rich repeat (LRR) protein	COG4886 Leucine-rich repeat (LRR) protein	pfam12799 LRR_4				0	N
	2265140787	or1124	FOG: WD40 repeat	COG2319 FOG: WD40 repeat	pfam00400 WD40				3	N
4G	2265142434	or0078	FOG: WD40 repeat	COG2319 FOG: WD40 repeat	pfam00400 WD40 pfam08662 eIF2A				0	N

	2265142435	or0079	FOG: WD40 repeat	COG2319 FOG: WD40 repeat	pfam00400 WD40				0	N
	2265142463	or1167	FOG: WD40 repeat	COG2319 FOG: WD40 repeat	pfam00400 WD40				0	N
	2265142489	or1193	FOG: WD40-like repeat	COG1520 FOG: WD40-like repeat	pfam13360 PQQ_2 pfam01011 PQQ				2	N
	2265142494	or1198	hypothetical protein	COG1729 Uncharacterized protein conserved in bacteria	pfam13174 TPR_6				0	N
	2265142496	or1200	Cytochrome c biogenesis factor	COG4235 Cytochrome c biogenesis factor	pfam13517 VCBS pfam13432 TPR_16 pfam07593 UnbV_ASPIC pfam13414 TPR_11				0	N
	2265142498	or1202	Periplasmic component of the Tol biopolymer transport system	COG0823 Periplasmic component of the Tol biopolymer transport system	pfam07676 PD40 pfam13414 TPR_11				1	N
	2265142499	or1203	ASPIC and UnbV.		pfam07593 UnbV_ASPIC pfam13517 VCBS				0	Y
	2265142512	or1310	hypothetical protein		pfam07719 TPR_2				0	N

	2265142519	or1318	Zn-dependent hydrolases, including glyoxylases	COG0491 Zn-dependent hydrolases, including glyoxylases	pfam07719 TPR_2 pfam00753 Lactamase_B				0	N
	2265142549	or1591	hypothetical protein		pfam13414 TPR_11				1	N
	2265142582	or1743	ABC-type Fe3+-hydroxamate transport system, periplasmic component	COG0614 ABC-type Fe3+-hydroxamate transport system, periplasmic component	pfam13174 TPR_6				1	N
