# Supplementary material for: The Candidate Phylum Poribacteria by Single-Cell Genomics: New Insights into Phylogeny, Cell-Compartmentation, Eukaryote-Like Repeat Proteins, and Other Genomic Features
Source: PLoS One. 2014 Jan 31;9(1):e87353. doi: 10.1371/journal.pone.0087353 (PMC3909097; doi:10.1371/journal.pone.0087353)
Supplement: Table S9 — List of total repeat and eukaryote like protein domain encoding genes of poribacterial SAGs showing the number of genes (# genes), the number of genes with transmemebrane helicies (# TMH), percentage of genes with transmembrane helicies (% TMH), number of genes with signal peptide (# SP), and percentage of genes with signal peptide (% SP) for each domain. (PDF) [file pone.0087353.s009.pdf]

Table S9: List of total repeat and eukaryote like protein domain encoding genes of poribacterial SAGs showing the number of genes (# genes), the number of genes with transmemebrane helicies (# TMH), percentage of genes with transmembrane helicies (% TMH), number of genes with signal peptide (# SP), and percentage of genes with signal peptide (% SP) for each domain.

		# Genes	# TMH	% TMH	# SP	% SP
pfam00023	Ankyrin repeat	1	0	0.00	0	0.00
pfam00028	Cadherin domain	8	5	62.50	2	25.00
pfam00041	Fibronectin type III domain	3	2	66.67	1	33.33
pfam00058	Low-density lipoprotein receptor repeat class B	7	6	85.71	1	14.29
pfam00069	Protein kinase domain	4	1	25.00	0	0.00
pfam00400	WD domain, G-beta repeat	133	32	24.06	41	30.83
pfam00404	Dockerin type I repeat	13	3	23.08	4	30.77
pfam00515	Tetratricopeptide repeat	47	12	25.53	5	10.64
pfam01011	PQQ enzyme repeat	2	2	100.00	0	0.00
pfam01436	NHL repeat	10	2	20.00	0	0.00
pfam02368	Bacterial Ig-like domain (group 2)	5	0	0.00	0	0.00
pfam02369	Bacterial Ig-like domain (group 1)	2	0	0.00	0	0.00
pfam05833	Fibronectin-binding protein A N-terminus (FbpA)	3	0	0.00	0	0.00
pfam07593	ASPIC and UnbV	23	9	39.13	8	34.78
pfam07719	Tetratricopeptide repeat	29	10	34.48	2	6.90
pfam08238	Sel1 repeat	6	0	0.00	0	0.00
pfam09976	Tetratricopeptide repeat	2	2	100.00	0	0.00
pfam12245	Bacterial Ig-like domain (group 3)	1	1	100.00	0	0.00
pfam12796	Ankyrin repeats (3 copies)	11	2	18.18	1	9.09
pfam12799	Leucine Rich repeats (2 copies)	56	20	35.71	15	26.79
pfam13174	Tetratricopeptide repeat	21	8	38.10	3	14.29
pfam13176	Tetratricopeptide repeat	1	0	0.00	0	0.00
pfam13181	Tetratricopeptide repeat	15	6	40.00	6	40.00
pfam13205	Bacterial Ig-like domain	6	3	50.00	3	50.00
pfam13360	PQQ-like domain	24	16	66.67	7	29.17
pfam13371	Tetratricopeptide repeat	4	2	50.00	0	0.00
pfam13374	Tetratricopeptide repeat	1	0	0.00	0	0.00

pfam13414	Tetratricopeptide repeat	158	51	32.28	15	9.49
pfam13424	Tetratricopeptide repeat	8	4	50.00	0	0.00
pfam13428	Tetratricopeptide repeat	2	0	0.00	1	50.00
pfam13429	Tetratricopeptide repeat	1	1	100.00	0	0.00
pfam13431	Tetratricopeptide repeat	1	1	100.00	0	0.00
pfam13432	Tetratricopeptide repeat	16	8	50.00	3	18.75
pfam13516	Leucine Rich repeat	3	2	66.67	2	66.67
pfam13517	Repeat domain in Vibrio. Colwellia, Bradyrhizobium and Shewanella	30	10	33.33	9	30.00
pfam13570	PQQ-like domain	4	4	100.00	1	25.00
pfam13637	Ankyrin repeats (many copies)	1	0	0.00	0	0.00
pfam13750	Bacterial Ig-like domain (group 3)	1	1	100.00	1	100.00
pfam13754	Bacterial Ig-like domain (group 3)	1	1	100.00	0	0.00
pfam13855	Leucine rich repeat	2	1	50.00	0	0.00
pfam13857	Ankyrin repeats (many copies)	1	0	0.00	0	0.00
