# Supplementary material for: The Candidate Phylum Poribacteria by Single-Cell Genomics: New Insights into Phylogeny, Cell-Compartmentation, Eukaryote-Like Repeat Proteins, and Other Genomic Features
Source: PLoS One. 2014 Jan 31;9(1):e87353. doi: 10.1371/journal.pone.0087353 (PMC3909097; doi:10.1371/journal.pone.0087353)
Supplement: Table S10 — phyH domain distribution on publically available genomes. (PDF) [file pone.0087353.s010.pdf]

Table S10: phyH domain distribution on publically available genomes.

Domain	Genome	Total # genes on genome	# phyH genes	phyH genes of total genes (%)	average phyH genesof total genes (%)	maximum % of phyH genes of total genes
Archaea	Halonotius sp. J07HN6 (version 2)	2965	2	0,067	0,038	0,067
	Nitrosopumilus maritimus SCM1	1842	1	0,054		
	Candidatus Nitrosopumilus koreensis AR1	1931	1	0,052		
	Candidatus Nitrosopumilus sp. AR2	2014	1	0,050		
	Halomicrobium mukohataei arg-2, DSM 12286	3475	1	0,029		
	halophilic archaeon True-ADL	3520	1	0,028		
	Halomicrobium katesii DSM 19301	3876	1	0,026		
	Halalkalicoccus jeotgali B3, DSM 18796	3915	1	0,026		
	Halalkalicoccus jeotgali B3, DSM 18796	3925	1	0,025		
	Natronorubrum tibetense DSM 13204	4867	1	0,021		
Bacteria / Poribacteria	Candidatus Poribacteria sp. WGA-4E	3319	123	3,706	2,705	3,706
	Candidatus Poribacteria sp. WGA-4G	178	6	3,371		
	Candidatus Poribacteria sp. WGA-4C	1634	41	2,509		
	Candidatus Poribacteria sp. WGA-3G	4820	114	2,365		
	Candidatus Poribacteria sp. WGA-4CII	478	11	2,301		

	Candidatus Poribacteria sp. WGA-3A	505	10	1,980		
Bacteria	gamma proteobacterium SCGC AAA076-D02	1830	9	0,492		0,492
	alpha proteobacterium SCGC AAA160-J14	817	4	0,490		
	gamma proteobacterium SCGC AAA076-E13	1067	5	0,469		
	Candidatus Pelagibacter-like (SAR11) HIMB140	1535	7	0,456		
	gamma proteobacterium SCGC AAA076-F14	1828	8	0,438		
	candidate division EM 19 bacterium SCGC AAA471-D06 (GBS-N_001_7)	232	1	0,431		
	alpha proteobacterium SCGC AAA300-B11	239	1	0,418		
	Candidatus Pelagibacter ubique SAR11 HTCC8051	1498	6	0,401		
	alpha proteobacterium SCGC AAA536-G10	2337	8	0,342		
	gamma proteobacterium SCGC AAA076-D13	1772	6	0,339		
	Firmicutes bacterium JGI 0000119-C08	314	1	0,318		
	Actinopolymorpha alba DSM 45243	7545	24	0,318		
	Bradyrhizobium sp. JGI 001005-E20	344	1	0,291		
	Marine gamma proteobacterium sp. HTCC2080	3229	9	0,279		
	Candidatus Pelagibacter ubique SAR11 HTCC9022	1464	4	0,273		

	alpha proteobacterium sp. HIMB5	1466	4	0,273		
	Alphaproteobacteria sp. SAR11 HIMB5	1467	4	0,273		
	Thalassobaculum salexigens DSM 19539	4776	13	0,272		
	Candidatus Pelagibacter-like (SAR11) HIMB083	1505	4	0,266		
	alpha proteobacterium SCGC AAA024-N17	397	1	0,252		
	alpha proteobacterium SCGC AAA300-J16	1201	3	0,250		
	Marine gamma proteobacterium sp. HTCC2143	3709	9	0,243		
	Gammaproteobacteria sp. OM60 HIMB55	2511	6	0,239		
	Candidatus Puniceispirillum marinum IMCC1322	2582	6	0,232		
	alpha proteobacterium SCGC AAA536-K22	2189	5	0,228		
	SAR116 cluster alpha proteobacterium sp. HIMB100	2376	5	0,210		
	Nisaea sp BAL199	6182	13	0,210		
	Candidatus Pelagibacter sp. HTCC7211	1481	3	0,203		
	gamma proteobacterium SCGC AAA300-D14	1484	3	0,202		
	Flectobacillus major VKMB-859, DSM 103	5011	10	0,200		
	Paenibacillus harenae DSM 16969	6376	12	0,188		
	Kordiimonas gwangyangensis DSM	3778	7	0,185		

	19435					
	Sphingopyxis alaskensis RB2256	3265	6	0,184		
	Paenibacillus alginolyticus DSM 5050	8231	15	0,182		
	Inquilinus limosus DSM 16000	7081	12	0,169		
	Paenibacillus terrigena DSM 21567	5932	10	0,169		
	Wolbachia sp. wRi	1187	2	0,168		
	Planctomyces maris DSM 8797	6542	11	0,168		
	Legionella shakespearei DSM 23087	3035	5	0,165		
	Planctomyces brasiliensis IFAM 1448, DSM 5305	4865	8	0,164		
	Pedobacter heparinus HIM 762-3, DSM 2366	4339	7	0,161		
	alpha proteobacterium SCGC AAA015-O19	1891	3	0,159		
	Verrucomicrobia bacterium SCGC AAA164-E04	3814	6	0,157		
	Paenibacillus sp. JDR-2	6410	10	0,156		
	Marine gamma proteobacterium sp. HTCC2148	3871	6	0,155		
	Dyadobacter fermentans NS114, DSM 18053	5854	9	0,154		
	Acidobacteriaceae sp. DSM 24297	5863	9	0,154		
	Paenibacillus sp. HGF5	6579	10	0,152		
	Haliea salexigens DSM 19537	4016	6	0,149		

	alpha proteobacterium SCGC AAA076-C03	2028	3	0,148		
	Verrucomicrobia bacterium SCGC AAA164-I21	1355	2	0,148		
	Sphingobium sp. SYK-6	4121	6	0,146		
	Candidatus Pelagibacter ubique SAR11 HTCC9565	1386	2	0,144		
	alpha proteobacterium SCGC AAA300-J04	697	1	0,143		
	Candidatus Pelagibacter ubique SAR11 HTCC1013	1411	2	0,142		
	Flavobacteria bacterium MS220-5C	707	1	0,141		
	Micromonospora carbonacea var. africana. ATCC 39149	5692	8	0,141		
	Kribbella flavida IFO 14399, DSM 17836	7149	10	0,140		
	Paenibacillus lautus Y412MC10	6444	9	0,140		
	SAR86 cluster bacterium SAR86E	1441	2	0,139		
	Actinospica robiniae DSM 44927	8726	12	0,138		
	Rhodobacterales sp. HTCC2255 (original sequence, contaminants removed)	2209	3	0,136		
	Rhodobacterales sp. HTCC2150	3713	5	0,135		
	Parvibaculum lavamentivorans DS-1	3714	5	0,135		
	Sphingobium japonicum UT26S	4460	6	0,135		

	Proteobacteria bacterium JGI 0000113-E04	5242	7	0,134		
	Kribbella catacumbae DSM 19601	9069	12	0,132		
	Paenibacillus sp. HGF7	6074	8	0,132		
	Pseudovibrio sp. JE062	5323	7	0,132		
	candidate division EM 19 bacterium SCGC AAA471-D06 (Combined_Assembly_EM19_2__Oct-Spa1-106)	1536	2	0,130		
	Haliangium ochraceum SMP-2, DSM 14365	6952	9	0,129		
	Wolbachia endosymbiont of Drosophila simulans	781	1	0,128		
	Pseudovibrio sp. FO-BEG1	5560	7	0,126		
	Brachybacterium faecium 6-10, DSM 4810	3189	4	0,125		
	Verrucomicrobia bacterium SCGC AAA164-A21 (genbank_version)	1613	2	0,124		
	Paenibacillus daejeonensis DSM 15491	6575	8	0,122		
	Roseobacter denitrificans OCh 114	4201	5	0,119		
	Sphingobium chlorophenolicum L-1	4224	5	0,118		
	Rhodobacterales sp. HTCC2083	4226	5	0,118		
	Arenibacter certesii DSM 19833	4235	5	0,118		
	Beutenbergia cavernae HKI 0122, DSM 12333	4278	5	0,117		
	Cyclobacterium marinum	5151	6	0,116		

	Raj, DSM 745					
	candidate division EM 19 bacterium JGI 0000001-B9 (GBS-A_001_114)	860	1	0,116		
	Maribacter sp. HTCC2170	3455	4	0,116		
	Marinomonas mediterranea MMB-1, ATCC 700492	4326	5	0,116		
	Nisaea denitrificans DSM 18348	4346	5	0,115		
	Thalassospira lucentensis DSM 14000	4437	5	0,113		
	alpha proteobacterium SCGC AAA536-B06	1789	2	0,112		
	Flavobacteria bacterium MS024-2A	1811	2	0,110		
	Nocardia sp. BMG111209	8197	9	0,110		
	Ahrensia sp. R2A130	3673	4	0,109		
	Loktanella sp. CCS2	3703	4	0,108		
	Roseobacter litoralis Och 149	4668	5	0,107		
	Thalassiobium sp. R2A62	3744	4	0,107		
	Glaciecola agarilytica 4H-3-7+YE-5	4688	5	0,107		
	Synechococcus sp. RS9917	2822	3	0,106		
	Streptomyces cattleya NRRL 8057	7585	8	0,105		
	Leucothrix mucor DSM 2157	4767	5	0,105		
	Streptomyces cattleya DSM 46488	7650	8	0,105		
	Arcobacter sp. L	2919	3	0,103		
	Bordetella sp. FB-8, DSM	3906	4	0,102		

	24873					
	Sphingomonas sp. URHD0007	2941	3	0,102		
	Hahella chejuensis KCTC 2396	6875	7	0,102		
	Spirosoma panaciterrae DSM 21099	6885	7	0,102		
	Synechococcus sp. CC9616	2958	3	0,101		
	Gamma proteobacterium sp. NOR51-B	2972	3	0,101		
	Azoarcus sp. KH32C	2994	3	0,100		
	Legionella pneumophila pneumonophila 570-CO-H, ATCC 43290	2996	3	0,100		
	Legionella pneumophila pneumophila Philadelphia-1	3003	3	0,100		
	Salinispora pacifica CNY202	5016	5	0,100		
	Synechococcus sp. RS9916	3010	3	0,100		
	Amycolatopsis alba DSM 44262	9036	9	0,100		
	Runella slithyformis LSU4, DSM 19594	6025	6	0,100		
	Nocardia sp. CNY236	5041	5	0,099		
	Burkholderia sp. WSM3556	7123	7	0,098		
	Spirosoma linguale DSM 74	7130	7	0,098		
	Legionella pneumophila Lens	3062	3	0,098		
	Hellea balneolensis DSM 19091	3091	3	0,097		
	Streptomyces sp. CNY243	7235	7	0,097		
	Streptomyces sp. CNQ766	7248	7	0,097		

	Hahella ganghwensis DSM 17046	6233	6	0,096		
	Prochlorococcus marinus MIT 9303	3133	3	0,096		
	Ktedonobacter racemifer SOSP1-21, DSM 44963	11540	11	0,095		
	candidate division EM 19 bacterium JGI 0000001-G10 (GBS-A_001_117)	1050	1	0,095		
	Stigmatella aurantiaca DW4/3-1	8407	8	0,095		
	Streptomyces sp. CNS335	7389	7	0,095		
	Streptomyces sp. CNQ865	7455	7	0,094		
	Plesiocystis pacifica SIR-1	8542	8	0,094		
	Paenibacillus mucilaginosus K02	7476	7	0,094		
	Stigmatella aurantiaca DW4/3-1	8596	8	0,093		
	Runella zeae DSM 19591	6467	6	0,093		
	Legionella pneumophila 2300/99 Alcoy	3242	3	0,093		
	Pseudonocardia spinosispora DSM 44797	8664	8	0,092		
	Xenococcus sp. PCC 7305	5421	5	0,092		
	Ruegeria sp. KLH11	4338	4	0,092		
	Jannaschia sp. CCS1	4339	4	0,092		
	Catelliglobosispora koreensis DSM 44566	7606	7	0,092		
	Legionella pneumophila Corby	3266	3	0,092		
	Ruegeria pomeroyi DSS-3	4355	4	0,092		

	Streptomyces flavidovirens DSM 40150	6544	6	0,092		
	Emticicia oligotrophica GPTSA100-15, DSM 17448	4365	4	0,092		
	Legionella pneumophila Paris	3280	3	0,091		
	Actinomadura cremea rifamycini DSM 43936	7705	7	0,091		
	Pseudoalteromonas atlantica T6c	4405	4	0,091		
	beta proteobacterium SCGC AAA027-K21	2203	2	0,091		
	gamma proteobacterium SCGC AAA076-P09	1109	1	0,090		
	Legionella pneumophila 130b	3333	3	0,090		
	Spirulina major PCC 6313	4460	4	0,090		
	Dyadobacter alkalitolerans DSM 23607	5578	5	0,090		
	Streptomyces hygroscopicus jinggangensis 5008	8935	8	0,090		
	Ruegeria sp. TW15	4470	4	0,089		
	Anabaena circinalis AWQC131C (Submitted file with automatic translation by Kostas)	4475	4	0,089		
	Novosphingobium sp. AP12	5607	5	0,089		
	Burkholderia sp. YI23	7886	7	0,089		
	Wolbachia pipientis wAlbB	1127	1	0,089		
	Ralstonia sp. JGI 0001001-C06	9070	8	0,088		
	beta proteobacterium JGI 0001001-A11	12601	11	0,087		

	Achromobacter xylosoxidans A8	6885	6	0,087		
	Burkholderia sp. Ch1-1	8077	7	0,087		
	Oceanospirillum beijerinckii DSM 7166	4634	4	0,086		
	Pseudomonas azotifigens DSM 17556	4636	4	0,086		
	SAR406 cluster bacterium JGI 0000113-D11	2324	2	0,086		
	Sphingobium sp. AP49	4658	4	0,086		
	Ralstonia sp. JGI 0001001-B07	13993	12	0,086		
	Verrucomicrobia bacterium SCGC AAA164-M04	2340	2	0,085		
	Burkholderia glumae BGR1	5854	5	0,085		
	Burkholderia cenocepacia HI2424	7050	6	0,085		
	Leeia oryzae DSM 17879	3526	3	0,085		
	Bacteroidetes bacterium JGI 0001001-A08	3540	3	0,085		
	Novosphingobium sp. PP1Y	4731	4	0,085		
	Rhizobium sp. JGI 0001002-E20	1187	1	0,084		
	Burkholderia cenocepacia MC0-3	7169	6	0,084		
	Halomonas anticariensis DSM 16096	4807	4	0,083		
	Burkholderia cenocepacia PC184	6024	5	0,083		
	Prochlorococcus sp. CC9902	2410	2	0,083		
	Arcobacter sp. CAB	3617	3	0,083		

	Streptomyces sp. CNB091	7272	6	0,083		
	Burkholderia sp. WSM4176	8497	7	0,082		
	Marine gamma proteobacterium sp. HTCC2207	2429	2	0,082		
	Bradyrhizobium elkanii WSM2783	9734	8	0,082		
	Litoreibacter arenae DSM 19593	3657	3	0,082		
	Dyadobacter beijingensis DSM 21582	6125	5	0,082		
	Silicibacter lacuscaerulensis ITI-1157	3677	3	0,082		
	Bradyrhizobium sp. Ai1a-2	8584	7	0,082		
	Armatimonadetes bacterium JGI 0000077-K19 (TAbiofilm_001_240)	1234	1	0,081		
	Octadecabacter antarcticus 307	4939	4	0,081		
	Actinokineospora enzanensis DSM 44649	7409	6	0,081		
	Pseudomonas chlororaphis chlororaphis GP72 (Draft1)	6176	5	0,081		
	Pseudomonas sp. GM17	6188	5	0,081		
	Niabella aurantiaca DSM 17617	4964	4	0,081		
	Nautella italica R11	3725	3	0,081		
	Gamma proteobacterium sp. NOR5-3	3726	3	0,081		
	Burkholderia thailandensis MSMB43	7481	6	0,080		
	Streptomyces sp. CNQ329	6241	5	0,080		

	Streptomyces sulphureus DSM 40104	6252	5	0,080		
	Cyanobacterium sp. ESFC-1	5006	4	0,080		
	Streptomyces sp. CNT371	7514	6	0,080		
	Paenibacillus mucilaginosus 3016	7528	6	0,080		
	Rudaea cellulosilytica DSM 22992	3775	3	0,079		
	Runella limosa DSM 17973	6298	5	0,079		
	Verrucomicrobia bacterium SCGC AAA300-K03	1262	1	0,079		
	Methylobacterium nodulans ORS 2060	8885	7	0,079		
	Streptomyces scabrisporus DSM 41855	10155	8	0,079		
	Cellvibrio japonicus Ueda107	3811	3	0,079		
	Ralstonia pickettii 12J	5092	4	0,079		
	Pusillimonas sp. T7-7	3826	3	0,078		
	Synechococcus sp. BL107	2555	2	0,078		
	Marinimicrobium agarilyticum DSM 16975	3836	3	0,078		
	Mycobacterium sp. URHD0025	6402	5	0,078		
	Azotobacter vinelandii DJ, ATCC BAA-1303	5133	4	0,078		
	Oceanicola granulosus HTCC2516	3855	3	0,078		
	Bacteroidetes bacterium SCGC AAA027-G08	1294	1	0,077		
	Prochlorococcus sp. WH8102	2588	2	0,077		

	Pseudomonas stutzeri JM3000, DSM 10701	3888	3	0,077		
	Alcaligenes faecalis ANA	6490	5	0,077		
	Phenylobacterium zucineum HLK1	3899	3	0,077		
	Segetibacter koreensis DSM 18137	5211	4	0,077		
	Calothrix desertica PCC 7102	10426	8	0,077		
	Streptomyces viridochromogenes DSM 40736	7831	6	0,077		
	Paenibacillus ginsengihumi DSM 21568	5222	4	0,077		
	Wolbachia endosymbiont wVitB of Nasonia vitripennis	1308	1	0,076		
	Stackebrandtia nassauensis LLR-40K-21, DSM 44728	6541	5	0,076		
	Wolbachia endosymbiont of Drosophila melanogaster	1312	1	0,076		
	Burkholderia glumae LMG 2196	5249	4	0,076		
	Streptomyces sp. SA3_actG	6612	5	0,076		
	Sphingomonas jaspsi DSM 18422	2646	2	0,076		
	Paenibacillus mucilaginosus KNP414	7956	6	0,075		
	Cupriavidus necator JMP134	6631	5	0,075		
	Burkholderia cenocepacia AU 1054	6637	5	0,075		
	Methylotenera sp. 1P/1	2655	2	0,075		
	Methylophilaceae bacterium	2656	2	0,075		

	11					
	Sphingomonas sp. SKA58	3985	3	0,075		
	Salinispora pacifica CNS996	5349	4	0,075		
	Mycobacterium massiliense GO 06	2677	2	0,075		
	Rudanella lutea DSM 19387	5356	4	0,075		
	Pseudomonas sp. URMO17WK12:I7	5363	4	0,075		
	Pseudomonas vranovensis DSM 16006	5363	4	0,075		
	Mesorhizobium loti WSM1293	6711	5	0,075		
	Aestuariimicrobium kwangyangense DSM 21549	2692	2	0,074		
	Novosphingobium aromaticivorans DSM 12444	4038	3	0,074		
	Pleurocapsa sp. PCC 7319	6749	5	0,074		
	Corallococcus coralloides DSM 2259	8101	6	0,074		
	Terrimonas ferruginea DSM 30193	4053	3	0,074		
	Chthoniobacter flavus Ellin428	6778	5	0,074		
	Frankia alni ACN14a	6795	5	0,074		
	Microcoleus sp. PCC 7113	6822	5	0,073		
	Sphingomonas wittichii RW1	5463	4	0,073		
	Spirosoma spitsbergense DSM 19989	6830	5	0,073		
	Saccharophagus degradans 2-40	4114	3	0,073		
	Caulobacter sp. K31	5499	4	0,073		

	Rhodococcus opacus B4	8259	6	0,073		
	Ralstonia pickettii 12D	5518	4	0,072		
	Roseovarius sp. TM1035	4158	3	0,072		
	Rivularia sp. PCC 7116	6946	5	0,072		
	Candidatus Pelagibacter ubique SAR11 HTCC1062	1394	1	0,072		
	Burkholderia ambifaria IOP40-10	6979	5	0,072		
	Salinisphaera shabanensis E1L3A	4196	3	0,071		
	Frankia sp. CN3	8412	6	0,071		
	Kiloniella laminariae DSM 19542	4212	3	0,071		
	Microscilla marina ATCC 23134	8445	6	0,071		
	Labrys methylaminiphilus JLW10	7043	5	0,071		
	gamma proteobacterium SCGC AAA076-P13	1410	1	0,071		
	Flavobacteria MS024-3C	1411	1	0,071		
	Burkholderia sp. WSM2230	5648	4	0,071		
	Rhizobium sp. Pop5	7071	5	0,071		
	Pseudomonas resinovorans DSM 21078	5661	4	0,071		
	Porphyra umbilicalis P.um.1-endophyte10645 (Porphyra_umbilicalis_P.um.1-endophyte10645)	7084	5	0,071		
	Cecembia lonarensis LW9	4259	3	0,070		
	Algoriphagus sp. PR1	4265	3	0,070		
	Burkholderia thailandensis	5696	4	0,070		

	E264, ATCC 700388					
	Duganella violaceinigra DSM 15887	5697	4	0,070		
	Mesorhizobium amorphae CCNWGS0123	7136	5	0,070		
	Cohnella laeviribosi DSM 21336	4284	3	0,070		
	Burkholderia thailandensis E264	5727	4	0,070		
	Reinekea sp. MED297	4301	3	0,070		
	Burkholderia ambifaria MEX-5	7181	5	0,070		
	Stappia stellulata DSM 5886	4314	3	0,070		
	Rhizobium leguminosarum bv. phaseoli 4292	7193	5	0,070		
	Candidatus Nitrospira defluvii	4317	3	0,069		
	gamma proteobacterium IMCC1989	2880	2	0,069		
	Burkholderia pseudomallei S13	5763	4	0,069		
	Terriglobus saanensis SP1PR4, DSM 23119	4333	3	0,069		
	Burkholderia sprentiae WSM5005	7223	5	0,069		
	gamma proteobacterium IMCC3088	2893	2	0,069		
	Spirosoma luteum DSM 19990	5791	4	0,069		
	Achromobacter piechaudii ATCC 43553	5816	4	0,069		
	Porphyra umbilicalis P.um.1-endophyte06694 (Porphyra_umbilicalis_P.um.	5828	4	0,069		

	1-endophyte06694)					
	Rhizobium leguminosarum bv phaseoli FA23	7285	5	0,069		
	Rickettsiella grylli	1457	1	0,069		
	Tsukamurella paurometabola 33, DSM 20162	4391	3	0,068		
	Thermobaculum terrenum YNP1, ATCC BAA-798	2933	2	0,068		
	Pseudomonas syringae CC1458 (CC1458)	5869	4	0,068		
	Flavobacterium sp. CF136	4422	3	0,068		
	Scytonema hofmanni UTEX 2349	7384	5	0,068		
	Saccharospirillum impatiens DSM 12546	4443	3	0,068		
	Candidatus Pelagibacter sp. IMCC9063	1482	1	0,067		
	Deinococcus pimensis KR-235, DSM 21231	4452	3	0,067		
	Polymorphum gilvum SL003B-26A1	4453	3	0,067		
	Burkholderia pseudomallei K96243	5942	4	0,067		
	Flexithrix dorotheae DSM 6795	7446	5	0,067		
	Alcanivorax dieselolei B5	4470	3	0,067		
	Mycobacterium kansasii ATCC 12478	5962	4	0,067		
	Collimonas fungivorans Ter331	4493	3	0,067		
	Burkholderia gladioli BSR3	7493	5	0,067		

	Nocardia farcinica IFM 10152	6011	4	0,067		
	Pseudomonas sp. URIL14HWK12:I12	4517	3	0,066		
	Pseudomonas sp. URIL14HWK12:I10	4522	3	0,066		
	Pseudomonas sp. URIL14HWK12:I11	4524	3	0,066		
	Pseudomonas sp. URIL14HWK12:I9	4527	3	0,066		
	Janthinobacterium lividum PAMC 25724	4546	3	0,066		
	Verrucomicrobia bacterium SCGC AAA164-L15 (genbank_version)	3031	2	0,066		
	Cupriavidus sp. JGI 0001016-D21	1520	1	0,066		
	Blastopirellula marina SH 106T, DSM 3645	6090	4	0,066		
	Caenispirillum salinarum AK4	4574	3	0,066		
	Candidatus Frankia datiscae Dg1	4579	3	0,066		
	alpha proteobacterium sp. HIMB59	1532	1	0,065		
	Flavobacterium sp. F52	4601	3	0,065		
	Erythrobacter litoralis HTCC2594	3068	2	0,065		
	Marinomonas sp. MWYL1	4609	3	0,065		
	Cyanothece sp. PCC 8801	4615	3	0,065		
	Chitinimonas koreensis DSM 17726	4621	3	0,065		
	Paenibacillus pinihumi DSM	6169	4	0,065		

	23905					
	Cupriavidus taiwanensis STM6070	6182	4	0,065		
	Calothrix sp. PCC 7507	6250	4	0,064		
	Burkholderia cepacia 383	7828	5	0,064		
	Cyanothece sp. PCC 8802	4697	3	0,064		
	Endozoicomonas elysicola DSM 22380	4722	3	0,064		
	Micromonospora sp. L5	6326	4	0,063		
	Hydrocarboniphaga effusa AP103	4756	3	0,063		
	Cohnella thermotolerans DSM 17683	4769	3	0,063		
	Rhizobium sp. 2MFCol3.1	6365	4	0,063		
	Pseudoalteromonas piscicida ATCC 15057	4775	3	0,063		
	Kineococcus radiotolerans SRS30216	4785	3	0,063		
	Nitrosococcus oceani C-107, ATCC 19707	3190	2	0,063		
	Streptomyces hygroscopicus ATCC 53653	9574	6	0,063		
	Azospirillum irakense DSM 11586	4801	3	0,062		
	Pirellula staleyi DSM 6068	4825	3	0,062		
	Streptomyces sp. TAA040	4829	3	0,062		
	Erythrobacter sp. NAP1	3226	2	0,062		
	Salinispora pacifica CNT029	4847	3	0,062		
	Marinobacterium rhizophilum DSM 18822	4853	3	0,062		

	Streptomyces canus 299MFChir4.1	9710	6	0,062		
	Nitrosococcus watsoni C-113	3245	2	0,062		
	Robiginitalea biformata HTCC2501	3255	2	0,061		
	Burkholderia mimosarum STM3621	8145	5	0,061		
	Polaromonas sp. CF318	4889	3	0,061		
	Henriciella marina DSM 19595	3260	2	0,061		
	Salinispora pacifica CNT584	4912	3	0,061		
	Legionella moravica DSM 19234	3275	2	0,061		
	Burkholderia sp. UYPR1.413	9835	6	0,061		
	Marinococcus halotolerans DSM 16375	3299	2	0,061		
	Salinispora pacifica CNY239	4954	3	0,061		
	Gordonia soli NBRC 108243	4955	3	0,061		
	Streptomyces sviceus ATCC 29083	8275	5	0,060		
	Burkholderia bryophila 376MFSha3.1	6625	4	0,060		
	Paenibacillus pasadenensis DSM 19293	4970	3	0,060		
	Alicyclobacillus herbarius DSM 13609	3323	2	0,060		
	Gluconacetobacter hansenii ATCC 23769	3353	2	0,060		
	Ralstonia eutropha H16	6718	4	0,060		
	Hymenobacter norwichensis DSM 15439	5053	3	0,059		

	Streptomyces bingchenggensis BCW-1	10106	6	0,059		
	Rhizobium rhizogenes K84	6744	4	0,059		
	Crinalium epipsammum PCC 9333	5059	3	0,059		
	Herbaspirillum sp. CF444	5064	3	0,059		
	Streptomyces sp. 351MFTsu5.1	8466	5	0,059		
	Burkholderia thailandensis TXDOH	6776	4	0,059		
	Klebsiella sp. 1_1_55	5089	3	0,059		
	Bordetella petrii Se-1111R, DSM 12804	5092	3	0,059		
	Herminiimonas arsenicoxydans ULPAs1	3399	2	0,059		
	Fischerella sp. PCC 9339	6807	4	0,059		
	Flavobacteriaceae bacterium HQM9	3410	2	0,059		
	Verminephrobacter eiseniae EF01-2	5115	3	0,059		
	Burkholderia thailandensis Bt4	6824	4	0,059		
	Chromohalobacter salexigens 1H11, DSM 3043	3412	2	0,059		
	Aquiflexum balticum BA160, DSM 16537	5121	3	0,059		
	Sagittula stellata E-37	5121	3	0,059		
	Frankia sp. BCU110501	6839	4	0,058		
	Acinetobacter baylyi ADP1	3433	2	0,058		
	Salinispora arenicola CNX508	5152	3	0,058		

	Trichodesmium erythraeum IMS101	5156	3	0,058		
	Flexibacter roseolus DSM 9546	3442	2	0,058		
	Bacillus chagannorensis DSM 18086	3443	2	0,058		
	Calothrix sp. PCC 7103	10329	6	0,058		
	Nitrosococcus oceani AFC27	3445	2	0,058		
	Lentisphaera araneosa HTCC2155	5173	3	0,058		
	Amycolatopsis halophila YIM 93223, DSM 45216	5187	3	0,058		
	Salinispora arenicola CNX814	5188	3	0,058		
	Novosphingobium acidiphilum DSM 19966	3460	2	0,058		
	Salinispora arenicola CNY011	5192	3	0,058		
	Bordetella avium 197N	3464	2	0,058		
	Klebsiella variicola At-22	5213	3	0,058		
	Pseudorhodobacter ferrugineus DSM 5888	3480	2	0,057		
	Rhizobium gallicum bv. gallicum R602sp	6977	4	0,057		
	Aestuariibacter salexigens DSM 15300	3490	2	0,057		
	Jiangella gansuensis YIM 002, DSM 44835	5250	3	0,057		
	Deinococcus deserti VCD115	3511	2	0,057		
	Salinispora pacifica CNT084	5290	3	0,057		

	Salinispora arenicola CNH877	5299	3	0,057		
	Legionella longbeachae NSW150	3536	2	0,057		
	Streptomyces scabiei 87.22	8841	5	0,057		
	Halomonas elongata DSM 2581	3556	2	0,056		
	Acidobacteriaceae bacterium TAA 166	5364	3	0,056		
	Eudoraea adriatica DSM 19308	3579	2	0,056		
	Pseudomonas thermotolerans J53	3586	2	0,056		
	Rhizobium leguminosarum bv. viciae 248	7179	4	0,056		
	Microlunatus phosphovorus NM-1	5391	3	0,056		
	Salinispora pacifica CNY331	5391	3	0,056		
	Roseovarius nubinhibens ISM	3605	2	0,055		
	Burkholderia cenocepacia J2315	7229	4	0,055		
	Salinispora arenicola CNH964	5426	3	0,055		
	Caulobacter sp. AP07	5433	3	0,055		
	Burkholderia xenovorans LB400	9059	5	0,055		
	Burkholderia ubonensis Bu	7250	4	0,055		
	Frankia sp. EAN1pec	7250	4	0,055		
	Cucumibacter marinus DSM 18995	3628	2	0,055		
	Streptomyces sp. HrubLS-	7257	4	0,055		

	53					
	Pseudomonas thermotolerans DSM 14292	3630	2	0,055		
	Gluconacetobacter diazotrophicus PAl 5, DSM 5601	3633	2	0,055		
	Mycobacterium tuberculosis CCDC5180	3639	2	0,055		
	Leptospira biflexa Patoc 1 (Ames)	3641	2	0,055		
	Burkholderia pseudomallei 1655	5467	3	0,055		
	Empedobacter brevis ATCC 43319	3646	2	0,055		
	Catenulispora acidiphila ID139908, DSM 44928	9125	5	0,055		
	Pseudomonas putida PC9	5478	3	0,055		
	Salinispora arenicola CNP105	5478	3	0,055		
	Rhizobium sp. CF142	7306	4	0,055		
	Cyanothece sp. PCC 7425	5481	3	0,055		
	Pseudomonas putida KT2440	5481	3	0,055		
	Burkholderia pseudomallei 668	7322	4	0,055		
	Massilia alkalitolerans DSM 17462	5500	3	0,055		
	Methylobacterium sp. WSM2598	7349	4	0,054		
	Rhizobium leguminosarum bv. viciae 3841	7357	4	0,054		
	Burkholderia pseudomallei 1106b	5518	3	0,054		

	Burkholderia pseudomallei 1710a	5520	3	0,054		
	Gilvimarinus chinensis DSM 19667	3680	2	0,054		
	Mycobacterium tuberculosis CCDC5079	3695	2	0,054		
	Rhodococcus jostii RHA1	9242	5	0,054		
	Chitinophaga pinensis UQM 2034, DSM 2588	7396	4	0,054		
	Rhizobium leguminosarum bv viciae 128C53	7396	4	0,054		
	Acidiphilium cryptum JF-5	3701	2	0,054		
	Rhizobium leguminosarum bv. trifolii SRDI943	7406	4	0,054		
	Chitiniphilus shinanonensis DSM 23277	3713	2	0,054		
	Glaciecola nitratireducens FR1064	3720	2	0,054		
	Streptomyces sp. TAA204	5580	3	0,054		
	Klebsiella oxytoca KCTC 1686	5598	3	0,054		
	Rhizobium leguminosarum bv viciae WSM1481	7464	4	0,054		
	Rhizobium leguminosarum bv viciae VF39	7469	4	0,054		
	Mycobacterium tuberculosis RGTB327	3739	2	0,053		
	Rhizobium leguminosarum bv. trifolii WSM597	7481	4	0,053		
	Delftia sp. JGI 0001021-J09	3743	2	0,053		
	Polaromonas sp. JS666	5634	3	0,053		
	Burkholderia sp. H160	7515	4	0,053		

	Paenibacillus massiliensis DSM 16942	5637	3	0,053		
	Janthinobacterium sp. Marseille	3763	2	0,053		
	Oscillatoriales sp. JSC-1	5650	3	0,053		
	Burkholderia pseudomallei Pasteur	5652	3	0,053		
	Leptospira biflexa Patoc 1 (Paris)	3770	2	0,053		
	Burkholderia pseudomallei 406e	5672	3	0,053		
	Burkholderia phymatum STM815	7574	4	0,053		
	Thioflavicoccus mobilis 8321	3787	2	0,053		
	Mycobacterium gilvum PYR-GCK	5683	3	0,053		
	Singularimonas variicoloris DSM 15731	3793	2	0,053		
	Phaeobacter gallaeciensis 2.10	3798	2	0,053		
	Francisella novicida 3523	1902	1	0,053		
	Novosphingobium nitrogenifigens DSM 19370	3807	2	0,053		
	Hymenobacter aerophilus DSM 13606	3815	2	0,052		
	Rubritepida flocculans DSM 14296	3821	2	0,052		
	Kitasatospora setae KM-6054, NBRC 14216	7669	4	0,052		
	Pseudomonas aeruginosa PACS2	5753	3	0,052		
	Actinomycetospora chiangmaiensis DSM 45062	5756	3	0,052		

	Cytophaga hutchinsonii ATCC 33406	3843	2	0,052		
	Gordonia araii NBRC 100433	3845	2	0,052		
	Beijerinckia indica indica ATCC 9039	3850	2	0,052		
	Arenibacter latericius DSM 15913	3851	2	0,052		
	Achromobacter arsenitoxydans SY8	5778	3	0,052		
	Mycobacterium tuberculosis UT205	3852	2	0,052		
	Novosphingobium nitrogenifigens Y88, DSM 19370	3853	2	0,052		
	Pseudomonas sp. GM18	5797	3	0,052		
	Rhizobium leguminosarum bv. trifolii CC283b	7731	4	0,052		
	Thiothrix disciformis DSM 14473	3869	2	0,052		
	Legionella longbeachae D-4968	3879	2	0,052		
	Mycobacterium africanum GM041182	3880	2	0,052		
	Chryseobacterium caeni DSM 17710	3888	2	0,051		
	Micromonospora sp. CNB394	5836	3	0,051		
	Streptomyces avermitilis MA-4680	7792	4	0,051		
	alpha proteobacterium SCGC AAA298-K06	1951	1	0,051		
	Glaciecola pallidula DSM 14239	3902	2	0,051		

	Vibrio nigripulchritudo ATCC 27043	5855	3	0,051		
	Acidiphilium sp. PM, DSM 24941	3908	2	0,051		
	Mycobacterium canettii CIPT 140010059	3909	2	0,051		
	Frankia sp. EUN1f	7833	4	0,051		
	Klebsiella pneumoniae 342	5881	3	0,051		
	Cytophaga aurantiaca DSM 3654	3923	2	0,051		
	Ramlibacter tataouinensis TTB310	3926	2	0,051		
	Nocardiopsis sp. CNS639	5890	3	0,051		
	Niabella soli JS13-8, DSM 19437	3931	2	0,051		
	Prochlorococcus marinus MIT 9301	1967	1	0,051		
	Burkholderia cepacia GG4	5903	3	0,051		
	Pelagibacterium halotolerans B2	3940	2	0,051		
	Mycobacterium tuberculosis SUMu001	3942	2	0,051		
	Burkholderia mimosarum LMG 23256	7885	4	0,051		
	Cupriavidus necator N-1, ATCC 43291	7915	4	0,051		
	Phaeobacter gallaeciensis DSM 17395	3960	2	0,051		
	Pseudomonas fluorescens F113	5952	3	0,050		
	Oscillatoria sp. PCC 6506	5965	3	0,050		
	Streptomyces sp. W007	7969	4	0,050		

	Hoeflea sp. 108	5977	3	0,050		
	Gordonia rhizosphera NBRC 16068	5979	3	0,050		
	Phaeobacter inhibens T5, DSM 16374	3986	2	0,050		
	Alcaligenes faecalis phenolicus DSM 16503	3993	2	0,050		
	Mycobacterium bovis BCG Tokyo 172	3996	2	0,050		
	Mycobacterium tuberculosis CTRI-2	3996	2	0,050		
	Gluconacetobacter diazotrophicus PAl 5, DSM 5601	3997	2	0,050		
	Candidatus Solibacter usitatus Ellin6076	8003	4	0,050		
	Rhizobium etli Kim 5	6005	3	0,050		
	Acidiphilium multivorum AIU301	4004	2	0,050		
	Burkholderia cepacia UCB 717, ATCC 25416	8025	4	0,050		
	Mycobacterium bovis AF2122/97	4014	2	0,050		
	Pseudomonas aeruginosa LESB58	6026	3	0,050		
	Mycobacterium tuberculosis F11 (ExPEC)	4019	2	0,050		
	Mycobacterium tuberculosis KZN 605 (XDR)	4019	2	0,050		
	Rheinheimera sp. A13L	4019	2	0,050		
	Methyloferula stellata AR4	4027	2	0,050		
	Mycobacterium bovis BCG Mexico	4030	2	0,050		

	Mycobacterium vanbaalenii PYR-1	6047	3	0,050		
	Oceanobacter kriegii DSM 6294	4034	2	0,050		
	Klebsiella oxytoca E718	6056	3	0,050		
	Alteromonas sp. ALT199	4040	2	0,050		
	Streptomyces clavuligerus ATCC 27064	6060	3	0,050		
	Gemmata obscuriglobus UQM 2246	8086	4	0,049		
	Mycobacterium tuberculosis KZN 4207 (DS)	4043	2	0,049		
	Mycobacterium chubuense NBB4	6069	3	0,049		
	Mycobacterium bovis BCG Pasteur 1173P2	4048	2	0,049		
	Variovorax paradoxus EPS	6086	3	0,049		
	Mycobacterium tuberculosis H37Rv (lab strain)	4062	2	0,049		
	Rhizobium etli CIAT 652	6127	3	0,049		
	Nitrosococcus halophilus Nc4	4086	2	0,049		
	Mycobacterium tuberculosis C	4087	2	0,049		
	Burkholderia sp. KJ006	6143	3	0,049		
	Mycobacterium tuberculosis H37Ra	4099	2	0,049		
	Sciscionella marina DSM 45152	8212	4	0,049		
	Mycobacterium tuberculosis KZN 1435 (MDR)	4107	2	0,049		
	Pseudomonas brassicacearum	6176	3	0,049		

	brassicacearum NFM421					
	Prochlorococcus marinus MIT 9215	2059	1	0,049		
	Burkholderia sp. CCGE1003	6188	3	0,048		
	Roseibium sp. TrichSKD4	6202	3	0,048		
	Pseudomonas aeruginosa 2192	6203	3	0,048		
	Phaeobacter gallaeciensis BS107, CIP 105210	4136	2	0,048		
	Mycobacterium tuberculosis EAS054	4150	2	0,048		
	Burkholderia pseudomallei 14	8314	4	0,048		
	Mycobacterium tuberculosis 98-R604 INH-RIF-EM	4159	2	0,048		
	Mycobacterium tuberculosis GM 1503	4163	2	0,048		
	Mycobacterium tuberculosis H37Rv	4170	2	0,048		
	Pseudomonas fluorescens Pf-5	6257	3	0,048		
	Burkholderia pseudomallei 1026b	6262	3	0,048		
	Aquimarina muelleri DSM 19832	4179	2	0,048		
	Streptomyces sp. e14	6270	3	0,048		
	Mycobacterium tuberculosis 210	4182	2	0,048		
	Chryseobacterium sp. CF314	4183	2	0,048		
	Mycobacterium tuberculosis T46	4183	2	0,048		

	Mycobacterium tuberculosis CPHL_A	4187	2	0,048		
	Rheinheimera baltica DSM 14885	4187	2	0,048		
	Pseudomonas aeruginosa PA45	6282	3	0,048		
	Streptomyces sp. CNS615	6283	3	0,048		
	Mycobacterium tuberculosis KZN 4207	4192	2	0,048		
	Mycobacterium fortuitum fortuitum DSM 46621	6299	3	0,048		
	Paenibacillus lactis 154	6305	3	0,048		
	Echinicola pacifica DSM 19836	4206	2	0,048		
	Mycobacterium tuberculosis KZN V2475	4211	2	0,047		
	Streptomyces sp. CNT318	6320	3	0,047		
	Mycobacterium tuberculosis KZN R506	4219	2	0,047		
	Sphingomonas sp. LH128	6335	3	0,047		
	Burkholderia pseudomallei 354e	6348	3	0,047		
	Mycobacterium tuberculosis SUMu007	4232	2	0,047		
	Pseudomonas stutzeri A1501	4237	2	0,047		
	Pseudomonas aeruginosa NCGM2.S1	6358	3	0,047		
	Micromonospora aurantiaca ATCC 27029	6360	3	0,047		
	Mycobacterium tuberculosis SUMu006	4241	2	0,047		

	Acaryochloris marina MBIC11017	8488	4	0,047		
	Dinoroseobacter shibae DFL-12, DSM 16493	4244	2	0,047		
	Nitratireductor pacificus pht-3B	4246	2	0,047		
	Mycobacterium tuberculosis SUMu009	4248	2	0,047		
	Mycobacterium tuberculosis K85	4249	2	0,047		
	Mycobacterium tuberculosis SUMu002	4250	2	0,047		
	Mycobacterium tuberculosis T85	4251	2	0,047		
	Burkholderia sp. WSM2232	6383	3	0,047		
	Mycobacterium tuberculosis SUMu005	4258	2	0,047		
	Mycobacterium tuberculosis SUMu008	4261	2	0,047		
	Mycobacterium tuberculosis SUMu011	4267	2	0,047		
	Neptunomonas japonica DSM 18939	4271	2	0,047		
	Polynucleobacter necessarius asymbioticus QLW-P1DMWA-1	2136	1	0,047		
	Saccharomonospora saliphila YIM 90502	4272	2	0,047		
	Bradyrhizobium sp. Cp5.3	8545	4	0,047		
	Methylopila sp. M107	4275	2	0,047		
	Mycobacterium tuberculosis SUMu010	4280	2	0,047		
	Mycobacterium tuberculosis	4282	2	0,047		

	94_M4241A					
	Mycobacterium tuberculosis SUMu004	4283	2	0,047		
	Paracoccus sp. N5	4284	2	0,047		
	Chamaesiphon minutus PCC 6605	6427	3	0,047		
	Pseudomonas stutzeri Lautrop AB 201, ATCC 17588	4287	2	0,047		
	Mycobacterium tuberculosis SUMu003	4290	2	0,047		
	Burkholderia pseudomallei 1710b	6436	3	0,047		
	Mycobacterium tuberculosis CDC1551	4300	2	0,047		
	Mycobacterium tuberculosis T17	4300	2	0,047		
	Mycobacterium tuberculosis T92	4300	2	0,047		
	Bacteroidetes bacterium SCGC AAA027-N21	2153	1	0,046		
	Teredinibacter turnerae T7901	4308	2	0,046		
	Nocardiopsis sp. CNT312	4311	2	0,046		
	Pseudomonas aeruginosa 39016	6471	3	0,046		
	Marinobacterium litorale DSM 23545	4315	2	0,046		
	Mycobacterium tuberculosis 02_1987	4318	2	0,046		
	Rickettsia endosymbiont of Ixodes scapularis	2160	1	0,046		
	Mycobacterium tuberculosis	4322	2	0,046		

	SUMu012					
	Rhodococcus opacus M213	8680	4	0,046		
	Marinobacter aquaeolei VT8	4342	2	0,046		
	Nitratifractor salsuginis E9I37-1, DSM 16511	2173	1	0,046		
	Streptomyces sp. SPB78	6544	3	0,046		
	Arthrobacter sp. 161MFSha2.1	4363	2	0,046		
	Streptomyces sp. 142MFCol3.1	8727	4	0,046		
	Halomonas lutea DSM 23508	4368	2	0,046		
	Pseudomonas syringae Cit 7 (Cit7)	6553	3	0,046		
	Planctomyces limnophilus Mu 290, DSM 3776	4372	2	0,046		
	Rhizobium leguminosarum bv. trifolii CB782	6559	3	0,046		
	Pseudomonas stutzeri AN10, CCUG 29243	4374	2	0,046		
	Pseudomonas stutzeri CMT.A.9, DSM 4166	4374	2	0,046		
	Mycobacterium tuberculosis Haarlem	4376	2	0,046		
	Pedosphaera parvula Ellin514	6573	3	0,046		
	Mycobacterium sp. JDM601	4398	2	0,045		
	Pseudomonas stutzeri RCH2	4412	2	0,045		
	Burkholderia glumae AU6208	4418	2	0,045		
	Ralstonia solanacearum	4418	2	0,045		

	UW551					
	Saccharomonospora xinjiangensis XJ-54	4419	2	0,045		
	Roseobacter sp. R2A57	4432	2	0,045		
	Alteromonas taeanensis SN2	4442	2	0,045		
	Bordetella parapertussis 12822	4447	2	0,045		
	Pseudonocardia sp. P1	6674	3	0,045		
	Indibacter alkaliphilus LW1 (Draft1)	4451	2	0,045		
	Acidothermus cellulolyticus 11B	2229	1	0,045		
	Streptomyces sp. CNH189	6689	3	0,045		
	Streptomyces sp. CNT360	6690	3	0,045		
	Rhizobium giardinii bv. giardinii H152	6691	3	0,045		
	Bradyrhizobium sp. STM 3809	6699	3	0,045		
	Marinobacter adhaerens HP15, DSM 23420	4470	2	0,045		
	Mesorhizobium ciceri CMG6	6718	3	0,045		
	Burkholderia multivorans CGD2M	6730	3	0,045		
	Streptomyces sp. Tu6071	6733	3	0,045		
	Mycobacterium tuberculosis H37Ra	4489	2	0,045		
	Burkholderia multivorans CGD2	6734	3	0,045		
	Solitalea canadensis USAM 9D, DSM 3403	4490	2	0,045		

	Burkholderia cepacia AMMD	6738	3	0,045		
	Cylindrospermum stagnale PCC 7417	6739	3	0,045		
	Mycobacterium smegmatis MC2 155	6745	3	0,044		
	Arthrobacter castelli DSM 16402	4516	2	0,044		
	Kaistia granuli DSM 23481	4516	2	0,044		
	Bacillus aidingensis DSM 18341	4530	2	0,044		
	Saccharomonospora azurea NA-128, DSM 44631	4530	2	0,044		
	Burkholderia graminis C4D1M	6798	3	0,044		
	Rhizobium leguminosarum bv trifolii WSM1689	6798	3	0,044		
	Shewanella sp. ANA-3	4537	2	0,044		
	Photorhabdus asymbiotica asymbiotica ATCC 43949	4555	2	0,044		
	Rhizobium leguminosarum bv trifolii SRDI565	6836	3	0,044		
	Bradyrhizobium elkanii USDA 94	9129	4	0,044		
	Candidatus Glomeribacter sp. 1016415	2284	1	0,044		
	gamma proteobacterium IMCC2047	2285	1	0,044		
	Pseudomonas sp. Chol1	4578	2	0,044		
	Cycloclasticus sp. P1	2292	1	0,044		
	Burkholderia ambifaria MC40-6	6878	3	0,044		
	Colwellia piezophila ATCC	4598	2	0,043		

	BAA-637					
	Kaistia adipata DSM 17808	4601	2	0,043		
	Pseudomonas stutzeri KOS6	4603	2	0,043		
	Saccharomonospora azurea SZMC 14600	4604	2	0,043		
	Streptomyces griseoaurantiacus M045	6910	3	0,043		
	Adhaeribacter aquaticus MBRG1.5, DSM 16391	4607	2	0,043		
	Frankia sp. CcI3	4621	2	0,043		
	Sphingobacterium spiritivorum ATCC 33861	4623	2	0,043		
	Mycobacterium smegmatis MC2 155	6941	3	0,043		
	Rhizobium leguminosarum bv viciae UPM1131	6951	3	0,043		
	Terracoccus sp. 273MFTsu3.1	4641	2	0,043		
	Methylobacter tundripaludum SV96	4653	2	0,043		
	Leptolyngbya sp. PCC 7376	4654	2	0,043		
	Cycloclasticus pugetii PS-1	2332	1	0,043		
	Prochlorococcus marinus MIT 9313	2334	1	0,043		
	Variovorax paradoxus 110B	7002	3	0,043		
	Mesorhizobium loti R88b	7016	3	0,043		
	Echinicola vietnamensis KMM 6221, DSM 17526	4684	2	0,043		
	Burkholderia oklahomensis C6786	7034	3	0,043		

	Marmoricola sp. URHB0036	4705	2	0,043		
	Pseudonocardia dioxanivorans CB1190	7071	3	0,042		
	Herbaspirillum sp. GW103	4717	2	0,042		
	Photobacterium sp. SKA34	4734	2	0,042		
	Comamonadaceae bacterium URHA0028	4737	2	0,042		
	Gordonia amarae NBRC 15530	4748	2	0,042		
	Photobacterium angustum S14	4751	2	0,042		
	Bradyrhizobium sp. ORS 375	7143	3	0,042		
	Rhizobium leguminosarum bv. trifolii WSM2012	7166	3	0,042		
	Mycobacterium tusciae JS617	7168	3	0,042		
	Streptomyces sp. CNH099	7172	3	0,042		
	Nitratireductor aquibiodomus RA22 (Draft1)	4786	2	0,042		
	Salinispora pacifica CNT124	4796	2	0,042		
	Candidatus Regiella insecticola R5.15	2399	1	0,042		
	Streptomyces griseoflavus Tu4000	7207	3	0,042		
	Streptomyces griseus griseus NBRC 13350	7222	3	0,042		
	Burkholderia pseudomallei BPC006	7230	3	0,041		
	Mucilaginibacter paludis TPT56, DSM 18603	7236	3	0,041		
	Mycobacterium abscessus	4833	2	0,041		

	4S-0116-R					
	Salinispora pacifica CNT855	4834	2	0,041		
	Rhizobium leguminosarum bv. viciae TOM	7255	3	0,041		
	Frankia sp. EuI1c	7262	3	0,041		
	Ralstonia sp. 5_7_47FAA	4843	2	0,041		
	Deinococcus aquatilis DSM 23025	4845	2	0,041		
	Acidovorax avenae avenae ATCC 19860	4850	2	0,041		
	Sorangium cellulosum So ce 56	9700	4	0,041		
	Methylomonas sp. MK1	4851	2	0,041		
	Burkholderia pseudomallei 1106a	7278	3	0,041		
	Acidovorax sp. CF316	7281	3	0,041		
	Salinispora pacifica CNT569	4856	2	0,041		
	Burkholderia oklahomensis EO147	7289	3	0,041		
	Rhizobium leguminosarum bv. trifolii WSM1325	7292	3	0,041		
	Flavobacterium sp. SCGC AAA160-P02	2432	1	0,041		
	Salinispora pacifica CNS237	4865	2	0,041		
	Rhizobium leguminosarum bv viciae GB30	7302	3	0,041		
	Mycobacterium massiliense CCUG 48898	4878	2	0,041		
	Mycobacterium abscessus 4S-0206	4889	2	0,041		
	Mycobacterium abscessus	4894	2	0,041		

	4S-0116-S					
	Mycobacterium abscessus 4S-0726-RA	4894	2	0,041		
	Leisingera nanhaiensis NH52F, DSM 24252 (scaffold version)	4896	2	0,041		
	Mycobacterium abscessus 4S-0303	4898	2	0,041		
	Salinispora pacifica CNT150	4898	2	0,041		
	Mycobacterium abscessus 4S-0726-RB	4902	2	0,041		
	Rhizobium leguminosarum bv. viciae Ps8	7359	3	0,041		
	Marinomonas sp. MED121	4918	2	0,041		
	Streptomyces clavuligerus ATCC 27064	7381	3	0,041		
	Mesorhizobium loti CJ3sym	7401	3	0,041		
	Rhodopirellula baltica SH 1	7414	3	0,040		
	Mycobacterium abscessus M93	4955	2	0,040		
	Verrucomicrobiales sp. DG1235	4957	2	0,040		
	Salinispora pacifica CNT138	4958	2	0,040		
	Burkholderia pseudomallei MSHR305	7440	3	0,040		
	Granulicella mallensis MP5ACTX8	4960	2	0,040		
	Streptomyces griseus XyelbKG-1 1	7450	3	0,040		
	Burkholderia pseudomallei 576	7454	3	0,040		
	Myxococcus xanthus DK	7454	3	0,040		

	1622					
	Salinispora arenicola CNT800	4974	2	0,040		
	Rhizobium leguminosarum bv viciae UPM1137	7462	3	0,040		
	Salinispora pacifica DSM 45547	4981	2	0,040		
	Mycobacterium abscessus CIP 104536	4991	2	0,040		
	Burkholderia phytofirmans PsJN	7487	3	0,040		
	Salinispora arenicola CNR921	4992	2	0,040		
	Gordonia otitidis NBRC 100426	4993	2	0,040		
	Cupriavidus necator HPC(L)	4995	2	0,040		
	Rhizobium leguminosarum bv viciae WSM1455	7494	3	0,040		
	Ralstonia sp. 5_2_56FAA	5005	2	0,040		
	Salinispora pacifica DSM 45546	5005	2	0,040		
	Salinispora arenicola CNH962	5012	2	0,040		
	Salinispora arenicola CNH963	5017	2	0,040		
	Burkholderiales sp. JOSHI_001	5022	2	0,040		
	Herbaspirillum sp. YR522	5025	2	0,040		
	Streptomyces venezuelae Shinobu 719, ATCC 10712	7541	3	0,040		
	Rhodopseudomonas palustris BisB18	5028	2	0,040		

	Burkholderia pseudomallei Pakistan 9	7552	3	0,040		
	Pseudomonas mendocina NK-01	5035	2	0,040		
	Salinispora pacifica CNT854	5035	2	0,040		
	Salinispora pacifica CNS860	5040	2	0,040		
	Stanieria cyanosphaera PCC 7437	5041	2	0,040		
	Bacillus kribbensis DSM 17871	5042	2	0,040		
	Sulfurovum sp. NBC37-1	2525	1	0,040		
	Leptospirillum ferriphilum ML-04	2527	1	0,040		
	Acaryochloris sp CCMEE 5410	7587	3	0,040		
	Actinoplanes globisporus DSM 43857	10116	4	0,040		
	Salinispora pacifica DSM 45549	5060	2	0,040		
	Mycobacterium abscessus M94	5063	2	0,040		
	Salinispora pacifica CNT131	5064	2	0,039		
	Colwellia psychrerythraea 34H	5066	2	0,039		
	Rhizobium leguminosarum bv. viciae Vc2	7602	3	0,039		
	Salinispora arenicola CNT850	5070	2	0,039		
	Ralstonia solanacearum Po82	5080	2	0,039		
	Rhodopseudomonas palustris DX-1	5081	2	0,039		

	Bordetella bronchiseptica RB50	5086	2	0,039		
	Methylomonas sp. 11b	5086	2	0,039		
	Salinispora pacifica CNR942	5086	2	0,039		
	Rhizobium etli Brasil 5	5088	2	0,039		
	Salinispora arenicola CNX481	5088	2	0,039		
	Nesterenkonia sp. F	2545	1	0,039		
	Streptomyces lividans TK24	7636	3	0,039		
	Pseudonocardia asaccharolytica DSM 44247	5091	2	0,039		
	Rhizobium leguminosarum bv. viciae Vh3	7647	3	0,039		
	Streptomyces viridosporus T7A, ATCC 39115	7648	3	0,039		
	Flavobacterium johnsoniae UW101, ATCC 17061	5099	2	0,039		
	Bradyrhizobium elkanii WSM1741	7649	3	0,039		
	Burkholderia pseudomallei MSHR346	7657	3	0,039		
	Salinispora pacifica CNT003	5106	2	0,039		
	Myxococcus xanthus DZ2	7662	3	0,039		
	Salinispora pacifica DSM 45543	5120	2	0,039		
	Simkania negevensis Z, ATCC VR-1471	2560	1	0,039		
	Streptomyces clavuligerus ATCC 27064	7680	3	0,039		
	Salinispora pacifica CNS863	5125	2	0,039		
	Rhizobium etli 8C-3	5131	2	0,039		

	Salinispora arenicola CNT798	5135	2	0,039		
	Caulobacter sp. URHA0033	5142	2	0,039		
	Rhodococcus imtechensis RKJ300	7733	3	0,039		
	Salinispora arenicola CNR425	5160	2	0,039		
	Salinispora arenicola CNS-205	5169	2	0,039		
	Chroococcidiopsis sp. PCC 6712	5176	2	0,039		
	Pedobacter sp. BAL39	5176	2	0,039		
	Salinispora arenicola CNQ748	5179	2	0,039		
	Gordonia terrae NBRC 100016	5181	2	0,039		
	Salinispora arenicola CNS299	5181	2	0,039		
	Gordonia polyisoprenivorans VH2, DSM 44266	5188	2	0,039		
	Saccharomonospora cyanea NA-134	5196	2	0,038		
	Salinispora pacifica CNT849	5196	2	0,038		
	Ralstonia solanacearum GMI1000	5204	2	0,038		
	Salinispora arenicola CNR107	5209	2	0,038		
	Gordonia sp. KTR9	5217	2	0,038		
	Rhizobium leguminosarum bv trifolii WSM2297	7827	3	0,038		
	Salinispora tropica CNH898	5219	2	0,038		
	Mycobacterium abscessus	5224	2	0,038		

	6G-0125-R					
	Streptomyces ghanaensis ATCC 14672	7840	3	0,038		
	Sporocytophaga myxococcoides DSM 11118	5236	2	0,038		
	Mycobacterium abscessus 6G-0212	5237	2	0,038		
	Burkholderia vietnamiensis G4	7880	3	0,038		
	Salinispora arenicola CNY260	5256	2	0,038		
	Salinispora arenicola CNX482	5260	2	0,038		
	Klebsiella pneumoniae KCTC 2242	5264	2	0,038		
	Yaniella halotolerans DSM 15476	2633	1	0,038		
	Microchaete sp. PCC 7126	5278	2	0,038		
	Mycobacterium colombiense CECT 3035	5279	2	0,038		
	Sporichthya polymorpha DSM 43042	5279	2	0,038		
	Vibrio tubiashii ATCC 19109	5280	2	0,038		
	Salinispora tropica CNB536	5287	2	0,038		
	Pseudomonas putida W619	5292	2	0,038		
	Pseudomonas entomophila L48	5293	2	0,038		
	Pseudomonas syringae pv. syringae SM	5293	2	0,038		
	Salinispora arenicola CNY256	5301	2	0,038		
	Gloeocapsa sp. PCC 7428	5304	2	0,038		

	Ralstonia pickettii OR214	5313	2	0,038		
	Pseudomonas syringae pv. syringae 642	5318	2	0,038		
	Burkholderia pseudomallei BCC215	7993	3	0,038		
	Salinispora arenicola CNH941	5340	2	0,037		
	Salinispora arenicola CNB458	5343	2	0,037		
	Klebsiella oxytoca M5al	5349	2	0,037		
	Salinispora arenicola CNP193	5357	2	0,037		
	Mycobacterium abscessus 3A-0122-S	5359	2	0,037		
	Pseudomonas sp. FGI182	5365	2	0,037		
	Chryseobacterium gleum F93, ATCC 35910	5371	2	0,037		
	Klebsiella pneumoniae pneumoniae MGH78578	5377	2	0,037		
	Kytococcus sedentarius 541, DSM 20547	2692	1	0,037		
	Pseudomonas sp. GM84	5384	2	0,037		
	Zooshikella ganghwensis DSM 15267	5387	2	0,037		
	Glycomyces tenuis DSM 44171	5399	2	0,037		
	Salinispora pacifica CNT045	5399	2	0,037		
	Mycobacterium abscessus 5S-1215	5402	2	0,037		
	Mycobacterium abscessus 3A-0119-R	5403	2	0,037		
	Lewinella persica DSM	5405	2	0,037		

	23188					
	Burkholderia pseudomallei 112	8109	3	0,037		
	Myxococcus stipitatus DSM 14675	8128	3	0,037		
	Salinispora arenicola CNS673	5421	2	0,037		
	Lautropia mirabilis ATCC 51599	2713	1	0,037		
	Mycobacterium abscessus 3A-0930-S	5434	2	0,037		
	Mycobacterium gilvum Spyr1	5434	2	0,037		
	Duganella zoogloeoides ATCC 25935	5437	2	0,037		
	Mycobacterium abscessus 5S-1212	5437	2	0,037		
	Mycobacterium abscessus 5S-0421	5438	2	0,037		
	Mycobacterium abscessus 5S-0304	5440	2	0,037		
	Mycobacterium abscessus 5S-0708	5440	2	0,037		
	Rhizobium etli GR56	5440	2	0,037		
	Burkholderia pseudomallei NCTC 13177	8161	3	0,037		
	Pseudanabaena sp. PCC 6802	5447	2	0,037		
	Bradyrhizobium sp. TV2a.2	8174	3	0,037		
	Burkholderia pseudomallei B7210	8176	3	0,037		
	Mycobacterium abscessus 3A-0930-R	5458	2	0,037		

	Mycobacterium abscessus 5S-0921	5458	2	0,037		
	Pseudomonas syringae USA011 (USA011)	5477	2	0,037		
	Mycobacterium abscessus 3A-0731	5479	2	0,037		
	Gluconobacter oxydans 621H	2742	1	0,036		
	Burkholderia dolosa AUO158	5493	2	0,036		
	Mycobacterium marinum M, ATCC BAA-535	5501	2	0,036		
	Mycobacterium abscessus 6G-0728-S	5504	2	0,036		
	Thioalkalivibrio sp. AKL19	2752	1	0,036		
	Mycobacterium abscessus 6G-0728-R	5505	2	0,036		
	Salinispora arenicola CNB527	5508	2	0,036		
	Mycobacterium abscessus 6G-1108	5511	2	0,036		
	Mycobacterium abscessus 6G-0125-S	5512	2	0,036		
	Pseudomonas putida GB-1	5515	2	0,036		
	Mycobacterium abscessus 5S-0422	5519	2	0,036		
	Burkholderia pseudomallei 9	8281	3	0,036		
	Salinispora pacifica CNR114	5521	2	0,036		
	Prochlorococcus sp. CC9605	2761	1	0,036		
	Bradyrhizobium sp. ARR65	8310	3	0,036		
	Salinicoccus albus DSM	2770	1	0,036		

	19776					
	Rhizobium etli CIAT 894	5541	2	0,036		
	Streptomyces coelicolor A3(2)	8325	3	0,036		
	Bradyrhizobium elkanii USDA 3259	8327	3	0,036		
	Microcoleus chthonoplastes PCC 7420	8359	3	0,036		
	Streptomyces vitaminophilus DSM 41686	5575	2	0,036		
	Burkholderia pseudomallei 7894	8370	3	0,036		
	Burkholderia pseudomallei 91	8390	3	0,036		
	Salimicrobium sp. MJ3	2801	1	0,036		
	Nocardiopsis alba ATCC BAA-2165	5609	2	0,036		
	Bradyrhizobium japonicum WSM2793	8414	3	0,036		
	Leptolyngbya sp. PCC 7375	8448	3	0,036		
	Alcanivorax borkumensis SK2	2817	1	0,035		
	Bradyrhizobium sp. WSM3983	8456	3	0,035		
	Flavobacterium suncheonense DSM 17707	2821	1	0,035		
	Jonesia quinghaiensis DSM 15701	2823	1	0,035		
	Nocardiopsis dassonvillei dassonvillei DSM 43111	5647	2	0,035		
	Methylobacillus flagellatus KT	2824	1	0,035		

	Streptomyces sp. 303MFCol5.2	8480	3	0,035		
	Streptomyces sp. CNT372	5674	2	0,035		
	Gordonia polyisoprenivorans NBRC 16320	5686	2	0,035		
	Paenibacillus dendritiformis C454	5690	2	0,035		
	Bradyrhizobium elkanii USDA 3254	8564	3	0,035		
	Pseudomonas syringae pv. aceris M302273PT (Aceris)	5717	2	0,035		
	Rhizobium leguminosarum bv trifolii TA1	8576	3	0,035		
	Bradyrhizobium sp. WSM2254	8585	3	0,035		
	Photobacterium profundum 3TCK	5728	2	0,035		
	Variovorax sp. CF313	5732	2	0,035		
	Bradyrhizobium sp. CCGE-LA001	8603	3	0,035		
	Pseudomonas syringae CC1466 (CC1466)	5738	2	0,035		
	Actinomadura flavalba DSM 45200	5740	2	0,035		
	Salipiger mucosus DSM 16094 (scaffold version)	5745	2	0,035		
	Burkholderia pseudomallei DM98	8639	3	0,035		
	Cryptosporangium arvum YU 629-21, DSM 44712	8650	3	0,035		
	Rhizobium leguminosarum bv trifolii CC278f	8665	3	0,035		
	Oceanicaulis alexandrii DSM	2889	1	0,035		

	11625					
	Pseudomonas syringae CC1557 (CC1557)	5792	2	0,035		
	Oscillatoria formosa PCC 6407	5797	2	0,035		
	Synechococcus sp. JA-3-3Ab	2900	1	0,034		
	Porphyrobacter cryptus DSM 12079	2903	1	0,034		
	Streptomyces sp. SPB74	5808	2	0,034		
	Pseudomonas fluorescens HK44 (Draft 1)	5809	2	0,034		
	Rhizobium sp. JGI 0001003-A11	2911	1	0,034		
	Bradyrhizobium japonicum USDA 6	8736	3	0,034		
	Mycobacterium sp. 360MFTsu5.1	5833	2	0,034		
	Pseudomonas syringae CC457 (CC457)	5837	2	0,034		
	Tistrella mobilis KA081020-065	5851	2	0,034		
	Mycobacterium sp. JLS	5855	2	0,034		
	Pseudomonas fluorescens Pf0-1	5857	2	0,034		
	Burkholderia sp. JPY347	5878	2	0,034		
	Octadecabacter arcticus 238, DSM 13978	5883	2	0,034		
	Verrucomicrobia bacterium SCGC AAA168-E21 (genbank_version)	2943	1	0,034		
	Verrucomicrobia bacterium SCGC AAA168-F10	2943	1	0,034		

	(genbank_version)					
	Alkalilimnicola ehrlichei MLHE-1	2947	1	0,034		
	Synechococcus sp. JA-2-3B'a(2-13)	2947	1	0,034		
	Burkholderia nodosa DSM 21604	8860	3	0,034		
	Bradyrhizobium japonicum USDA 6	8886	3	0,034		
	Cupriavidus taiwanensis STM6018	5925	2	0,034		
	Frankia sp. DC12	5933	2	0,034		
	Pseudomonas sp. URMO17WK12:I6	5967	2	0,034		
	Erythrobacter sp, SD-21	2992	1	0,033		
	Cupriavidus taiwanensis LMG 19424	5986	2	0,033		
	Paenibacillus vortex V453	5993	2	0,033		
	Ulvibacter sp. SCB49	2999	1	0,033		
	Conexibacter woesei ID131577, DSM 14684	6001	2	0,033		
	Pantoea sp. At-9b	6007	2	0,033		
	Pseudomonas sp. 45MFCol3.1	6032	2	0,033		
	Chroococcidiopsis thermalis PCC 7203	6033	2	0,033		
	Pseudomonas mandelii 36MFCvi1.1	6059	2	0,033		
	Azospirillum halopraeferens DSM 3675	6075	2	0,033		
	Mesorhizobium australicum WSM2073	6080	2	0,033		

	Gramella portivictoriae DSM 23547	3045	1	0,033		
	Sideroxydans lithotrophicus ES-1	3049	1	0,033		
	Bradyrhizobium elkanii USDA 76	9151	3	0,033		
	Oscillatoria acuminata PCC 6304	6101	2	0,033		
	Rhizobium etli CFN 42, DSM 11541	6113	2	0,033		
	Raphidiopsis brookii D9	3057	1	0,033		
	Pseudoxanthomonas suwonensis J47	3066	1	0,033		
	Pseudomonas sp. GM79	6146	2	0,033		
	Oceanicaulis alexandrii HTCC2633	3081	1	0,032		
	Flavobacterium branchiophilum FL-15	3082	1	0,032		
	Bradyrhizobium japonicum USDA 38	9258	3	0,032		
	Pseudomonas aeruginosa PADK2_CF510	6183	2	0,032		
	Burkholderia multivorans ATCC 17616	6193	2	0,032		
	Rhizobium sp. AP16	6205	2	0,032		
	Candidatus Chloracidobacterium thermophilum B	3103	1	0,032		
	Streptomyces sp. TAA486	6224	2	0,032		
	Streptomyces sp. CNH287	6228	2	0,032		
	Mesorhizobium sp. URHA0056	6232	2	0,032		

	Loktanella vestfoldensis SKA53	3117	1	0,032		
	Streptomyces sp. CNT302	6237	2	0,032		
	Oxalobacteraceae bacterium AB_14	6242	2	0,032		
	Anabaena cylindrica PCC 7122	6258	2	0,032		
	Ensifer meliloti 1021	6295	2	0,032		
	Gordonia shandongensis DSM 45094	3159	1	0,032		
	Mobilicoccus pelagius NBRC 104925	3162	1	0,032		
	Streptosporangium roseum NI 9100, DSM 43021	9510	3	0,032		
	Mycobacterium rhodesiae NBB3	6342	2	0,032		
	Actinomadura atramentaria DSM 43919	6345	2	0,032		
	Burkholderia multivorans ATCC 17616	6373	2	0,031		
	Ensifer meliloti Mlalz-1	6388	2	0,031		
	Labrenzia aggregata IAM 12614	6390	2	0,031		
	Methylobacterium sp. 285MFTsu5.1	6392	2	0,031		
	Pseudoxanthomonas suwonensis J42	3197	1	0,031		
	Ensifer meliloti WSM1022	6398	2	0,031		
	Mesorhizobium loti R7A	6398	2	0,031		
	Pseudoxanthomonas spadix BD-a59	3202	1	0,031		
	Oceanimonas smirnovii	3210	1	0,031		

	ATCC BAA-899					
	Ensifer meliloti 4H41	6422	2	0,031		
	Cupriavidus metallidurans CH34	6430	2	0,031		
	Nitrobacter winogradskyi Nb-255	3215	1	0,031		
	Methylophaga aminisulfidivorans MP, KCTC 12909	3217	1	0,031		
	Longispora albida DSM 44784	6439	2	0,031		
	Arcobacter nitrofigilis DSM 7299	3220	1	0,031		
	Streptomyces sp. CNR698	6455	2	0,031		
	Nodosilinea nodulosa PCC 7104	6470	2	0,031		
	Bradyrhizobium sp. EC3.3	9710	3	0,031		
	Ignavibacterium album Mat9-16, JCM 16511	3243	1	0,031		
	Pseudomonas fluorescens SBW25	6492	2	0,031		
	Rhizobium tropici CIAT899	6494	2	0,031		
	Burkholderia sp. URHA0054	6499	2	0,031		
	Ensifer meliloti CIAM1775	6516	2	0,031		
	Chitinibacter tainanensis DSM 15459	3265	1	0,031		
	Mesorhizobium ciceri bv biserrulae WSM1271	6531	2	0,031		
	Haloglycomyces albus DSM 45210	3266	1	0,031		
	Frankia sp. QA3	6546	2	0,031		

	Ensifer sp. USDA 6670, CC2017	6547	2	0,031		
	Rhizobium tropici USDA 9039	6564	2	0,030		
	Citromicrobium bathyomarinum JL354	3283	1	0,030		
	Limnobacter sp. MED105	3285	1	0,030		
	Frateuria aurantia Kondo 67, DSM 6220	3288	1	0,030		
	Tolumonas auensis TA 4, DSM 9187	3288	1	0,030		
	Bacteriovorax marinus SJ	3291	1	0,030		
	Variovorax paradoxus 4MFCol3.1	6601	2	0,030		
	Burkholderia phenoliruptrix BR3459	6605	2	0,030		
	Bradyrhizobium sp. th.b2	9917	3	0,030		
	Pseudomonas sp. Ag1	6623	2	0,030		
	Rhizobium leguminosarum bv. trifolii WSM2304	6643	2	0,030		
	Burkholderia multivorans CGD1	6646	2	0,030		
	Streptomyces sp. SirexAA-E	6647	2	0,030		
	Sediminibacterium sp. OR43	3327	1	0,030		
	Mesorhizobium ciceri WSM4083	6662	2	0,030		
	Sediminibacterium sp. OR53	3332	1	0,030		
	Oceanimonas sp. GK1	3333	1	0,030		
	Halomonas sp. GFAJ-1	3347	1	0,030		
	Deinococcus maricopensis LB-34, DSM 21211	3367	1	0,030		

	Ralstonia solanacearum CFBP2957	3367	1	0,030		
	Mesorhizobium opportunistum WSM2075 (final version from ORNL)	6747	2	0,030		
	Alcanivorax sp. DG881	3384	1	0,030		
	Ensifer meliloti BL225C	6769	2	0,030		
	Ensifer meliloti RRl128	6770	2	0,030		
	Acinetobacter sp. ATCC 27244	3392	1	0,029		
	Mesorhizobium loti USDA 3471	6785	2	0,029		
	Nostoc punctiforme PCC 73102	6791	2	0,029		
	Oceanospirillum maris DSM 6286	3397	1	0,029		
	Kyrpidia tusciae T2, DSM 2912	3401	1	0,029		
	Ensifer meliloti GVPV12	6806	2	0,029		
	Synechococcus sp. WH5701	3403	1	0,029		
	Bradyrhizobium sp. URHA0002	6819	2	0,029		
	Bradyrhizobium sp. ORS278	6825	2	0,029		
	Ensifer meliloti GR4	6826	2	0,029		
	Psychroflexus torquis ATCC 700755	6835	2	0,029		
	Bradyrhizobium sp. ORS285	6842	2	0,029		
	Pseudomonas syringae pv. lachrymans 107 MAFF301315 (Lac 107)	6847	2	0,029		
	Gracilimonas tropica DSM 19535	3426	1	0,029		

	Porphyra umbilicalis P.um.1-endophyte07873 (Porphyra_umbilicalis_P.um.1-endophyte07873)	3437	1	0,029		
	Cyanobium gracile PCC 6307	3439	1	0,029		
	Ensifer meliloti CCNWSX0020	6900	2	0,029		
	Agromyces italicus DSM 16388	3451	1	0,029		
	Ensifer meliloti BO21CC	6907	2	0,029		
	Haliscomenobacter hydrossis O, DSM 1100	6918	2	0,029		
	Methylohalobius crimeensis 10Ki	3464	1	0,029		
	Streptomyces pristinaespiralis ATCC 25486	6937	2	0,029		
	Mesorhizobium sp. WSM3224	6951	2	0,029		
	Streptomyces roseosporus NRRL 15998	6986	2	0,029		
	Flavobacteriales sp. ALC-1	3502	1	0,029		
	Ensifer meliloti AK58	7013	2	0,029		
	Ensifer meliloti AK83, DSM 23913	7022	2	0,028		
	Maritalea myrionectae DSM 19524	3512	1	0,028		
	Bordetella pertussis CS	3516	1	0,028		
	Cyanothece sp. PCC 7822	7041	2	0,028		
	Fodinicurvata sediminis DSM 21159	3551	1	0,028		
	Amphritea japonica ATCC	3558	1	0,028		

	BAA-1530					
	Mycobacterium rhodesiae JS60	7116	2	0,028		
	Renibacterium salmoninarum ATCC 33209	3558	1	0,028		
	Acinetobacter haemolyticus ATCC 19194	3560	1	0,028		
	Chitinilyticum aquatile DSM 21506	3561	1	0,028		
	Methylobacterium sp. 4-46	7125	2	0,028		
	Owenweeksia hongkongensis DSM 17368	3563	1	0,028		
	Cupriavidus sp. AMP6	7130	2	0,028		
	Segniliparus rugosus ATCC BAA-974	3565	1	0,028		
	Beggiatoa alba B18LD	3569	1	0,028		
	Streptomyces roseosporus NRRL 11379	7141	2	0,028		
	Hyphomonas neptunium ATCC 15444	3577	1	0,028		
	Ensifer meliloti SM11	7156	2	0,028		
	Acetobacter tropicalis NBRC 101654	3579	1	0,028		
	Dietzia cinnamea P4	3593	1	0,028		
	Kushneria aurantia DSM 21353	3598	1	0,028		
	Saprospira grandis HR1, DSM 2844	3598	1	0,028		
	Ensifer meliloti MVII-I	7213	2	0,028		
	Arhodomonas aquaeolei DSM 8974	3608	1	0,028		

	Rhizobium mongolense USDA 1844	7223	2	0,028		
	Mesorhizobium alhagi CCNWXJ12-2	7244	2	0,028		
	Alicyclobacillus pohliae DSM 22757	3628	1	0,028		
	Maritimibacter sp. HL-12	3629	1	0,028		
	Epilithonimonas tenax DSM 16811	3631	1	0,028		
	Gramella forsetii KT0803	3642	1	0,027		
	Myroides odoratimimus CCUG 3837	3644	1	0,027		
	Marinomonas posidonica IVIA-Po-181	3651	1	0,027		
	Flavobacterium soli DSM 19725	3655	1	0,027		
	Leadbetterella byssophila 4M15, DSM 17132	3665	1	0,027		
	Mycobacterium tuberculosis RGTB423	3670	1	0,027		
	Bradyrhizobium japonicum in8p8	7353	2	0,027		
	Mesorhizobium loti MAFF303099	7356	2	0,027		
	Myxococcus fulvus HW-1	7362	2	0,027		
	Bradyrhizobium japonicum is5	7363	2	0,027		
	Dactylococcopsis salina PCC 8305	3685	1	0,027		
	Elizabethkingia anophelis Ag1	3686	1	0,027		
	Salinimonas chungwhensis DSM 16280	3691	1	0,027		

	Mesorhizobium loti NZP2037	7388	2	0,027		
	Algoriphagus mannitolivorans DSM 15301	3700	1	0,027		
	Azovibrio restrictus DSM 23866	3703	1	0,027		
	Flavobacterium daejeonense DSM 17708	3711	1	0,027		
	Salsuginibacillus kocurii DSM 18087	3711	1	0,027		
	Chitinilyticum litopenaei DSM 21440	3715	1	0,027		
	Myroides odoratimimus CIP 101113	3723	1	0,027		
	Dasania marina DSM 21967	3729	1	0,027		
	Neptuniibacter caesariensis MED92	3735	1	0,027		
	Halomonas zhanjiangensis DSM 21076	3738	1	0,027		
	Cupriavidus sp. UYPR2.512	7487	2	0,027		
	Photobacterium damselae damselae CIP 102761	3751	1	0,027		
	Thiomonas arsenitoxydans 3As	3753	1	0,027		
	Spongiibacter tropicus DSM 19543	3758	1	0,027		
	Methylocapsa acidiphila B2	3762	1	0,027		
	Psychromonas hadalis ATCC BAA-638	3773	1	0,027		
	Sphingomonas melonis C3	3774	1	0,026		
	Acinetobacter baumannii AB900	3783	1	0,026		

	Cupriavidus basilensis OR16	7600	2	0,026		
	Methyloversatilis sp. NVD	3821	1	0,026		
	Isosphaera pallida IS1B, ATCC 43644	3823	1	0,026		
	Bordetella pertussis Tohama I	3833	1	0,026		
	Brevibacterium linens BL2	3833	1	0,026		
	Arsenicicoccus bolidensis DSM 15745	3836	1	0,026		
	Nesiotobacter exalbescens DSM 16456	3836	1	0,026		
	Simiduia agarivorans SA1	3836	1	0,026		
	Thermocrispum agreste DSM 44070	3841	1	0,026		
	Rhizobium sp. OR 191	7704	2	0,026		
	Simiduia agarivorans DSM 21679	3856	1	0,026		
	Caldithrix abyssi LF13, DSM 13497	3860	1	0,026		
	Myroides odoratimimus CIP 103059	3862	1	0,026		
	Psychromonas ingrahamii 37	3877	1	0,026		
	Halomonas sp. TD01	3889	1	0,026		
	Microbacterium sp. URHA0036	3890	1	0,026		
	Myroides odoratus DSM 2801	3892	1	0,026		
	Ruania albidiflava DSM 18029	3892	1	0,026		
	Bradyrhizobium sp. BTAi1	7819	2	0,026		

	Sphingomonas melonis FR1	3920	1	0,026		
	Asticcacaulis excentricus CB 48	3921	1	0,026		
	Pedobacter saltans Steyn 113, DSM 12145	3921	1	0,026		
	Burkholderia rhizoxinica HKI 454	3938	1	0,025		
	Marinobacter sp. BSs20148	3944	1	0,025		
	Algoriphagus marincola DSM 16067	3951	1	0,025		
	Methylocystis rosea SV97T	3954	1	0,025		
	Sphingomonas melonis DAPP-PG 224	3959	1	0,025		
	Gordonia sihwensis NBRC 108236	3960	1	0,025		
	Bradyrhizobium japonicum USDA 135	7923	2	0,025		
	Saccharomonospora viridis P101, DSM 43017	3962	1	0,025		
	Acinetobacter sp. DR1	3963	1	0,025		
	Ruegeria sp. TM1040	3964	1	0,025		
	Acidovorax sp. JHL-9	3974	1	0,025		
	Advenella kashmirensis WT001	3980	1	0,025		
	Burkholderia sp. JPY251	7978	2	0,025		
	Ferrimonas futtsuensis DSM 18154	3989	1	0,025		
	Bradyrhizobium japonicum WSM1743	7983	2	0,025		
	Joostella marina En5, DSM 19592	4004	1	0,025		

	Pseudogulbenkiania ferrooxidans 2002	4011	1	0,025		
	Methyloversatilis sp. FAM1	4012	1	0,025		
	Rhodocyclaceae bacterium RZ94	4012	1	0,025		
	Sinobacter flavus DSM 18980	4012	1	0,025		
	Pseudanabaena sp. PCC 7367	4015	1	0,025		
	Azohydromonas australica DSM 1124	8046	2	0,025		
	Gordonia neofelifaecis NRRL B-59395	4034	1	0,025		
	Saccharomonospora halophila 8	4035	1	0,025		
	Alteromonas macleodii Balearic Sea AD45	4054	1	0,025		
	Flavobacterium rivuli DSM 21788	4056	1	0,025		
	Bradyrhizobium canariense WSM4349	8114	2	0,025		
	Halobacillus kuroshimensis DSM 18393	4062	1	0,025		
	Sphingomonas sp. S17	4066	1	0,025		
	Aliivibrio salmonicida LFI1238	4075	1	0,025		
	Amycolatopsis benzoatilytica DSM 43387	8155	2	0,025		
	Gordonia kroppenstedtii DSM 45133	4078	1	0,025		
	Thiomonas sp. FB-6, DSM 25805	4082	1	0,024		
	Shewanella putrefaciens	4083	1	0,024		

	CN-32					
	Vibrio caribbenthicus ATCC BAA-2122	4084	1	0,024		
	Algoriphagus vanfongensis DSM 17529	4085	1	0,024		
	Photobacterium leiognathi mandapamensis svers.1.1	4087	1	0,024		
	Flexibacter litoralis Fx l1, DSM 6794	4090	1	0,024		
	Pseudogulbenkiania sp. MAI-1	4097	1	0,024		
	Algicola sagamiensis DSM 14643	4098	1	0,024		
	Actinoplanes missouriensis NBRC 102363	8202	2	0,024		
	Wenxinia marina DSM 24838	4104	1	0,024		
	Luteibacter sp. 22Crub2.1	4109	1	0,024		
	Paludibacterium yongneupense DSM 18731	4116	1	0,024		
	Bradyrhizobium japonicum USDA 4	8243	2	0,024		
	Pseudogulbenkiania sp. NH8B	4124	1	0,024		
	Fluviicola taffensis RW262, DSM 16823	4131	1	0,024		
	Pantoea sp. Sc1	4133	1	0,024		
	Leptospira kirschneri sv. Valbuzzi 200702274	4138	1	0,024		
	Cellulomonas sp. URHD0024	4141	1	0,024		
	Caulobacter crescentus OR37	4145	1	0,024		

	Flavobacterium subsaxonicum DSM 21790	4147	1	0,024		
	Chryseobacterium gregarium DSM 19109	4152	1	0,024		
	Gloeocapsa sp. PCC 73106	4152	1	0,024		
	Pantoea ananatis AJ13355	4167	1	0,024		
	Shewanella sp. HN-41	4179	1	0,024		
	Marinobacter algicola DG893	4180	1	0,024		
	Patulibacter americanus DSM 16676	4180	1	0,024		
	Bradyrhizobium sp. YR681	8377	2	0,024		
	Caldilinea aerophila STL-6-01, DSM 14535	4192	1	0,024		
	Bradyrhizobium japonicum USDA 110	8402	2	0,024		
	Rhodobacterales sp. Y4I	4206	1	0,024		
	Paracoccus sp. J55	4219	1	0,024		
	Psychromonas arctica DSM 14288	4219	1	0,024		
	Aliagarivorans taiwanensis DSM 22990	4224	1	0,024		
	Halobacillus halophilus DSM 2266	4224	1	0,024		
	Leptospira borgpetersenii sv. Castellonis 200801910	4225	1	0,024		
	Hyphomicrobium sp. 99	4226	1	0,024		
	Pseudomonas cremoricolorata DSM 17059	4235	1	0,024		
	Shewanella sp. W3-18-1	4237	1	0,024		
	Leptospira kirschneri	4238	1	0,024		

	200801774					
	Gordonia paraffinivorans NBRC 108238	4243	1	0,024		
	Moritella marina ATCC 15381	4245	1	0,024		
	Amycolatopsis taiwanensis DSM 45107	8504	2	0,024		
	Marinobacter manganoxydans MnI7-9	4254	1	0,024		
	Thermocrispum municipale DSM 44069	4256	1	0,023		
	Sulfitobacter sp. GAI101	4258	1	0,023		
	Bacillus clausii KSM-K16	4261	1	0,023		
	Oceanicola batsensis HTCC2597	4261	1	0,023		
	Nocardia brasiliensis ATCC 700358	8548	2	0,023		
	Bacillus atrophaeus 1942	4282	1	0,023		
	Bradyrhizobiaceae bacterium SG-6C	4287	1	0,023		
	Leptospira interrogans sv. Muenchen Brem 129	4287	1	0,023		
	Actinopolyspora iraqiensis IQ-H1	4294	1	0,023		
	Teredinibacter turnerae T0609	4301	1	0,023		
	Mycobacterium ulcerans Agy99	4306	1	0,023		
	Bradyrhizobium japonicum USDA 124	8621	2	0,023		
	Nitrobacter sp. Nb-311A	4312	1	0,023		
	Saprospira grandis Lewin	4324	1	0,023		

	Gordonia malaquae NBRC 108250	4326	1	0,023		
	Methylovulum miyakonense HT12	4327	1	0,023		
	Mycobacterium xenopi RIVM700367	4332	1	0,023		
	Pantoea ananatis LMG 20103	4340	1	0,023		
	Arthrobacter sp. 31Cvi3.1E	4342	1	0,023		
	Solirubrobacter soli DSM 22325	8699	2	0,023		
	Gracilibacillus lacisalsi DSM 19029	4363	1	0,023		
	Shewanella benthica KT99	4366	1	0,023		
	Xenorhabdus bovienii SS-2004	4373	1	0,023		
	Leptospira interrogans UI 09600	4382	1	0,023		
	Saccharomonospora glauca K62, DSM 43769	4386	1	0,023		
	Methylobacterium sp. 77	4387	1	0,023		
	Phaeobacter daeponensis TF-218, DSM 23529 (scaffold version)	4388	1	0,023		
	Deinococcus apachensis DSM 19763	4394	1	0,023		
	Alteromonas macleodii AltDE1	4405	1	0,023		
	Mycobacterium avium paratuberculosis K-10	4415	1	0,023		
	Vibrio harveyi HY01	4421	1	0,023		
	Shewanella baltica OS183	4424	1	0,023		

	Hymenobacter roseosalivarius AA-718, DSM 11622	4432	1	0,023		
	Ochrobactrum intermedium LMG 3301	4432	1	0,023		
	Bacillus pseudofirmus OF4	4434	1	0,023		
	Leptospira interrogans sv. Grippotyphosa UI 08434	4439	1	0,023		
	Microbulbifer variabilis ATCC 700307	4439	1	0,023		
	Anabaena circinalis AWQC310F (Submitted file with automatic translation by Kostas)	4443	1	0,023		
	Deinococcus peraridilitoris KR-200, DSM 19664	4466	1	0,022		
	Gordonia effusa NBRC 100432	4471	1	0,022		
	Kineosphaera limosa NBRC 100340	4471	1	0,022		
	Rhodonellum psychrophilum DSM 17998	4476	1	0,022		
	Bacillus sp. 123MFChir2	4485	1	0,022		
	Methylosinus sp. LW4	4496	1	0,022		
	Escherichia fergusonii UMN026, ATCC 35469	4498	1	0,022		
	Gordonia namibiensis NBRC 108229	4498	1	0,022		
	Xanthomonas axonopodis pv. citri 306	4501	1	0,022		
	Leptospira interrogans sv. Pomona Kennewicki LC82-25	4502	1	0,022		
	Xanthomonas campestris pv. musacearum NCPPB	4510	1	0,022		

	4381					
	Leptospira kirschneri H1	4511	1	0,022		
	Aliagarivorans marinus DSM 23064	4519	1	0,022		
	Ferrimonas kyonanensis DSM 18153	4524	1	0,022		
	Methylomicrobium buryatense 5G	4530	1	0,022		
	Paenibacillus sp. D14	4532	1	0,022		
	Pantoea ananatis PA13	4542	1	0,022		
	Starkeya novella DSM 506	4563	1	0,022		
	Vibrio ichthyoenteri ATCC 700023	4567	1	0,022		
	Methylosinus trichosporium OB3b	4568	1	0,022		
	Rhodococcus equi 103S	4570	1	0,022		
	Vibrio splendidus LGP32	4572	1	0,022		
	Gordonia amicalis NBRC 100051	4578	1	0,022		
	Psychromonas ossibalaenae ATCC BAA-1528	4580	1	0,022		
	Kordia algicida OT-1	4584	1	0,022		
	Saccharomonospora sp. CNQ490	4590	1	0,022		
	Thiothrix nivea JP2, DSM 5205	4594	1	0,022		
	Leptospira interrogans sv. Pyrogenes R168	4605	1	0,022		
	Roseobacter sp. MED193	4605	1	0,022		
	Leisingera methylohalidivorans MB2,	4608	1	0,022		

	DSM 14336					
	Shewanella baltica BA175	4609	1	0,022		
	Methylobacterium marinus A45	4610	1	0,022		
	Roseobacter sp. SK209-2-6	4610	1	0,022		
	Shewanella baltica OS185	4618	1	0,022		
	Shewanella baltica OS625	4639	1	0,022		
	Leptospira interrogans sv. Grippotyphosa UI 08368	4640	1	0,022		
	Loktanella sp. SE62	4641	1	0,022		
	Gordonia sputi NBRC 100414	4642	1	0,022		
	Amycolatopsis mediterranei S699	9291	2	0,022		
	Amycolatopsis mediterranei U32	9292	2	0,022		
	Glycomyces arizonensis DSM 44726	4653	1	0,021		
	Arthrobacter nitroguajacolicus Rue61a	4655	1	0,021		
	Shewanella oneidensis MR-1	4657	1	0,021		
	Mycobacterium thermoresistibile ATCC 19527	4662	1	0,021		
	Gordonia aichiensis NBRC 108223	4663	1	0,021		
	Methylomonas methanica MC09	4664	1	0,021		
	Salinispora tropica CNB-440	4664	1	0,021		
	Fischerella sp. JSC-11	4671	1	0,021		

	Leptospira interrogans 2002000624	4675	1	0,021		
	Methylobacter luteus IMV-B-3098	4675	1	0,021		
	Pseudomonas mendocina DLHK	4677	1	0,021		
	Flavobacterium sp. URHB0058	4682	1	0,021		
	Mycobacterium avium avium ATCC 25291	4684	1	0,021		
	Leptospira interrogans sv. Bataviae L1111	4687	1	0,021		
	Gordonia alkanivorans NBRC 16433	4709	1	0,021		
	Xanthobacteraceae bacterium 501b	4709	1	0,021		
	Zunongwangia profunda SM-A87	4709	1	0,021		
	Acidovorax sp. NO-1	4729	1	0,021		
	Pseudomonas mendocina ymp	4730	1	0,021		
	Vibrio sp. MED222	4733	1	0,021		
	Methylocystis sp. Rockwell, ATCC 49242	4734	1	0,021		
	Nocardioides halotolerans DSM 19273	4734	1	0,021		
	Shewanella baltica OS155	4741	1	0,021		
	Serratia odorifera 4Rx13	4743	1	0,021		
	Leptospira interrogans sv. Pyrogenes 2006006960	4744	1	0,021		
	Asticcacaulis biprosthecum C19, ATCC 27554	4756	1	0,021		

	Granulicella tundricola MP5ACTX9	4757	1	0,021		
	Amycolicicoccus subflavus DQS3-9A1	4759	1	0,021		
	Maritimibacter alkaliphilus HTCC2654	4763	1	0,021		
	Pantoea ananatis LMG 5342	4763	1	0,021		
	Synechococcus sp. PCC 7336	4768	1	0,021		
	Streptomyces violaceusniger Tu 4113	9557	2	0,021		
	Azorhizobium caulinodans ORS 571	4781	1	0,021		
	Salinispora pacifica CNT796	4782	1	0,021		
	Burkholderia mallei 2002721280	4792	1	0,021		
	Herbaspirillum seropedicae SmR1	4799	1	0,021		
	Acidovorax sp. KKS102	4803	1	0,021		
	Mycobacterium massiliense 2B-0307	4810	1	0,021		
	Amycolatopsis mediterranei S699, ATCC 13685	9639	2	0,021		
	Pseudanabaena sp. PCC 7429	4820	1	0,021		
	Prochlorothrix hollandica PCC 9006	4823	1	0,021		
	Roseovarius sp. 217	4823	1	0,021		
	Candidatus Korebacter versatilis Ellin345	4837	1	0,021		
	Burkholderia mallei JHU	4838	1	0,021		
	Salinispora pacifica CNT148	4843	1	0,021		

	Gordonia rubripertincta NBRC 101908	4857	1	0,021		
	Shewanella baltica OS195	4857	1	0,021		
	Mycobacterium massiliense 2B-0107	4862	1	0,021		
	Mycobacterium massiliense 2B-0626	4862	1	0,021		
	Salinispora tropica CNS416	4862	1	0,021		
	Salinispora arenicola CNR416	4863	1	0,021		
	Acidovorax avenae citrulli AAC00-1	4868	1	0,021		
	Mycobacterium massiliense 2B-0912-R	4868	1	0,021		
	Mycobacterium massiliense 2B-0912-S	4871	1	0,021		
	Mycobacterium abscessus 47J26	4872	1	0,021		
	Nitratireductor indicus C115	4872	1	0,021		
	Rhodococcus pyridinivorans AK37	4875	1	0,021		
	Salinispora tropica CNS197	4882	1	0,020		
	Comamonas testosteroni CNB-1	4887	1	0,020		
	Burkholderia mallei GB8 horse 4	4888	1	0,020		
	Salinispora pacifica DSM 45548	4888	1	0,020		
	Phaeobacter arcticus DSM 23566	4889	1	0,020		
	Salinispora arenicola CNH646	4904	1	0,020		

	Leptospira interrogans UI 12758	4905	1	0,020		
	Agrobacterium albertimagni AOL15	4911	1	0,020		
	Salinispora tropica CNB476	4911	1	0,020		
	Burkholderia mallei FMH	4912	1	0,020		
	Rhodopseudomonas palustris CGA009	4918	1	0,020		
	Leptospira interrogans 2002000623	4922	1	0,020		
	Vibrio sp. EJY3	4935	1	0,020		
	Moritella sp. PE36	4937	1	0,020		
	Acidovorax delafieldii 2AN	4945	1	0,020		
	Leptospira interrogans UI 12621	4945	1	0,020		
	Mycobacterium hassiacum DSM 44199	4948	1	0,020		
	Burkholderia mallei 10399	4951	1	0,020		
	Salinarimonas rosea DSM 21201	4952	1	0,020		
	Paenibacillus curdlanolyticus YK9	4957	1	0,020		
	Amycolatopsis balhimycina DSM 44591	9919	2	0,020		
	Ancylobacter sp. FA202	4964	1	0,020		
	Phyllobacterium sp. YR531	4970	1	0,020		
	Sphingobacterium spiritivorum ATCC 33300	4972	1	0,020		
	Salinispora pacifica CNT851	4974	1	0,020		
	Mycobacterium massiliense 1S-151-0930	4983	1	0,020		

	Mycobacterium massiliense 1S-152-0914	4985	1	0,020		
	Shewanella baltica OS117	4985	1	0,020		
	Mycobacterium massiliense 1S-154-0310	4986	1	0,020		
	Actinoalloteichus cyanogriseus DSM 43889	4992	1	0,020		
	Pantoea sp. YR343	4992	1	0,020		
	Salinispora pacifica CNT603	4992	1	0,020		
	Mycobacterium massiliense 1S-153-0915	4993	1	0,020		
	Gordonia bronchialis 3410, DSM 43247	5002	1	0,020		
	Frankia sp. Iso899	5006	1	0,020		
	Erwinia billingiae Eb661	5015	1	0,020		
	Algoriphagus terrigena DSM 22685	5016	1	0,020		
	Pseudomonas syringae pv. syringae B64	5021	1	0,020		
	Salinispora arenicola CNY234	5027	1	0,020		
	Pseudomonas putida BIRD-1	5046	1	0,020		
	Shewanella piezotolerans WP3	5047	1	0,020		
	Rahnella aquatilis HX2	5060	1	0,020		
	Salinispora pacifica DSM 45544	5064	1	0,020		
	Oscillatoriales sp. JSC-12	5081	1	0,020		
	Salinispora pacifica CNT001	5088	1	0,020		
	Pseudomonas taiwanensis	5091	1	0,020		

	DSM 21245					
	Xanthomonas gardneri PDDCC 1620, ATCC 19865	5091	1	0,020		
	Salinispora pacifica CNT609	5099	1	0,020		
	Salinispora pacifica CNQ768	5100	1	0,020		
	Patulibacter minatonensis KV-614, DSM 18081	5104	1	0,020		
	Salinispora arenicola CNS243	5113	1	0,020		
	Saccharomonospora paurometabolica YIM 90007	5115	1	0,020		
	Rhodococcus equi ATCC 33707	5116	1	0,020		
	Vibrio harveyi 1DA3	5117	1	0,020		
	Rahnella aquatilis CIP 78.65, ATCC 33071	5118	1	0,020		
	Serratia plymuthica AS9	5138	1	0,019		
	Serratia plymuthica AS12	5139	1	0,019		
	Serratia plymuthica AS13	5139	1	0,019		
	Labrenzia alexandrii DFL-11	5144	1	0,019		
	Vibrio coralliilyticus ATCC BAA-450	5144	1	0,019		
	Geodermatophilus obscurus G-20, DSM 43160	5155	1	0,019		
	Paracoccus denitrificans PD1222	5158	1	0,019		
	Salinispora pacifica CNR894	5163	1	0,019		
	Salinispora tropica CNT261	5174	1	0,019		
	Salinispora pacifica CNY330	5176	1	0,019		
	Mycobacterium sp.	5177	1	0,019		

	MOTT36Y					
	Pantoea strewartii stewartii DC283	5181	1	0,019		
	Salinispora arenicola CNH905	5185	1	0,019		
	Salinispora arenicola CNT005	5186	1	0,019		
	Mycobacterium intracellulare ATCC 13950	5193	1	0,019		
	Xanthobacter sp. 126	5195	1	0,019		
	Mycobacterium intracellulare MOTT-02	5198	1	0,019		
	Smaragdicoccus niigatensis DSM 44881	5200	1	0,019		
	Comamonas testosteroni S44	5218	1	0,019		
	Leisingera aquimarina DSM 24565 (scaffold version)	5218	1	0,019		
	Rhodococcus sp. 114MFTsu3.1	5236	1	0,019		
	Salinispora arenicola CNY282	5236	1	0,019		
	Streptomyces sp. CNS606	5240	1	0,019		
	Salinispora arenicola CNT857	5242	1	0,019		
	Salinispora pacifica CNS103	5243	1	0,019		
	Pseudomonas syringae pv. syringae B728a	5245	1	0,019		
	Salinispora pacifica CNY237	5257	1	0,019		
	Geminicoccus roseus DSM 18922	5259	1	0,019		
	Salinispora tropica CNR699	5261	1	0,019		

	Salinispora arenicola CNS744	5262	1	0,019		
	Salinispora arenicola CNY231	5262	1	0,019		
	Martelella mediterranea DSM 17316	5266	1	0,019		
	Salinispora arenicola CNX891	5275	1	0,019		
	Mycobacterium intracellulare ATCC 13950	5282	1	0,019		
	Rahnella sp. Y9602	5285	1	0,019		
	Nitriliruptor alkaliphilus DSM 45188	5296	1	0,019		
	Mycobacterium intracellulare MOTT-64	5297	1	0,019		
	Serratia plymuthica RVH1	5297	1	0,019		
	Mycobacterium avium 104	5305	1	0,019		
	Pseudomonas putida S16	5307	1	0,019		
	Burkholderia mallei SAVP1	5309	1	0,019		
	Salinispora arenicola CNS051	5312	1	0,019		
	Mycobacterium indicus pranii MTCC 9506	5318	1	0,019		
	Salinispora arenicola CNT859	5320	1	0,019		
	Salinispora arenicola CNS-991	5327	1	0,019		
	Rhodococcus sp. 29MFTsu3.1	5332	1	0,019		
	Phaeobacter caeruleus 13, DSM 24564	5335	1	0,019		
	Sphingobacterium sp. 21	5340	1	0,019		

	Leptolyngbya sp. PCC 6406	5341	1	0,019		
	Vibrio splendidus 12B01	5346	1	0,019		
	Rhodopseudomonas palustris TIE-1	5377	1	0,019		
	Nocardia sp. 348MFTsu5.1	5380	1	0,019		
	Klebsiella pneumoniae NTUH-K2044	5385	1	0,019		
	Arthrospira platensis Paraca	5401	1	0,019		
	Pseudomonas syringae CC1544 (CC1544)	5409	1	0,018		
	Azorhizobium doebereinerae UFLA1-100	5415	1	0,018		
	Pseudomonas putida F1	5423	1	0,018		
	Pseudomonas syringae pv. phaseolicola 1448A	5436	1	0,018		
	Vibrio shilonii AK1	5438	1	0,018		
	Serratia plymuthica A30	5448	1	0,018		
	Agrobacterium vitis S4	5455	1	0,018		
	Pseudomonas sp. TA043 (TA043)	5460	1	0,018		
	Bacillus sp. URHB0009	5468	1	0,018		
	Bacillus cereus BDRD-Cer4	5472	1	0,018		
	Rhizobium sp. PDO1-076	5480	1	0,018		
	Mycobacterium phlei RIVM601174	5489	1	0,018		
	Acidobacteriaceae bacterium KBS 83	5491	1	0,018		
	Burkholderia mallei ATCC 23344	5500	1	0,018		
	Vibrionales sp. SWAT-3	5502	1	0,018		

	Conexibacter woesei Iso977N	5509	1	0,018		
	Bacillus cereus ATCC 14579	5513	1	0,018		
	Janthinobacterium sp. CG3	5535	1	0,018		
	Nocardia cyriacigeorgica GUH-2	5560	1	0,018		
	Pseudomonas syringae TLP2 (TLP2)	5562	1	0,018		
	Promicromonospora sukumoe 327MFSha3.1	5569	1	0,018		
	Pseudomonas syringae pv. morsprunorum M302280PT (Morsprunorum)	5579	1	0,018		
	Salinispora arenicola CNY280	5597	1	0,018		
	Aquimarina latercula DSM 2041	5610	1	0,018		
	Pseudomonas syringae pv. aesculi 0893_23 (Aesculi)	5614	1	0,018		
	Synechococcus sp. PCC 7335	5626	1	0,018		
	Enterobacter cloacae cloacae ATCC 13047	5627	1	0,018		
	Pseudomonas cannabina pv. alisalensis T3C (draft assemply)	5630	1	0,018		
	Burkholderia mallei NCTC 10229	5635	1	0,018		
	Bacillus thuringiensis sv andalousiensis BGSC 4AW1	5638	1	0,018		
	Pseudomonas syringae CC1513 (CC1513)	5648	1	0,018		
	Microcoleus vaginatus FGP-2	5651	1	0,018		

	Pseudomonas syringae pv aesculi 2250	5666	1	0,018		
	Pseudomonas aeruginosa PAO1	5671	1	0,018		
	Pseudomonas syringae Pph1448A (1448A gold standard)	5672	1	0,018		
	Nocardioidaceae bacterium Broad-1	5686	1	0,018		
	Nostoc sp. PCC 7524	5688	1	0,018		
	Pseudomonas syringae pv aesculi NCPPB 3681	5689	1	0,018		
	Pseudomonas aeruginosa C3719	5696	1	0,018		
	Pseudomonas sp. GM30	5696	1	0,018		
	Mycobacterium sp. MCS	5704	1	0,018		
	Arthrospira sp. PCC 8005	5718	1	0,017		
	Pseudomonas cannabina pv. alisalensis BS91 (draft assemply)	5721	1	0,017		
	Pseudomonas syringae pv. tomato DC3000	5721	1	0,017		
	Arthrospira maxima CS-328	5730	1	0,017		
	Pseudomonas syringae CC440 (CC440)	5767	1	0,017		
	Pseudomonas aeruginosa M18	5769	1	0,017		
	Anabaena variabilis ATCC 29413	5772	1	0,017		
	Saccharomonospora marina XMU15, DSM 45390	5784	1	0,017		
	Pseudomonas syringae pv. maculicola ES4326	5810	1	0,017		

	(Maculicola)					
	Pseudomonas sp. GM25	5836	1	0,017		
	Calothrix sp. PCC 6303	5840	1	0,017		
	Pseudomonas sp. UB246 (UB246)	5861	1	0,017		
	Massilia niastensis DSM 21313	5887	1	0,017		
	Pseudomonas syringae pv. tomato K40	5900	1	0,017		
	Cyanothece sp. PCC 7424	5933	1	0,017		
	Pseudomonas umsongensis 20MFCvi1.1	5959	1	0,017		
	Pseudomonas aeruginosa DK2	5960	1	0,017		
	Pseudomonas sp. 35MFCvi1.1	5965	1	0,017		
	Burkholderia mallei NCTC 10247	5978	1	0,017		
	Pseudomonas sp. GM60	5991	1	0,017		
	Bacillus cereus G9842	5994	1	0,017		
	Pseudomonas aeruginosa UCBPP-PA14	5994	1	0,017		
	Crenothrix polyspora	36101	6	0,017		
	Pseudomonas aeruginosa PAb1	6019	1	0,017		
	Pseudomonas syringae CC94 (CC94)	6027	1	0,017		
	Pseudomonas sp. GM55	6068	1	0,016		
	Verrucosispora maris AB-18-032	6069	1	0,016		
	Streptomyces sp. CNY228	6082	1	0,016		

	Mycobacterium sp. KMS	6089	1	0,016		
	Janthinobacterium sp. HH01	6100	1	0,016		
	Streptomyces albus J1074	6114	1	0,016		
	Pseudomonas sp. 2_1_26	6116	1	0,016		
	Pseudomonas sp. M1	6131	1	0,016		
	Arthrospira platensis C1 (Draft2 circular genome)	6153	1	0,016		
	Burkholderia sp. CCGE1001	6157	1	0,016		
	Rhizobium sp. CF122	6157	1	0,016		
	Pseudomonas syringae CC1543 (CC1543)	6167	1	0,016		
	Fischerella sp. PCC 9431	6190	1	0,016		
	Lyngbya sp. CCY 8106	6191	1	0,016		
	Nostoc sp. PCC 7120	6222	1	0,016		
	Mesorhizobium sp. WSM3626	6243	1	0,016		
	Pseudomonas syringae pv. tabaci ATCC 11528 (Tabaci)	6261	1	0,016		
	Pseudomonas syringae UB303 (UB303)	6298	1	0,016		
	Pseudomonas sp. GM80	6334	1	0,016		
	Pseudomonas sp. GM49	6340	1	0,016		
	Frankia sp. BMG5.12	6342	1	0,016		
	Azospirillum lipoferum 4B	6349	1	0,016		
	Microcystis aeruginosa NIES-843	6360	1	0,016		
	Pseudomonas aeruginosa PA7	6396	1	0,016		
	Burkholderia mallei PRL-20	6399	1	0,016		

	Pseudomonas syringae pv. oryzae 1_6 (Curated version without duplicated contigs)	6407	1	0,016		
	Azospirillum sp. B510	6417	1	0,016		
	Variovorax paradoxus S110	6450	1	0,016		
	Ensifer medicae Di28	6469	1	0,015		
	Ensifer medicae WSM244	6495	1	0,015		
	Rhodococcus erythropolis PR4	6505	1	0,015		
	Methylobacterium radiotolerans JCM 2831	6510	1	0,015		
	Mycobacterium parascrofulaceum ATCC BAA-614	6510	1	0,015		
	Pseudomonas syringae pv. tabaci ATCC 11528	6527	1	0,015		
	Ensifer arboris LMG 14919	6545	1	0,015		
	Verrucomicrobium spinosum DSM 4136	6584	1	0,015		
	Ensifer medicae WSM419	6599	1	0,015		
	Arthrospira platensis NIES-39	6676	1	0,015		
	Beggiatoa sp. PS	6698	1	0,015		
	Deinococcus hopiensis KR-140, DSM 18049	6701	1	0,015		
	Ensifer medicae WSM1369	6735	1	0,015		
	Promicromonospora kroppenstedtii RS16, DSM 19349	6746	1	0,015		
	Rhodococcus erythropolis SK121	6767	1	0,015		
	Pseudomonas sp. GM78	6804	1	0,015		

	Proteobacteria bacterium JGI 0000113-P07	6852	1	0,015		
	Streptomyces purpureus KA281, ATCC 21405	6892	1	0,015		
	Mesorhizobium sp. WSM2561	6910	1	0,014		
	Leptolyngbya boryana PCC 6306	6911	1	0,014		
	Rhizobium sp. STM6155	6926	1	0,014		
	Pseudomonas cannabina pv. alisalensis PSa1-3 (Draft assemply with Ion Torrent)	6930	1	0,014		
	Bradyrhizobium sp. S23321	6943	1	0,014		
	Oscillatoria nigro-viridis PCC 7112	7006	1	0,014		
	Ensifer medicae WSM4191	7099	1	0,014		
	Oscillatoria sp. PCC 10802	7104	1	0,014		
	Fischerella sp. PCC 9605	7130	1	0,014		
	Pseudomonas syringae pv. mori MAFF301020 (Mori)	7228	1	0,014		
	Amycolatopsis methanolica 239, DSM 44096	7236	1	0,014		
	Pseudomonas syringae pv. aptata DSM 50252 (Aptata)	7238	1	0,014		
	Saccharopolyspora erythraea NRRL 2338	7290	1	0,014		
	Mycobacterium smegmatis JS623	7311	1	0,014		
	Saccharopolyspora erythraea NRRL 2338	7356	1	0,014		
	Burkholderia sp. CCGE1002	7358	1	0,014		
	Bradyrhizobium sp.	7430	1	0,013		

	WSM471					
	Niastella koreensis GR20-10, DSM 17620	7444	1	0,013		
	Rhizobium sullae WSM1592	7526	1	0,013		
	Singulisphaera acidiphila MOB10, DSM 18658	7683	1	0,013		
	Streptomyces sp. C	7768	1	0,013		
	Bradyrhizobium sp. WSM1417	7772	1	0,013		
	Azospirillum brasilense Sp245	7962	1	0,013		
	Amycolatopsis sp. 75iv2, ATCC 39116	8314	1	0,012		
	Actinoplanes sp. SE50/110	8385	1	0,012		
	Hamadaea tsunoensis DSM 44101	8415	1	0,012		
	Pseudomonas cannabina pv. alisalensis PSa866 (Draft assemply with Ion Torrent)	8436	1	0,012		
	Bradyrhizobium sp. WSM1253	8498	1	0,012		
	Bradyrhizobium japonicum USDA 122	8530	1	0,012		
	Nonomuraea coxensis DSM 45129	8548	1	0,012		
	Amycolatopsis thermoflava N1165, DSM 44574	8633	1	0,012		
	Streptomyces sp. AA4	8642	1	0,012		
	Amycolatopsis nigrescens CSC17Ta-90, DSM 44992	8660	1	0,012		
	Bradyrhizobium sp. USDA 3384	9298	1	0,011		

	Bradyrhizobium genosp. SA-4 CB756	9456	1	0,011		
	Pseudomonas syringae pv. japonica M301072PT (Japonica)	9628	1	0,010		
	Microvirga sp. Lut6	10951	1	0,009		
Eukarya	Aureococcus anophagefferens CCMP 1984	11501	43	0,374	0,0429	0,374
	Ostreococcus tauri OTH95	7725	13	0,168		
	Micromonas pusilla CCMP 490(RCC 114)	8366	14	0,167		
	Aspergillus nidulans FGSC A4	9727	16	0,164		
	Emiliania huxleyi CCMP 1516	33333	53	0,159		
	Ostreococcus lucimarinus CCE9901	7640	12	0,157		
	Micromonas pusilla NOUM17, RCC 299	10108	14	0,139		
	Leishmania major Friedlin	1579	2	0,127		
	Phaeodactylum tricornutum CCAP1055/1	10408	13	0,125		
	Aspergillus niger CBS 513.88	14431	18	0,125		
	Cryptococcus neoformans var neoformans B-3501A	6609	8	0,121		
	Penicillium marneffei ATCC 18224	10804	13	0,120		
	Talaromyces stipitatus ATCC 10500	13356	16	0,120		
	Branchiostoma floridae S238N-H82	50817	57	0,112		

	Thalassiosira pseudonana CCMP 1335	11673	13	0,111		
	Aspergillus flavus NRRL 3357	13487	15	0,111		
	Cryptococcus neoformans var neoformans JEC 21	6609	7	0,106		
	Neosartorya fischeri NRRL 181	10697	11	0,103		
	Nectria haematococca MPVI 77-13-4	15708	16	0,102		
	Aspergillus fumigatus Af293	10152	10	0,099		
	Monosiga brevicollis MX1	9171	9	0,098		
	Penicillium chrysogenum Wisconsin 54-1255	13052	11	0,084		
	Proterospongia sp. ATCC 50818	9828	8	0,081		
	Cochliobolus heterostrophus C4, ATCC 48331	12720	10	0,079		
	Theileria annulata Ankara clone C9	11495	9	0,078		
	Arthroderma gypseum CBS 118893	9015	7	0,078		
	Cochliobolus heterostrophus C5	13336	10	0,075		
	Fusarium oxysporum lycopersici FGSC 4286	17426	13	0,075		
	Uncinocarpus reesii 1704	7856	5	0,064		
	Arthroderma benhamiae CBS 112371	8041	5	0,062		
	Trichophyton verrucosum HKI 0517	8086	5	0,062		
	Gibberella zeae PH-1	11640	7	0,060		

	Dictyostelium discoideum AX4	13362	8	0,060		
	Spizellomyces punctatus DAOM BR117	8779	5	0,057		
	Pyrenophora tritici-repentis Pt-1C-BFP	12298	7	0,057		
	Aspergillus terreus NIH2624	10556	6	0,057		
	Arthroderma otae CBS 113480	8847	5	0,057		
	Phaeosphaeria nodorum SN15	16126	9	0,056		
	Coccidioides posadasii C735 delta SOWgp	7255	4	0,055		
	Lottia gigantea	23851	13	0,055		
	Amphimedon queenslandica	30060	16	0,053		
	Verticillium albo-atrum VaMs.102	10487	5	0,048		
	Magnaporthe grisea 70-15	13146	6	0,046		
	Ciona intestinalis	14002	6	0,043		
	Aspergillus clavatus NRRL 1	9356	4	0,043		
	Saitoella complicata NRRL Y-17804	7034	3	0,043		
	Botryotinia fuckeliana B05.10	16584	7	0,042		
	Debaryomyces hansenii var hansenii CBS767	7108	3	0,042		
	Coemansia reversa NRRL 1564	7347	3	0,041		
	Lachancea thermotolerans CBS 6340	5371	2	0,037		
	Capitella teleta I ESC-2004	32415	12	0,037		

	Naegleria gruberi NEG-M	16619	6	0,036		
	Nadsonia fulvescens elongata AJ 4281, DSM 6958	5657	2	0,035		
	Scheffersomyces stipitis CBS 6054	5816	2	0,034		
	Lodderomyces elongisporus NRRL YB-4239	5905	2	0,034		
	Candida dubliniensis CD36	5972	2	0,033		
	Meyerozyma guilliermondii ATCC 6260	6062	2	0,033		
	Candida (Clavispora) lusitaniae ATCC 42720	6153	2	0,033		
	Paracoccidioides brasiliensis Pb01	9254	3	0,032		
	Nasonia vitripennis SymAX	9373	3	0,032		
	Ajellomyces capsulatus NAm1	9402	3	0,032		
	Phytophthora ramorum Pr102, UCD Pr4	15743	5	0,032		
	Candida tropicalis MYA-3404	6441	2	0,031		
	Ectocarpus siliculosus Ec 32 (CCAP 1310/04)	16284	5	0,031		
	Chlorella sp. NC64A	9791	3	0,031		
	Saprolegnia parasitica CBS 223.65	16484	5	0,030		
	Ustilago maydis 521	6631	2	0,030		
	Thielavia terrestris NRRL 8126	9962	3	0,030		
	Ajellomyces dermatitidis SLH14081	10047	3	0,030		

	Neurospora crassa OR74A	10082	3	0,030		
	Nematostella vectensis CH2 x CH6	27273	8	0,029		
	Bombyx mori Dazao	14623	4	0,027		
	Sclerotinia sclerotiorum 1980 UF-70	14637	4	0,027		
	Acyrthosiphon pisum LSR1	10996	3	0,027		
	Chaetomium globosum CBS 148.51	11232	3	0,027		
	Babesia bovis T2Bo	3781	1	0,026		
	Theileria parva Muguga	4159	1	0,024		
	Leishmania infantum JPCM5 (MCAN/ES/98/LLM-877)	8335	2	0,024		
	Batrachochytrium dendrobatidis JEL423	8806	2	0,023		
	Daphnia pulex Log50	30938	7	0,023		
	Myceliophthora thermophila ATCC 42464	9296	2	0,022		
	Xenopus tropicalis	27916	6	0,021		
	Candida albicans SC5314	14144	3	0,021		
	Chlamydomonas reinhardtii CC-503 cw92 mt+	14546	3	0,021		
	Schizophyllum commune H4-8	14652	3	0,020		
	Phanerochaete chrysosporium RP-78	10048	2	0,020		
	Pichia pastoris GS115	5040	1	0,020		
	Tribolium castaneum Georgia GA2	10196	2	0,020		
	Trypanosoma brucei brucei 927/4 GUTat10.1	10253	2	0,020		

	Torulaspora delbrueckii Wallerstein 129, CBS 1146	5176	1	0,019		
	Caenorhabditis elegans Bristol N2	20935	4	0,019		
	Podospora anserina S mat+	10760	2	0,019		
	Saccharomyces cerevisiae AWRI1631	5451	1	0,018		
	Pediculus humanus corporis USDA	10994	2	0,018		
	Gymnopus luxurians FD-317 M1	22057	4	0,018		
	Saccharomyces cerevisiae YJM789	5903	1	0,017		
	Perkinsus marinus PmCV4CB5 2B3 D4	29653	5	0,017		
	Hypholoma sublateritium	17911	3	0,017		
	Tetrahymena thermophila SB210 (Macronucleus)	24725	4	0,016		
	Saccharomyces cerevisiae S288C	6273	1	0,016		
	Trypanosoma cruzi CL Brener TC3	25105	4	0,016		
	Ricinus communis Hale	31894	5	0,016		
	Anopheles gambiae PEST	13749	2	0,015		
	Yarrowia lipolytica CLIB122	7042	1	0,014		
	Physcomitrella patens patens	35938	5	0,014		
	Volvox carteri f. nagariensis 69-1b	14542	2	0,014		
	Selaginella moellendorfii	22285	3	0,013		
	Tuber melanosporum Mel28	7496	1	0,013		

	Helobdella robusta	23432	3	0,013		
	Serpula lacrymans lacrymans S7.9	16257	2	0,012		
	Thecamonas trahens ATCC 50062	8155	1	0,012		
	Botryobasidium botryosum FD-172 SS1	16526	2	0,012		
	Ixodes scapularis Wikel	24903	3	0,012		
	Drosophila pseudoobscura MV2-25	16737	2	0,012		
	Allomyces macrogynus ATCC 38327	17386	2	0,012		
	Aedes aegypti Liverpool	18091	2	0,011		
	Apis mellifera DH4	9450	1	0,011		
	Zea mays mays cv. B73	106046	11	0,010		
	Trichoplax adhaerens Grell-BS-1999	11520	1	0,009		
	Danio rerio Tuebingen	38017	3	0,008		
	Paramecium tetraurelia d4-2	39642	3	0,008		
	Moniliophthora perniciosa FA553	13674	1	0,007		
	Coprinopsis cinerea okayama7#130	13832	1	0,007		
	Drosophila grimshawi G1, TSC#15287-2541.00	14717	1	0,007		
	Drosophila virilis TSC#15010-1051.87	14974	1	0,007		
	Drosophila mojavensis TSC#15081-1352.22	15187	1	0,007		
	Drosophila erecta TSC#14021-0224.01	15712	1	0,006		

	Drosophila willistoni Gd-H4-1, TSC#14030-0811.24	15877	1	0,006		
	Drosophila ananassae AABBg1, TSC#14024-0371.13	15985	1	0,006		
	Coccidioides immitis RS	16160	1	0,006		
	Drosophila yakuba Tai18E2	16940	1	0,006		
	Oikopleura dioica	17113	1	0,006		
	Drosophila sechellia Rob3c, 14021-0248.25	17321	1	0,006		
	Laccaria bicolor S238N-H82	18215	1	0,005		
	Glycine max cultivar Williams 82	55787	3	0,005		
	Rattus norvegicus BN/SsNHsdMCW	38115	2	0,005		
	Homo sapiens	38612	2	0,005		
	Ailuropoda melanoleuca	20664	1	0,005		
	Drosophila melanogaster y; cn bw sp	21116	1	0,005		
	Culex pipiens quinquefasciatus JHB	22666	1	0,004		
	Vitis vinifera PN40024	24954	1	0,004		
	Sorghum bicolor BTx623	29448	1	0,003		
	Mus musculus C57BL/6J	60745	2	0,003		
	Arabidopsis thaliana Columbia	31392	1	0,003		
	Arabidopsis lyrata lyrata MN47	32549	1	0,003		
	Populus balsamifera trichocarpa	40566	1	0,002		
