# Supplementary material for: The Candidate Phylum Poribacteria by Single-Cell Genomics: New Insights into Phylogeny, Cell-Compartmentation, Eukaryote-Like Repeat Proteins, and Other Genomic Features
Source: PLoS One. 2014 Jan 31;9(1):e87353. doi: 10.1371/journal.pone.0087353 (PMC3909097; doi:10.1371/journal.pone.0087353)
Supplement: Table S11 — Poribacterial phyH gene clusters based on 60% amino acid identity. (PDF) [file pone.0087353.s011.pdf]

Table S11: Poribacterial phyH gene clusters based on 60% amino acid identity.

Cluster number	Number of sequences	Representative sequences (OID)	All sequences (OIDs)	Sequence length (aa)	%ID with representative sequence
0	2	2265137517	2265138005	246	100.0
			2265137517	276	
1	3	2265147978	2265143464	290	100.0
			2265140852	281	79.4
			2265147978	290	
2	5	2265148006	2265146023	286	100.0
			2265141982	143	89.5
			2265147780	287	88.1
			2265138929	286	70.3
			2265148006	286	
3	3	2265141659	2265143874	293	100.0
			2265147874	294	91.8
			2265141659	293	
4	3	2265142603	2265146677	300	100.0
			2265141434	300	89.3
			2265142603	300	
5	4	2265142984	2265148154	263	100.0
			2265137654	264	62.7
			2265141602	263	83.3
			2265142984	263	
6	2	2265143675	2265148021	273	100.0
			2265143675	273	
7	3	2265144124	2265148346	300	100.0
			2265141499	298	86.2
			2265144124	308	
8	4	2265144705	2265142430	257	100.0
			2265143154	259	72.6
			2265142387	147	70.7
			2265144705	257	
9	2	2265148069	2265146986	238	100.0
			2265148069	238	
10	4	2265140654	2265140655	245	100.0
			2265142516	248	64.2
			2265145749	246	66.7
			2265140654	245	
11	1	2265137852	2265137852	274	
12	2	2265137616	2265141298	274	69.7
			2265137616	276	
13	1	2265144816	2265144816	276	
14	2	2265147813	2265140751	274	90.1
			2265147813	274	
15	5	2265138263	2265148267	297	62.0

			2265143189	274	74.5
			2265140118	278	63.7
			2265143406	272	62.0
			2265138263	274	
16	1	2265139533	2265139533	273	
17	2	2265140682	2265145827	269	72.9
			2265140682	269	
18	4	2265147413	2265147484	176	89.2
			2265140552	294	62.7
			2265141549	293	80.5
			2265147413	293	
19	1	2265140145	2265140145	292	
20	3	2265145857	2265138010	247	81.0
			2265141378	247	86.6
			2265145857	247	
21	1	2265138033	2265138033	296	
22	3	2265139165	2265146658	287	72.8
			2265141074	267	73.4
			2265139165	288	
23	3	2265141393	2265138062	276	75.0
			2265145424	278	81.7
			2265141393	278	
24	2	2265145466	2265141633	277	92.4
			2265145466	277	
25	2	2265146478	2265141890	282	80.5
			2265146478	282	
26	2	2265140007	2265144334	322	72.6
			2265140007	240	
27	1	2265139289	2265139289	235	
28	2	2265139795	2265141299	248	69.4
			2265139795	247	
29	1	2265146306	2265146306	241	
30	3	2265142506	2265143566	305	83.9
			2265141486	310	79.0
			2265142506	305	
31	2	2265143770	2265142330	304	84.2
			2265143770	304	
32	1	2265147384	2265147384	165	
33	4	2265141957	2265139671	281	79.3
			2265145195	284	92.0
			2265147492	283	91.3
			2265141957	150	
34	2	2265145666	2265141357	249	92.3
			2265145666	248	
35	2	2265142692	2265138080	261	65.9
			2265142692	263	

36	1	2265139997	2265139997	262	
37	2	2265139858	2265144857	263	69.7
			2265139858	265	
38	1	2265140696	2265140696	264	
39	1	2265147373	2265147373	255	
40	1	2265139050	2265139050	262	
41	1	2265139872	2265139872	255	
42	2	2265148215	2265144724	272	62.3
			2265148215	246	
43	2	2265138431	2265141930	258	75.2
			2265138431	267	
44	2	2265143355	2265143354	275	94.0
			2265143355	266	
45	4	2265143360	2265141713	272	82.2
			2265143359	270	78.5
			2265141714	270	74.8
			2265143360	270	
46	4	2265140657	2265147086	266	78.6
			2265140656	268	76.1
			2265141708	266	74.8
			2265140657	268	
47	2	2265146753	2265138074	261	69.7
			2265146753	263	
48	2	2265138079	2265144437	260	63.2
			2265138079	262	
49	1	2265138680	2265138680	264	
50	1	2265144154	2265144154	275	
51	1	2265143658	2265143658	278	
52	1	2265138917	2265138917	278	
53	1	2265139401	2265139401	271	
54	1	2265138170	2265138170	273	
55	1	2265139953	2265139953	234	
56	1	2265138202	2265138202	241	
57	2	2265147293	2265147441	240	85.4
			2265147293	239	
58	2	2265145034	2265147561	162	100.0
			2265145034	399	
59	2	2265144672	2265142040	310	87.7
			2265144672	311	
60	1	2265146585	2265146585	581	
61	2	2265141539	2265145618	467	73.9
			2265141539	460	
62	1	2265143661	2265143661	257	
63	1	2265137666	2265137666	254	
64	1	2265138238	2265138238	259	
65	2	2265141310	2265146968	257	85.6

			2265141310	257	
66	1	2265140652	2265140652	252	
67	1	2265139751	2265139751	247	
68	1	2265140240	2265140240	253	
69	1	2265138537	2265138537	253	
70	1	2265137746	2265137746	288	
71	2	2265141537	2265145620	290	85.9
			2265141537	290	
72	1	2265139743	2265139743	289	
73	2	2265138180	2265139760	282	64.9
			2265138180	282	
74	1	2265144960	2265144960	287	
75	2	2265145220	2265138058	254	72.8
			2265145220	255	
76	1	2265137555	2265137555	295	
77	3	2265142375	2265145779	293	76.5
			2265138340	302	61.4
			2265142375	298	
78	2	2265146164	2265146163	252	71.4
			2265146164	252	
79	2	2265137957	2265140478	279	64.2
			2265137957	290	
80	3	2265148002	2265146027	291	100.0
			2265147431	87	73.9
			2265148002	294	
81	6	2265142041	2265144677	267	85.8
			2265140630	266	70.8
			2265142043	267	86.9
			2265144322	267	68.9
			2265140977	267	68.5
			2265142041	267	
82	1	2265139063	2265139063	263	
83	1	2265140239	2265140239	268	
84	2	2265142049	2265147103	259	87.5
			2265142049	256	
85	2	2265146461	2265141365	255	83.9
			2265146461	256	
86	1	2265139277	2265139277	261	
87	1	2265139130	2265139130	256	
88	1	2265138209	2265138209	268	
89	2	2265138262	2265146375	276	65.1
			2265138262	275	
90	1	2265144674	2265144674	275	
91	1	2265146165	2265146165	279	
92	1	2265142633	2265142633	269	
93	1	2265139048	2265139048	269	

94	3	2265147814	2265145990	271	89.7
			2265142399	271	81.9
			2265147814	271	
95	1	2265139608	2265139608	310	
96	1	2265146360	2265146360	279	
97	2	2265145767	2265138031	275	62.8
			2265145767	278	
98	1	2265138160	2265138160	272	
99	2	2265144049	2265148334	272	73.9
			2265144049	272	
100	2	2265145502	2265140192	283	76.0
			2265145502	283	
101	1	2265142632	2265142632	291	
102	1	2265140666	2265140666	290	
103	2	2265141373	2265144533	286	87.8
			2265141373	287	
104	1	2265140043	2265140043	286	
105	1	2265143187	2265143187	288	
106	1	2265138241	2265138241	134	
107	1	2265143298	2265143298	502	
108	1	2265145877	2265145877	239	
109	1	2265140336	2265140336	251	
110	1	2265140851	2265140851	310	
111	1	2265140621	2265140621	480	
112	1	2265137983	2265137983	380	
113	1	2265138310	2265138310	251	
114	1	2265138539	2265138539	268	
115	1	2265144874	2265144874	270	
116	1	2265139398	2265139398	269	
117	1	2265137987	2265137987	256	
118	1	2265139830	2265139830	263	
119	1	2265145979	2265145979	272	
120	1	2265140334	2265140334	271	
121	1	2265141296	2265141296	262	
122	1	2265145635	2265145635	261	
123	3	2265138546	2265144058	286	75.2
			2265144059	279	84.6
			2265138546	290	
124	1	2265141433	2265141433	290	
125	1	2265145562	2265145562	309	
126	1	2265140056	2265140056	301	
127	1	2265144722	2265144722	286	
128	1	2265140193	2265140193	275	
129	2	2265145800	2265142194	284	85.6
			2265145800	284	
130	2	2265146099	2265146097	282	69.5

			2265146099	283	
131	2	2265143601	2265140673	296	65.2
			2265143601	287	
132	2	2265137747	2265139528	288	75.6
			2265137747	295	
133	1	2265142707	2265142707	283	
134	1	2265146954	2265146954	274	
135	2	2265146146	2265142544	272	86.1
			2265146146	279	
136	2	2265145040	2265138880	408	60.8
			2265145040	406	
137	1	2265145145	2265145145	253	
138	1	2265148231	2265148231	240	
139	1	2265139813	2265139813	396	
140	1	2265139393	2265139393	297	
141	1	2265145150	2265145150	347	
142	1	2265138605	2265138605	337	
143	1	2265146374	2265146374	282	
144	1	2265144467	2265144467	282	
145	2	2265139222	2265144037	282	64.3
			2265139222	283	
146	1	2265144519	2265144519	278	
147	2	2265147466	2265146830	279	99.6
			2265147466	279	
148	1	2265143569	2265143569	301	
149	1	2265143105	2265143105	402	
150	1	2265139460	2265139460	330	
151	1	2265137916	2265137916	290	
152	2	2265143687	2265141528	285	82.5
			2265143687	285	
153	1	2265140659	2265140659	296	
154	1	2265138123	2265138123	291	
155	1	2265139831	2265139831	246	
156	1	2265144668	2265144668	250	
157	4	2265146882	2265139107	246	69.0
			2265138647	247	76.6
			2265146883	243	81.9
			2265146882	244	
158	1	2265138911	2265138911	179	
159	1	2265137950	2265137950	501	
160	1	2265138877	2265138877	239	
161	1	2265146760	2265146760	191	
162	3	2265142381	2265139040	272	79.0
			2265143156	272	84.1
			2265142381	271	
163	2	2265140729	2265143656	262	71.3

			2265140729	261	
164	1	2265138436	2265138436	258	
165	1	2265138656	2265138656	265	
166	1	2265138491	2265138491	265	
167	1	2265139776	2265139776	264	
168	1	2265143652	2265143652	271	
169	2	2265139723	2265145595	269	73.2
			2265139723	271	
170	1	2265139036	2265139036	270	
171	1	2265139396	2265139396	270	
172	1	2265138927	2265138927	291	
173	1	2265146689	2265146689	279	
174	1	2265140237	2265140237	281	
175	1	2265147119	2265147119	469	
176	1	2265139369	2265139369	242	
177	2	2265143759	2265138141	241	83.7
			2265143759	240	
178	1	2265144511	2265144511	308	
179	1	2265142574	2265142574	296	
180	1	2265140562	2265140562	310	
181	2	2265145759	2265140036	243	69.1
			2265145759	246	
182	1	2265144378	2265144378	245	
183	1	2265139622	2265139622	254	
184	1	2265138242	2265138242	182	
185	1	2265137664	2265137664	273	
186	1	2265144721	2265144721	272	
187	1	2265138538	2265138538	258	
188	1	2265143769	2265143769	266	
189	1	2265140433	2265140433	275	
190	1	2265138681	2265138681	513	
191	1	2265138085	2265138085	295	
192	1	2265138567	2265138567	291	
