# Supplementary material for: The Candidate Phylum Poribacteria by Single-Cell Genomics: New Insights into Phylogeny, Cell-Compartmentation, Eukaryote-Like Repeat Proteins, and Other Genomic Features
Source: PLoS One. 2014 Jan 31;9(1):e87353. doi: 10.1371/journal.pone.0087353 (PMC3909097; doi:10.1371/journal.pone.0087353)
Supplement: Text S1 — Genomic evidence for microcompartments in Poribacteria. (extended description of genomic architecture) (PDF) [file pone.0087353.s012.pdf]

Supplemental Text S1:

Genomic evidence for microcompartments in Poribacteria (extended description of genomic architecture)

Poribacterial BMC shell group A described a conserved area on SAGs 3G, 4CII, and 4E that contained one pfam00936 protein gene and nine to ten additional genes (Fig. 3, Suppl.Table S3). Genes of genomes 3G and 4CII are 100% identical in this area and therefore we only compared the genomes 3G and 4E here. The BMC shell protein (POR3G_00346 / or2319) was followed by a gene encoding for a propanediol utilization protein with hits to pfam06130-PduL (POR3G_00345 & or or2320). The remaining genes of groupA represented mostly hypothetical proteins and genes involved in exopolysaccharide biosynthesis.

The second conserved genomic region (group B) was found on SAGs 3G, 4E, and in fragments on 3A (Fig. 3, Suppl. Table S4). Tandem BMC shell protein genes were found on SAGs 3G and 4E with hits to pfam03319. Directly upstream of the BMC shell protein genes were genes encoding for a two component transcription regulation system with a Che-Y like receiver (or1099, POR3G_4179, POR0191.00000010) and a histidine kinase (or1101, POR3G_04178). This potential regulatory system was interrupted by a hypothetical protein on SAG 4E (or1100). Furthermore, proteins involved in riboflavin biosynthesis are encoded upstream of the regulatory system on SAGs 3G and 4E (or1102-1107, POR3G_04173-04177).

Group C described a region on SAGs 3G and 4E, which was not as well conserved and which encoded for a pfam03319 domain protein (Fig. 3, Suppl. Table S5). Only

two genes in this region showed homologies, namely the BMC shell protein (POR3G_2618, or0690) and a gene encoding for a 5-formyltetrahydrofolate cyclo-ligase (POR3G_2619, or0693), which might participate in folate transformation. In SAG 3G these two genes follow each other directly, while in 4E they are interrupted by two genes encoding for a two component regulatory system (Fig. 3, Suppl. Table S5) as observed in group B but with no homologies to these genes. No further synteny was detected between genomes of group C however SAG 4E encoded for two transposases (or0682 and or0696) in this area.

One additional BMC shell protein was found on SAG 4E (or2241). It was located on the same contig as the one already described in group A. Since there was no synteny with other poribacterial SAGs we defined the area between the BMC shell protein of group A and the additional shell protein on the same genome as group D (Fig. 3, Suppl. Table S6). This gene also encoded for a pfam00936 domain protein and shared homologies of 78.41% and 80.23% to those BMC shell proteins of group A on SAGs 3G and 4E, respectively. Functional information about genes in this area was largely missing. We were able to detect a response regulator with a CheY-like receiver (or2329), as in groups B and C. However, no histidine kinase was found in the genomic neighborhood of this gene, nor were any homologies detected between the response regulator genes of the different groups. Downstream of the second BMC shell protein (or2341) another transposase gene was located (or02347).
